# Supplementary material for: Advancing quantum imaging through learning theory
Source: Nat Commun. 2025 Dec 27;17:1124. doi: 10.1038/s41467-025-67884-1 (PMC12855939; doi:10.1038/s41467-025-67884-1)
Supplement: Supplementary file 1 — Supplementary Information [file 41467_2025_67884_MOESM1_ESM.pdf]

Supplemental Information: Advancing quantum imaging through learning theory

Yunkai Wang,[1, 2, 3, *] Changhun Oh,[4] Junyu Liu,[5, 6] Liang Jiang,[6, †] and Sisi Zhou[1, 2, 3, 7, ‡]

[1] *Perimeter Institute for Theoretical Physics, Waterloo, Ontario N2L 2Y5, Canada.*
[2] *Department of Applied Mathematics, University of Waterloo, Ontario N2L 3G1, Canada.*
[3] *Institute for Quantum Computing, University of Waterloo, Ontario N2L 3G1, Canada.*
[4] *Department of Physics, Korea Advanced Institute of Science and Technology, Daejeon 34141, Republic of Korea.*
[5] *Department of Computer Science, The University of Pittsburgh, Pittsburgh, PA 15260, USA.*
[6] *Pritzker School of Molecular Engineering, The University of Chicago, Chicago, IL 60637, USA.*
[7] *Department of Physics and Astronomy, University of Waterloo, Ontario N2L 3G1, Canada.*

Supplementary Table I: Summary of notations.

**Notation**	**Definition / Meaning**
General setup and probabilities	
$\boldsymbol{\theta}$	Input parameters of the imaging task (e.g., separation L, or moment vector $\vec{x}$).
$p(\boldsymbol{\theta})$	Prior distribution over parameters $\boldsymbol{\theta}$.
$\rho(\boldsymbol{\theta})$	Quantum state on the image plane encoding $\boldsymbol{\theta}$.
M_j	jth POVM element (measurement operator).
$P_j(\boldsymbol{\theta}) = \mathrm{Tr}[\rho(\boldsymbol{\theta})M_j]$	Probability of measurement outcome j.
S	Number of detected samples/photons.
REC (Resolvable Expressive Capacity) formalism	
$f(\boldsymbol{\theta})$	Target function of $\boldsymbol{\theta}$.
$C[f]$	REC of $f(\boldsymbol{\theta})$, measuring accuracy of approximating f with measured features.
$C_T = \sum_k \frac{1}{1+\beta_k^2/S}$	Total REC, effective number of resolvable features at sample size S.
$f_k(\boldsymbol{\theta}) = \sum_m r_{km} P_m(\boldsymbol{\theta})$	kth eigentask.
$\vec{r}_k = [r_{k0}, r_{k1}, r_{k2}, \cdots]$	kth eigenvector defining kth eigentask f_k.
β_k^2	Eigenvalue from REC eigenproblem, sets sample threshold for f_k.
$\hat{\rho}^{(t)} = \mathbb{E}_\theta[\rho(\boldsymbol{\theta})^{\otimes t}]$	Prior-averaged t-copy state.
$D_{kj} = \delta_{kj}\,\mathrm{Tr}[M_k\hat{\rho}^{(1)}]$	Diagonal expectation matrix.
$G_{jk} = \mathrm{Tr}[(M_j \otimes M_k)\,\hat{\rho}^{(2)}]$	Covariance matrix of outcomes.
$V = D - G$	Matrix defining REC eigenproblem.
Imaging model and PSF	
$\lvert u\rangle = a_u^\dagger\lvert 0\rangle$	Single-photon position eigenstate at u.
$\psi(u)$	Point spread function (PSF). Gaussian example: $\psi(u) = \exp\left(-u^2/4\sigma^2\right)/(2\pi\sigma^2)^{1/4}$.
σ	Width of the PSF.
$I(u)$	Normalized source intensity distribution.
Two incoherent point sources	
L	Separation between two point sources.
$u_1 = -L/2,\ u_2 = +L/2$	Coordinates of the sources.
$\rho(L) = \frac{1}{2}(\lvert\psi_1\rangle\langle\psi_1\rvert + \lvert\psi_2\rangle\langle\psi_2\rvert)$	State of two incoherent points.
$p(L) = \frac{1}{\sqrt{2\pi}\gamma} e^{-L^2/2\gamma^2}$	Gaussian prior for separation L.
$\alpha = \gamma/\sigma$	Small parameter in two-point source analysis.
$E_x = \lvert x\rangle\langle x\rvert$	Projector onto position x.
$M_0 = \lvert\phi_0\rangle\langle\phi_0\rvert,\ M_1 = I - M_0$	Binary SPADE POVM.
$\phi_0(u) = (2\pi\sigma^2)^{-1/4} e^{-u^2/4\sigma^2}$	Fundamental Gaussian mode.
Single compact source (moments)	
L	Source size.
$\alpha = L/\sigma$	Small parameter for compact source.
$x_n = \int du\, I(u) \left(\frac{u-u_0}{L}\right)^n$	nth normalized moment.
$\vec{x} = [x_0, x_1, \ldots]$	Moment vector.

* ywang10@perimeterinstitute.ca
† liangjiang@uchicago.edu
‡ sisi.zhou26@gmail.com

Notation	Definition / Meaning
$\rho = \sum_{k=0}^{\infty} x_k \rho^{(k)}$	Expansion of state in PSF derivatives.
$\|\psi^{(m)}\rangle$	Basis vector defined by the mth derivative of the PSF.
$\|b_l\rangle$	Basis obtained via Gram–Schmidt orthogonalization of the set $\{\|\psi^{(m)}\rangle\}_{m=0,1,2,\cdots}$.
$\|\phi_{n,\pm}\rangle = (\|b_n\rangle \pm \|b_{n+1}\rangle)/\sqrt{2}$	Basis vectors of the projective measurement used in SPADE.
$P_{n,\pm}$	Outcome probabilities.
$c_{nm},\, c^{\pm}_{nm}$	Expansion coefficients in probabilities.
Multiple compact sources	
Q	Number of compact sources.
$I_q(u)$	Intensity of the qth source.
u_q	Centroid of the qth source.
L_q	Size of the qth source.
$x_{n,q} = \int du\, I_q(u) \left(\frac{u-u_q}{L_q}\right)^n$	nth moment of the qth source.
$\|\psi_q^{(m)}\rangle$	Basis vector defined by the mth derivative of the PSF centered at u_q.
$\|b_{q,m}\rangle$	Basis obtained via Gram-Schmidt orthogonalization of set $\{\left\|\psi_q^{(m)}\right\rangle\}_{m=0,1,2,\cdots,\infty}$
$\|\phi_{q,m,\pm}\rangle = \frac{1}{\sqrt{2}}(\|b_{q,m}\rangle \pm \|b_{q,m+1}\rangle)$	Basis vectors of the projective measurement used in separate SPADE method.
$\|b_j^{(l)}\rangle$	Basis obtained via Gram–Schmidt orthogonalization of the set $\{\|\psi_q^{(m)}\rangle\}_{m=0,1,2,\cdots,\, q=1,2,\cdots,Q}$.
$\left\|\phi_{j\pm}^{(l)}\right\rangle = \frac{1}{\sqrt{2}}\left(\left\|b_j^{(l)}\right\rangle \pm \left\|b_j^{(l+1)}\right\rangle\right)$	Basis vectors of the projective measurement used in orthogonalized SPADE method.
Demonstrative examples	
$\eta(u)$	Indicator function used to randomly generate intensity distributions.
$\zeta(u)$	The function that specifies the positions of compact sources.
d	Region size containing all compact sources.
$\alpha = 0.1,\ \sigma = 1$	Example numerical parameters used in demonstrations.
$P_m(j)$	Probability of the jth measurement outcome for the mth intensity distribution.
$\vec{\xi}_m = [\xi_{0m}, \xi_{1m}, \ldots, \xi_{\mathcal{K}m}]$	Eigentask vector for the mth distribution, truncated at order $\mathcal{K}$.
$\vec{r}_k = [r_{k0}, r_{k1}, r_{k2}, \cdots]$	kth eigenvector defining kth eigentask.
$\xi_{km} = \sum_j r_{kj} P_m(j)$	kth eigentask for the mth distribution.
$\mathcal{K}$	Truncation order of the eigentask vector.
Auxiliary / supplemental symbols	
F	Fisher information matrix.
λ_k	Generalized eigenvalue, related to β_k^2.
$W = D^{-1/2} G D^{-1/2}$	Symmetrized matrix used in perturbation analysis.
C_n	Coefficient vectors in α-expansions.
$d_k,\quad g_{ij}$	Prior-dependent quantities: $d_k = \int d\vec{x}\, p(\vec{x})\, x_k$, $g_{ij} = \int d\vec{x}\, p(\vec{x})\, x_i x_j$.
z_n	Orthonormal basis vectors introduced for convenience in perturbative analysis.
Π_m	Projectors for block decomposition in perturbation theory.
$\vec{y}_k$	Auxiliary vectors appearing as eigenvectors of an equivalent form of the REC eigenproblem.

Supplementary Note 1. PRELIMINARY

A. Review of quantum-metrology-inspired superresolution

The Fisher information matrix F provides a lower bound on the variance $\Sigma_{\vec{\theta}}$ associated with estimating unknown parameters $\vec{\theta} = [\theta_1, \theta_2, \cdots, \theta_n]$, such that $\Sigma_{\vec{\theta}} \geq F^{-1}$ [1, 2]. Quantum-metrology-inspired superresolution leverages the insight that a more carefully designed measurement can yield significantly higher Fisher information compared to direct imaging, when the size of the source is much smaller than the Rayleigh limit of the imaging system. Ref. [3] highlighted that, when estimating the separation between two point sources, the Fisher information from direct imaging approaches zero as the separation between the sources decreases to zero, which indicates the Rayleigh't limit. However, the Fisher information for a spatial mode demultiplexing (SPADE) measurement onto Hermite-Gaussian modes remains constant, suggesting that it is possible to circumvent Rayleigh's criterion by carefully designing the measurement strategy.

Later on, this discussion is extended to consider a general source within the Rayleigh limit. We now review the discussion in Ref. [4] using our notation in more detail for later use. A general incoherent source can be modeled as

$$\rho = \int du du_1 du_2 I(u)\psi(u-u_1) |u_1\rangle \langle u_2| \psi(u-u_2), \tag{1}$$

where $|u\rangle = a_u^\dagger |0\rangle$ is the single photon state at position u, $\psi(u)$ is the PSF whose width is quantified as σ, such as $\psi(u) = \exp\big(-u^2/4\sigma^2\big)/(2\pi\sigma^2)^{1/4}$. Assume the normalized source intensity $I(u)$ is confined within the interval $[-L/2, L/2]$, define $\alpha = L/\sigma \ll 1$, we expand the PSF

$$\psi(u_1-u) = \sum_{n=0}^{\infty} \left(\frac{\partial^n \psi(u_1-u)}{\partial u^n}\bigg|_{u=u_0} \frac{L^n}{n!} \right) \left(\frac{u-u_0}{L}\right)^n = \sum_{n=0}^{\infty} \psi^{(n)}(u_1) \left(\frac{u-u_0}{L}\right)^n. \tag{2}$$

We can then get

$$\begin{aligned} \rho &= \sum_{m,n=0}^{\infty} \left(\int du I(u) \left(\frac{u-u_0}{L}\right)^{m+n} \right) \left(\int du_1 \psi^{(m)}(u_1) |u_1\rangle \right) \left(\int du_2 \psi^{(m)}(u_2) \langle u_2| \right) \\ &= \sum_{m,n=0}^{\infty} x_{m+n} \left|\psi^{(m)}\right\rangle \left\langle \psi^{(n)}\right| = \sum_{k=0}^{\infty} x_k \rho^{(k)}, \end{aligned} \tag{3}$$

where $\rho^{(k)} = \sum_{m+n=k} \left|\psi^{(m)}\right\rangle \left\langle\psi^{(n)}\right|$. We aim to construct a measurement such that, when estimating higher-order moments, the lower-order terms of α, which introduce significant noise, can be canceled. This can be achieved by constructing an orthonormal measurement basis $|b_l\rangle_l$ through the Gram-Schmidt procedure such that

$$a_{ml} = \left\langle \psi^{(m)} \middle| b_l \right\rangle \begin{cases} = 0 & m \le l-1 \\ \neq 0 & m \ge l \end{cases} \tag{4}$$

The measurement is then constructed as

$$\left\{ \frac{1}{2} |\phi_{i,\pm}\rangle \langle \phi_{i,\pm}|, \frac{1}{2} |b_0\rangle \langle b_0| \right\}, \tag{5}$$

$$|\phi_{i,\pm}\rangle = (|b_i\rangle \pm |b_{i+1}\rangle)/\sqrt{2}, \quad i = 0, 1, 2, \cdots \tag{6}$$

The probability of projecting onto state $|\phi_{n,\pm}\rangle$ is

$$\begin{aligned} P_0 &= \frac{1}{2} \sum_{m=0}^{\infty} x_m \rho_{0,m,0} = \sum_{m=0}^{\infty} c_m x_m \alpha^m \\ P_{n,\pm} &= \frac{1}{4} \sum_{m=2n}^{\infty} x_m \rho_{n,m,n} + \frac{1}{4} \sum_{m=2n+2}^{\infty} x_m \rho_{(n+1),m,(n+1)} \pm \frac{1}{4} \sum_{m=2n+1}^{\infty} x_m (\rho_{n,m,(n+1)} + \rho_{(n+1),m,n}) \\ &= \sum_{m \ge 2n} c_{nm}^{\pm} x_m \alpha^m, \quad n = 0, 1, 2, 3, \cdots \\ \rho_{n,m,l} &= \langle b_n| \rho^{(m)} |b_l\rangle \sim \Theta(\alpha^m), \end{aligned} \tag{7}$$

where we can find each coefficients c_{nm}.

As suggested above, the key feature in the construction of the superresolution method is that we can have the probability distribution $P_{n,\pm}$ starting from the $\Theta(\alpha^{2n})$ order. This is crucial because, when estimating higher-order moments, these moments appear as the coefficients of higher-order terms in the probability distribution. For example, when we want to estimate the moment x_2, this information is encoded in $P_0 = c_0 x_0 + c_1 x_1 \alpha + c_2 x_2 \alpha^2 + \cdots$, $P_{0,\pm} = c_{00}^{\pm} x_0 + c_{01}^{\pm} x_1 \alpha + c_{02}^{\pm} x_2 \alpha^2 + \cdots$ as well, but these contain lower-order terms of α such as $c_0 x_0$, $c_1 x_1 \alpha$, etc. In contrast, the probability distribution $P_{1,\pm} = c_{12}^{\pm} x_2 \alpha^2 + c_{13}^{\pm} x_3 \alpha^3 + \cdots$ has x_2 in the leading-order term. Intuitively, the lower-order terms of α serve as noise in estimating higher-order moments, while the term containing x_2 constitutes the signal. As $\alpha \to 0$, the signal-to-noise ratio in P_0 and $P_{0,\pm}$ becomes very poor due to the dominance of these noise terms. In contrast, in $P_{1,\pm}$, where lower-order terms are absent, the signal-to-noise ratio is significantly improved.

This improvement is explicitly quantified by the Fisher information. The Fisher information contributed by the basis $|\phi_{n,\pm}\rangle$ is

$$F^{(n)}_{x_i x_j} = \begin{cases} \Theta(\alpha^{i+j-2n}) & , \quad i,j \geq 2n \\ 0 & , \quad i < 2n \quad \text{or} \quad j < 2n \end{cases} \tag{8}$$

where $i, j, n = 0, 1, 2, \cdots$. Adding the contribution of all basis $|\phi_{n,\pm}\rangle$, the total Fisher information $F = \sum_n F^{(n)}$ is given by

$$F_{x_i x_j} = \Theta(\alpha^{i+j-2\lfloor \min\{i,j\}/2 \rfloor}). \tag{9}$$

In particular, the diagonal elements $F_{x_i x_i}$ scale as $\Theta(\alpha^0), \Theta(\alpha^2), \Theta(\alpha^2), \Theta(\alpha^4), \Theta(\alpha^4), \cdots$ for $i = 0, 1, 2, 3, 4, \cdots$. Note that the scaling of the Fisher information in the above equation differs from that in Ref.[4] by a factor of α^2. This discrepancy arises because we define the moment as $x_k = \int du I(u)(u - u_0)^k / L^k$, while in Ref.[4], the moment was defined as $M_k = (\int du I(u)(u - u_0)^k)^{1/k}$. Here, x_k is independent of α, whereas $M_k \propto \alpha$, leading to the factor of α^2 difference in the Fisher information.

For a direct imaging approach, we simply project onto the state $|u\rangle \langle u|$ to estimate the intensity at each position u on the image plane, with the probability given by

$$P_n = \sum_{m=0}^{\infty} c_{nm} x_m \alpha^m, \quad n = 0, 1, 2, 3, \cdots \tag{10}$$

Note that in practical detection scenarios, it is not feasible to estimate each spatial mode individually as $|u\rangle \langle u|$. Instead, we measure discretized pixels, represented as $M_n = \int_{X_n - l/2}^{X_n + l/2} dx \, |x\rangle \langle x|$, where l is the size of each pixel in the direct imaging, X_n is the centroid of each pixel. Consequently, we will use a discretized version of direct imaging for both analytical and numerical discussions. And we can calculate the FI of estimating x_n as

$$F_{x_i x_j} = \Theta(\alpha^{i+j}), \tag{11}$$

which is much lower compared to the measurement constructed in Eq. 9. Specifically, the diagonal elements scale as $F_{x_i x_i} = \Theta(\alpha^0), \Theta(\alpha^2), \Theta(\alpha^4), \Theta(\alpha^6), \Theta(\alpha^8), \cdots$ for $i = 0, 1, 2, 3, 4, \cdots$. This indicates that sensitivity can be improved by carefully designing the measurement strategy.

The measurement scheme from Ref. [4] works universally for any PSF. For the Gaussian PSF $\psi(u) = \exp\left(-u^2/4\sigma^2\right)/(2\pi\sigma^2)^{1/4}$, the scheme from Ref. [4] is the projective measurement $\left\{\frac{1}{4}(|b_i\rangle \pm |b_{i+1}\rangle)(\langle b_i| \pm \langle b_{i+1}|), \frac{1}{2} |b_0\rangle \langle b_0|\right\}_{i=0,1,2,\cdots}$ as in Eq. 5, where $|b_q\rangle = \int du \, \phi_q(u) \, |u\rangle$ and $\phi_q(u) = (1/2\pi\sigma^2)^{1/4}(1/\sqrt{2^q q!}) H_q(\frac{x}{\sqrt{2}\sigma}) \exp\left(-\frac{u^2}{4\sigma^2}\right)$, with H_q as the Hermite polynomial. Note that the SPADE measurement first used in Ref. [3] is the projective measurement $\{|b_q\rangle \langle b_q|\}_{q=0,1,2,3,\cdots}$ when PSF is Gaussian, the measurement scheme from Ref. [4] is the projective measurement $\left\{\frac{1}{4}(|b_i\rangle \pm |b_{i+1}\rangle)(\langle b_i| \pm \langle b_{i+1}|), \frac{1}{2} |b_0\rangle \langle b_0|\right\}_{i=0,1,2,\cdots}$. For simplicity, however, we still refer to the measurement from Ref. [4] for any PSF as the SPADE method throughout the discussion. However, all the analytical results presented in this work apply to any PSF and to the measurement approach from Ref. [4].

We briefly summarize related theoretical works for comparison with our results. Sorelli et al. [5] propose a useful estimator for the superresolution problem. However, their work focuses on a simple scenario—two point sources—and remains within the conventional parameter-estimation framework based on Fisher information, targeting a single well-defined parameter: the separation distance. In contrast, we address the imaging of multiple general compact sources with more complex structures using a quantum learning approach. Rather than estimating specific parameters like moments, our method identifies which features can be reliably estimated, given prior knowledge and the measurement model in the finite-sample regime. The resulting eigentasks are problem-dependent. Our framework can tackle complex tasks by informing downstream analysis strategies, making it more versatile and practically relevant than the approach of Sorelli et al. Grace et al. [6] focus on estimating the separation and centroid of two point sources of equal strength, framed as a simple parameter estimation problem. In contrast, our approach adopts a quantum learning framework and addresses more general imaging scenarios, where the source can have structures far more complex than just two point sources.

B. Physical neural networks

In parametric quantum circuit learning [7–17], a widely studied paradigm in quantum machine learning, one prepares an initial state and applies a sequence of parameterized unitaries $U(\vec{\omega})$, where the gate operations depend on tunable

parameters $\vec{\omega}$. The input parameters $\boldsymbol{\theta}$ are first embedded through a data-encoding unitary $U_{\rm enc}(\boldsymbol{\theta})$, so that the overall state is $|\psi(\boldsymbol{\theta},\vec{\omega})\rangle = U(\vec{\omega})U_{\rm enc}(\boldsymbol{\theta})|0\rangle$. Measuring an observable O at the output yields an expectation value $f(\boldsymbol{\theta},\vec{\omega}) = \langle 0|U_{\rm enc}^\dagger(\boldsymbol{\theta})U^\dagger(\vec{\omega})OU(\vec{\omega})U_{\rm enc}(\boldsymbol{\theta})|0\rangle$, which defines a family of functions parameterized jointly by the input $\boldsymbol{\theta}$ and the trainable parameters $\vec{\omega}$. By iteratively updating $\vec{\omega}$ during training, the expectation values can be steered toward desired outcomes, enabling the circuit to approximate target functions of $\boldsymbol{\theta}$ and thereby address a wide range of tasks in optimization, learning, and quantum simulation. The expressive power of the model is governed by the architecture of $U(\vec{\omega})$—including its depth and connectivity—as well as by the choice of data-encoding map $U_{\rm enc}(\boldsymbol{\theta})$ and measurement observables.

Physical neural networks (PNNs) can be regarded as special instances of parametric quantum circuit learning [18–30]. Unlike variational quantum circuits, PNNs contain no tunable gates within the physical evolution itself. Once the physical setup is specified, the dynamics are entirely fixed, so the parameters $\vec{\omega}$ and the corresponding unitary $U(\vec{\omega})$ that would normally be optimized in a parametric quantum circuit are predetermined. The evolution then produces a fixed set of measurement features $\eta_j(\boldsymbol{\theta}) = \langle 0|U_{\rm enc}^\dagger(\boldsymbol{\theta})U^\dagger(\vec{\omega})O_jU(\vec{\omega})U_{\rm enc}(\boldsymbol{\theta})|0\rangle$, where $\boldsymbol{\theta}$ denotes the input parameters. Learning takes place only at the output stage, where the features $\eta_j(\boldsymbol{\theta})$ are linearly combined with adjustable weights W_j to realize the desired task, $f(\boldsymbol{\theta}) = \sum_j W_j\eta_j(\boldsymbol{\theta})$. In this sense, PNNs represent a limiting case of parametric quantum circuits: the mapping from input to features is dictated entirely by the fixed physics of the system, while optimization is restricted to the output weights W_j.

An important advantage of the PNN approach is that it avoids the need to optimize over a large number of variational parameters within the circuit. In standard parametric quantum circuit learning, training can be hindered by barren plateaus, slow convergence, and the heavy computational cost of navigating a high-dimensional parameter space [31–33]. PNNs circumvent these challenges entirely: the physical evolution is fixed by the setup, and learning reduces to an optimization over only the output weights. This makes training efficient and scalable with classical resources, while the underlying physics directly supplies a structured and interpretable feature map. Moreover, owing to the intrinsic complexity of the physical dynamics, PNNs may naturally generate highly expressive feature maps, offering the potential for computational advantages over classical learning models. Taken together, these features make PNNs a robust and data-efficient framework for quantum learning, with particular promise for near-term applications where experimental control is limited and simple, reliable training procedures are essential.

In the imaging context, the role of the PNN is realized by the optical system together with its measurement scheme. Information about the source is encoded into a quantum state that arrives at the imaging plane, evolves through the imaging channel, and is subsequently probed by measurements that generate the feature functions. Imaging tasks such as object recognition, classification, or image reconstruction can then be modeled as learning problems within the PNN framework.

PNNs share the same high-level structure as parametric quantum circuit learning—consisting of state encoding, physical evolution, and measurement—but they differ in a crucial respect: the internal dynamics are fixed once the physical setup is chosen, so the expressive capacity of the feature map is determined entirely by the physics of the system. This raises the central question of which functions, or equivalently which learning tasks, can be supported by a given PNN architecture. The resolvable expressive capacity (REC) formalism established in Ref. [30] provides a natural framework to address this question, offering a quantitative characterization of the expressive capacity and revealing how the approximability of target functions and the associated sample complexity are governed by the eigentasks of the system.

C. Resolvable expressive capacity

We will use the tools of REC formalism introduced in Ref. [30]. If our goal is approximate a function $f(\boldsymbol{\theta})$, define the capacity

$$C[f] = 1 - \min_{W} \frac{\mathbb{E}_{\boldsymbol{\theta}}\left[\mathbb{E}_{\mathcal{X}}\left[\left(\sum_{k=0}^{K-1} W_k\bar{\eta}_k(\boldsymbol{\theta}) - f(\boldsymbol{\theta})\right)^2\right]\right]}{\int f^2(\boldsymbol{\theta})p(\boldsymbol{\theta})d\boldsymbol{\theta}}, \tag{12}$$

where we take the expectation value for the output sample $\mathcal{X}$ and the prior distribution $p(\boldsymbol{\theta})$, where $f(\boldsymbol{\theta})$ is approximated by a linear combination of measured functions $\bar{\eta}_k(\boldsymbol{\theta})$. $C[f]$ takes values between 0 and 1, representing the relative error of approximating the target function $f(\boldsymbol{\theta})$ as a linear combination of the measured features $\bar{\eta}_j(\boldsymbol{\theta})$. Here, $\bar{\eta}_j(\boldsymbol{\theta})$ denotes the measured features, which approximate the actual features $\eta_j(\boldsymbol{\theta})$.

The value of $C[f]$ depends on two aspects. Firstly, whether $f(\boldsymbol{\theta})$ can be well approximated by the features $\eta_j(\boldsymbol{\theta})$. Note not all functions of the input parameters $\boldsymbol{\theta}$ can be approximated as a linear combination of the features $\eta_j(\boldsymbol{\theta})$. The spanned linear subspace by all the features $\eta_j(\boldsymbol{\theta})$ actually quantifies the capability of the imaging system. A

larger subspace spanned by $\eta_j(\boldsymbol{\theta})$ means we can approximate more functions of the input parameters $\boldsymbol{\theta}$. And which functions lie in this subspace gives the information of what can be obtained using this imaging system. In the case that $f(\boldsymbol{\theta})$ is not in this subspace spanned by $\eta_j(\boldsymbol{\theta})$, the value of $C[f]$ will always significantly deviate from 1 even if we increase $S \to \infty$. In the worst case, we can at least choose $W_j = 0$ for $\forall j$, which gives $C[f] = 0$. So, if we cannot well approximate the target function, $C[f]$ takes values not close to 1. Secondly, in the case that $f(\boldsymbol{\theta})$ is in this subspace spanned by $\eta_j(\boldsymbol{\theta})$, this means that with the actual value of features $\eta_j(\boldsymbol{\theta})$, we have $\sum_j W_j \bar{\eta}_j(\boldsymbol{\theta}) - f(\boldsymbol{\theta}) \approx 0$. Furthermore, since $\bar{\eta}_j(\boldsymbol{\theta})$ is the measured feature as an approximation of the actual feature $\eta_j(\boldsymbol{\theta})$, when the sample number S is not sufficiently large, the deviation of $\bar{\eta}_j(\boldsymbol{\theta})$ from $\eta_j(\boldsymbol{\theta})$ will also cause some error. But when $S \to \infty$, we have a very good approximation. In this case, $C[f] \approx 1$. The mean squared error loss serves as an effective approximation of the first term in the Taylor expansion for a wide range of nonlinear post-processing and non-quadratic loss functions [30].

The total REC is defined as $C_T = \sum_{l=0}^{\infty} C[f_l]$, where $\{f_l\}_l$ represents a complete and orthonormal set of basis functions in the space of all functions of input parameters $f(\boldsymbol{\theta})$. Note that this set of basis functions has infinitely many elements, as the space of functions of $\boldsymbol{\theta}$ is infinite-dimensional. However, the calculation of $C_T = \sum_{l=0}^{\infty} C[f_l]$ can be reduced to just the eigentasks defined based on the following eigenvalue problem.

$$C_T = \mathrm{Tr}\left(\left(G + \frac{1}{S}V\right)^{-1} G\right) = \sum_{k=0}^{K-1} \frac{1}{1+\beta_k^2/S}, \tag{13}$$

$$\begin{gathered} D_{kk} = \mathrm{Tr}\left\{\hat{M}_k \hat{\rho}^{(1)}\right\}, \quad G_{jk} = \mathrm{Tr}\left\{\left(\hat{M}_j \otimes \hat{M}_k\right)\hat{\rho}^{(2)}\right\}, \quad \hat{\rho}^{(t)} = \int \rho(\boldsymbol{\theta})^{\otimes t} p(\boldsymbol{\theta}) d\boldsymbol{\theta}, \\ V = D - G, \quad V r_k = \beta_k^2 G r_k. \end{gathered} \tag{14}$$

where $p(\boldsymbol{\theta})$ describes our prior knowledge about the input, $\rho(\boldsymbol{\theta})$ is the quantum state encoded with input data $\boldsymbol{\theta}$, S is the number of samples, $\{M_k\}$ is a set of K POVM elements. The total REC C_T describes the capability of approximating arbitrary functions $f(\boldsymbol{\theta})$ using $\bar{\eta}_k(\boldsymbol{\theta})$. In this work, $\bar{\eta}_k(\boldsymbol{\theta})$ corresponds to the observed frequency of occurrence of the kth measurement outcome and $\mathbb{E}_{\mathcal{X}}[\bar{\eta}_k(\boldsymbol{\theta})] = \eta_k(\boldsymbol{\theta}) = P_k(\boldsymbol{\theta}) = \mathrm{tr}(\rho(\boldsymbol{\theta})M_k)$. A larger C_T, or smaller β_k^2, indicates better learning performance. Note that this theoretical framework incorporates only $\hat{\rho}^{(t=1,2)}$. This is because $C[f]$ is defined based on the square error, so when deriving the equation, only the expectation and covariance of $\bar{\eta}_k(\boldsymbol{\theta})$ are involved. Since we only need the expectation and covariance of the probability distribution, $\hat{\rho}^{(t=1,2)}$ is sufficient.

The eigenvectors r_k determines the eigentasks

$$f_k(\boldsymbol{\theta}) = \sum_j r_{kj}\eta_j(\boldsymbol{\theta}), \tag{15}$$

where $\eta_j(\boldsymbol{\theta}) = \mathbb{E}_{\mathcal{X}}[\bar{\eta}_j(\boldsymbol{\theta})]$ is probability of obtaining the kth measurement outcome. The REC of each eigentask is given by $C[f_k(\boldsymbol{\theta})] = 1/(1+\beta_k^2/S)$. Intuitively, the total REC C_T accounts for all possible basis functions. In practice, however, most basis functions contribute negligibly to C_T, and it suffices to consider only the eigentasks f_k constructed from the vectors r_k that have significant contributions. Importantly, in the finite-sample regime, the relevance of each eigentask is well quantified by its associated eigenvalue β_k^2. In the actual experiment, the direct measurement results yield $\bar{\eta}_j(\boldsymbol{\theta})$. In general, the information obtained from each η_j may not be independent. On the other hand, the eigentasks f_k are orthonormal in the sense of

$$\mathbb{E}_{\boldsymbol{\theta}}[f_k(\boldsymbol{\theta}) f_j(\boldsymbol{\theta})] = \delta_{kj}. \tag{16}$$

Intuitively, these can be interpreted as the "independent" tasks with learning precision quantified by β_k^2. The calculation of eigentasks can give us a sense of what function is hard to be estimated for our measurement device.

Supplementary Note 2. TWO-POINT SOURCES

In this section, we give more details about the calculation of imaging two-point sources using direct imaging and the binary SPADE method proposed in Ref. [3]. For the binary SPADE method, the POVM can be written as $M_0 = |\phi_0\rangle\langle\phi_0|$, $M_1 = I - M_0$, $|\phi_0\rangle = \int du \phi_0(u)|u\rangle$, $\phi_0(u) = \frac{1}{(2\pi\xi^2)^{1/4}}\exp\left(-\frac{u}{4\xi^2}\right)$. We can then calculate the matrix elements of D, G for the eigenvalue problem $V = D - G$, $Vr_k = \beta_k^2 G r_k$.

$$D_{00} = \mathrm{tr}\left(M_0\rho^{(1)}\right) = \frac{4\xi\sigma}{\sqrt{(\xi^2+\sigma^2)(\gamma^2+4\xi^2+4\sigma^2)}}, \quad D_{11} = 1 - D_{00}, \tag{17}$$

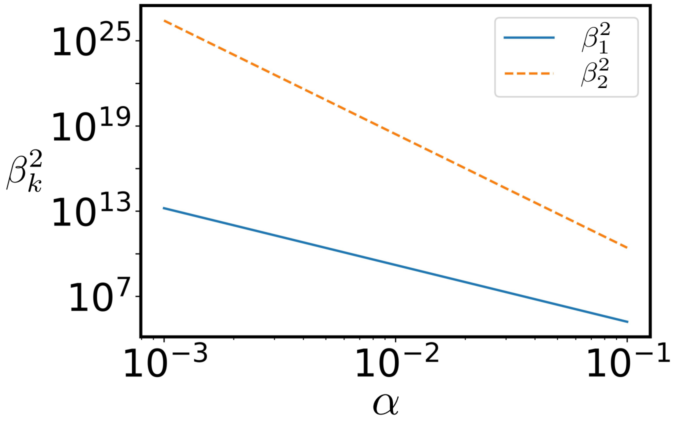

Supplementary Figure 1. Scaling of the eigenvalue $\beta^2_{1,2,3}$ as a function of α for direct imaging of two-point sources.

$$\begin{aligned}
G_{00} &= \mathrm{tr}\Big((E_0 \otimes E_0)\rho^{(2)}\Big) = \frac{4\sqrt{2}\sigma^2\xi^2}{(\sigma^2+\xi^2)^{3/2}\sqrt{\gamma^2+2\xi^2+2\sigma^2}},\\
G_{01} &= G_{10} = \mathrm{tr}\Big((E_0 \otimes E_1)\rho^{(2)}\Big) = \mathrm{tr}\Big((E_0 \otimes I)\rho^{(2)}\Big) - \mathrm{tr}\Big((E_0 \otimes E_0)\rho^{(2)}\Big) = D_{00} - G_{00},\\
G_{11} &= \mathrm{tr}\Big((E_1 \otimes E_1)\rho^{(2)}\Big) = \mathrm{tr}\Big(\rho^{(2)}\Big) - \mathrm{tr}\Big((I \otimes E_0)\rho^{(2)}\Big) - \mathrm{tr}\Big((E_0 \otimes I)\rho^{(2)}\Big) + \mathrm{tr}\Big((E_0 \otimes E_0)\rho^{(2)}\Big) = 1 - 2D_{00} + G_{00},
\end{aligned} \tag{18}$$

$$D = \begin{bmatrix} D_{00} & 0 \\ 0 & D_{11} \end{bmatrix}, \quad G = \begin{bmatrix} G_{00} & G_{01} \\ G_{10} & G_{11} \end{bmatrix}, \tag{19}$$

which then gives us the eigenvalues

$$\begin{aligned}
\beta_0^2 &= 0,\\
\beta_1^2 &= \frac{-\sqrt{2}\xi\sigma(\gamma^2+4(\xi^2+\sigma^2)) + \sqrt{\xi^2+\sigma^2}\sqrt{\gamma^2+2(\xi^2+\sigma^2)}\sqrt{(\xi^2+\sigma^2)(\gamma^2+4(\xi^2+\sigma^2))}}{\xi\sigma(4\sqrt{2}\xi^2+\sqrt{2}(\gamma^2+4\sigma^2) - 4\sqrt{\xi^2+\sigma^2}\sqrt{\gamma^2+2(\xi^2+\sigma^2)})}.
\end{aligned} \tag{20}$$

If we assume $\gamma \ll \sigma, \xi$ and define $\alpha = \gamma/\sigma$, we have

$$\beta_1^2 = \frac{16(\xi-\sigma)^2(\xi^2+\sigma^2)^2}{\xi\sigma\gamma^4} + \frac{2(\xi^2+\sigma^2)(-8\xi\sigma+5(\xi^2+\sigma^2))}{\xi\sigma\gamma^2} + \frac{3}{8}\left(-4+\frac{3\xi}{\sigma}+\frac{3\sigma}{\xi}\right) - \frac{\gamma^2}{64(\xi\sigma)} + O(\alpha^4). \tag{21}$$

The first-order term $\Theta(\alpha^{-4})$ can be made to vanish if we choose $\xi = \sigma$. With this choice, the leading order of β_1^2 becomes proportional to α^{-2}.

We may want to compare this with the direct imaging case where we directly project onto each spatial mode $\{E_x = |x\rangle\langle x|\}_x$. We again calculate the matrix elements as

$$D_{xx} = \mathrm{tr}\Big(E_x\rho^{(1)}\Big) = \frac{\sqrt{2/\pi}}{\sqrt{\gamma^2+4\sigma^2}}\exp\big[-2x^2/(\gamma^2+4\sigma^2)\big], \tag{22}$$

$$G_{xy} = \mathrm{tr}\Big((E_x \otimes E_y)\rho^{(2)}\Big) = \frac{\exp\Big(-\frac{x^2+y^2}{2\sigma^2}\Big)}{2\sqrt{2}\pi\gamma\sigma^2\sqrt{\frac{2}{\gamma^2}+\frac{1}{\sigma^2}}}\left[\exp\left(\frac{\gamma^2(x-y)^2}{4\sigma^2(\gamma^2+2\sigma^2)}\right) + \exp\left(\frac{\gamma^2(x+y)^2}{4\sigma^2(\gamma^2+2\sigma^2)}\right)\right]. \tag{23}$$

It is possible to numerically find β_k^2 as shown in Supplementary Figure 1. We again choose $\alpha = \gamma/\sigma$. In this case, the smallest eigenvalue $\beta_0^2 = \Theta(1)$, followed by $\beta_1^2 = \Theta(\alpha^{-4})$ and $\beta_2^2 = \Theta(\alpha^{-8})$. Values of β_k^2 exceeding $\sim 10^{15}$ in Supplementary Figure 1 are interpolated using a straight-line fit at larger α to mitigate numerical precision loss; this interpolation is applied to all β_k^2–α plots. More details about the numerical calculation can be found in Supplementary Note 6.

We use the prior distribution $p(L) = \frac{1}{\sqrt{2\pi}\gamma}\exp\left(-\frac{L^2}{2\gamma^2}\right)$, which reflects the assumption that the separation L is much smaller than the PSF width σ. This choice is motivated by the fact that the advantage of superresolution over direct imaging becomes significant only in the Rayleigh limit where $L \ll \sigma$. For a fair comparison, both methods are given the same prior. The prior serves as a probability distribution over possible separations L, making the overall precision—as quantified by the total REC—a weighted average of local precisions at each value of L. This is particularly relevant in subdiffraction imaging, where precision varies strongly with L: direct imaging performs poorly for small L, while superresolution maintains high precision. Although superresolution still performs well for larger L, its relative advantage over direct imaging diminishes. Conventional parameter estimation frameworks based on Fisher information assess precision locally at the true parameter value, which is typically unknown. In contrast, the quantum learning approach incorporates the prior directly and defines precision globally across the parameter space. This makes it more suitable when the parameter may vary over a range of values. Finally, the prior alone does not guarantee accurate estimation. The REC formalism, which measures relative error. While the prior implies $L \ll \sigma$, this does not ensure low relative error. In practice, it is easy to recognize that two sources are too close to be resolved by direct imaging, but extracting precise subdiffraction information is much more challenging—and better accomplished with superresolution. Therefore, we believe this prior is a well-motivated choice for the present discussion.

Supplementary Note 3. A SINGLE COMPACT SOURCE (GENERALLY DISTRIBUTED SOURCE WITHIN THE RAYLEIGH LIMIT)

A. Direct imaging of a single compact source

We model a general incoherent source as

$$\rho = \sum_{k=0}^{\infty} x_k \rho^{(k)}, \tag{24}$$

where $\rho^{(k)} = \Theta(\alpha^k)$, which depends on the choice of PSF as detailed in Supplementary Note 1 A and moments vector $\vec{x} = [x_0, x_1, \cdots,]^T$ is our input data, i.e. $\boldsymbol{\theta} = \vec{x}$. For direct imaging, we directly project onto each position $|u\rangle$, and the probability distribution for the direct imaging is given in the form of

$$P_n = \sum_{m=0}^{\infty} c_{nm} x_m \alpha^m, \quad n = 0, 1, 2, 3, \cdots \tag{25}$$

Again, we measure $M_n = \int_{X_n - l/2}^{X_n + l/2} du\, |u\rangle\langle u|$, a discretized version of direct imaging for both analytical and numerical discussions. Assume we have the prior knowledge about the moments $\vec{x} = [x_0, x_1, x_2, \cdots]$ described by $p(\vec{x})$. The prior distribution can be derived from the knowledge that the image is selected from all possible images $I_m(u)$, weighted by their probabilities $p(I_m(u))$. Each image has a corresponding moment vector $\vec{x}(m)$. Therefore, the prior distribution $p(\vec{x})$ for each moment vector $\vec{x}$ can be calculated by weighting it according to the probabilities $p(I_m(u))$ of all the images $I_m(u)$ associated with the moments $\vec{x}$. For any moment vector $\vec{x}$ that does not correspond to a valid image $I_m(u)$, or if all corresponding images $I_m(u)$ have $p(I_m(u)) = 0$, we simply assume that $p(\vec{x}) = 0$. We define

$$d_k = \int d\vec{x} p(\vec{x}) x_k, \quad g_{k_1 k_2} = \int d\vec{x} p(\vec{x}) x_{k_1} x_{k_2}. \tag{26}$$

We can rewrite the eigenvalue problem as

$$G r_k = \lambda_k D r_k, \tag{27}$$

where $\lambda_k = (\beta_k^2 + 1)^{-1}$. Notice that $D_{kk} = \int d\vec{x} p(\vec{x}) P_k(\vec{x})$ and $G_{jk} = \int d\vec{x} p(\vec{x}) P_j(\vec{x}) P_k(\vec{x})$. We can expand D and G as a series of α

$$D = \sum_{n=0}^{\infty} D^{(n)} = \sum_{n=0}^{\infty} d_n \alpha^n \mathcal{C}_n, \quad \mathcal{C}_n = \begin{bmatrix} c_{0n} & 0 & 0 & \cdots \\ 0 & c_{1n} & 0 & \cdots \\ 0 & 0 & c_{2n} & \cdots \\ \cdots & \cdots & \cdots & \cdots \end{bmatrix}, \tag{28}$$

$$G = \sum_{n=0}^{\infty} G^{(n)} = \sum_{n=0}^{\infty} \alpha^n \sum_{i+j=n} g_{ij} C_i C_j^T, \quad C_i = [c_{0i}, c_{1i}, c_{2i}, \cdots]^T. \tag{29}$$

We can reformulate the eigenvector problem as $Wy_k = \lambda_k y_k$, where $W = D^{-1/2} G D^{-1/2}$. In the basis $\{z_n\}_{n=0,1,2,\cdots}$, which is the orthonormal basis constructed from the set of vectors $\{D^{-1/2} C_n\}_{n=0,1,2,\cdots}$ using the Gram-Schmidt procedure, the elements of W are given by $z_m^\dagger W z_n = \Theta(\alpha^{m+n})$. We can intuitively predict the scaling of eigenvalues from its character polynomial

$$|\lambda I - W| = \sum_{k=0}^{M} (-1)^k e_k \lambda^{M-k}, \tag{30}$$

where

$$e_0 = 1, \quad e_1 = \sum \lambda_i, \quad e_2 = \sum \lambda_i \lambda_j, \quad e_3 = \sum \lambda_i \lambda_j \lambda_k, \quad \cdots, \quad e_M = \prod_{i=1}^{M} \lambda_i. \tag{31}$$

To determine the scaling of λ_i, we only need to know the leading-order scaling of e_i with respect to α. We observe that selecting the largest k diagonal elements $z_n^\dagger W z_n$ provides the leading-order scaling for each e_k. This is because the contribution to each e_k from off-diagonal elements $z_m^\dagger W z_n$ can at most share the same order of scaling with respect to α. However, there is a possibility of cancellation among the contributions from the diagonal and off-diagonal elements, making this reasoning somewhat heuristic and not entirely rigorous. Essentially, we are trying to determine the scaling of e_k for a polynomial

$$(\lambda - \Theta(\alpha^0))(\lambda - \Theta(\alpha^2))(\lambda - \Theta(\alpha^4))(\lambda - \Theta(\alpha^6)) \cdots \tag{32}$$

We can read from it

$$e_1 = \Theta(\alpha^0), \quad e_2 = \Theta(\alpha^{0+2}), \quad e_3 = \Theta(\alpha^{0+2+4}), \quad \cdots \tag{33}$$

So, the scaling of eigenvalue

$$\lambda = \Theta(\alpha^0), \quad \Theta(\alpha^2), \quad \Theta(\alpha^4), \quad \Theta(\alpha^6), \quad \Theta(\alpha^8), \quad \cdots \tag{34}$$

which implies

$$\beta_0^2 = \Theta(1),\ \beta_1^2 = \Theta(\alpha^{-2}),\ \beta_2^2 = \Theta(\alpha^{-4}),\ \beta_3^2 = \Theta(\alpha^{-6}), \cdots \tag{35}$$

We now try to find the scaling of eigenvalues and the eigenbasis by perturbation theory up to the $O(\alpha^2)$ order for λ below,

$$\left(\sum_{n=0}^{\infty} G^{(n)}\right)\left(\sum_{n=0}^{\infty} r_k^{(n)}\right) = \left(\sum_{n=0}^{\infty} \lambda_k^{(n)}\right)\left(\sum_{n=0}^{\infty} D^{(n)}\right)\left(\sum_{n=0}^{\infty} r_k^{(n)}\right). \tag{36}$$

To the zeroth order

$$G^{(0)} r_k^{(0)} = \lambda_k^{(0)} D^{(0)} r_k^{(0)}, \tag{37}$$

$$\lambda_0^{(0)} = O(1), \quad \lambda_{j\neq 0}^{(0)} = 0, \quad r_0^{(0)} \propto [1, 1, 1, \cdots, 1]^T. \tag{38}$$

We easily prove that the properly normalized eigenvector $r_j^{(0)}$ satisfies

$$r_i^{(0)\dagger} D^{(0)} r_j^{(0)} = \delta_{ij}, \tag{39}$$

We now want to do degenerate perturbation theory in the subspace span$\{r_{k\neq 0}^{(0)}\}$.

$$\sum_{i+j=1} G^{(i)} r_k^{(j)} = \sum_{p+q+m=1} \lambda_k^{(p)} D^{(q)} r_k^{(m)}, \tag{40}$$

where $k \geq 1$. Notice that $\lambda_{k\geq 1}^{(0)} = 0$, $r.h.s = \lambda_k^{(1)} D^{(0)} r_k^{(0)}$. Notice that $G^{(0)}$ is a zero matrix in the subspace span$\{r_{j\neq 0}^{(0)}\}$, $l.h.s = G^{(1)} r_k^{(0)}$. Multiplying $r_l^{(0)\dagger}$ in both side

$$r_l^{(0)\dagger} G^{(1)} r_k^{(0)} = \lambda_k^{(1)} r_l^{(0)\dagger} D^{(0)} r_k^{(0)}, \tag{41}$$

where $k, l \geq 1$. Furthermore, notice that $r_{l\geq 1}^{(0)\dagger} D^{(0)} r_0^{(0)} \propto r_{l\geq 1}^{(0)\dagger} C_0^T = 0$ and $G^{(1)} = \alpha g_{01} C_0 C_1^T + \alpha g_{10} C_1 C_0^T$. It is clear that the $l.h.s$ of Eq. 41 vanishes. We thus have

$$\lambda_{k\geq 1}^{(1)} = 0. \tag{42}$$

Similarly, we can have the second order perturbation theory.

$$\sum_{i+j=2} G^{(i)} r_k^{(j)} = \sum_{p+q+m=2} \lambda_k^{(p)} D^{(q)} r_k^{(m)}. \tag{43}$$

Notice that $\lambda_{k\geq 1}^{(0,1)} = 0$, $r.h.s = \lambda_k^{(2)} D^{(0)} r_k^{(0)}$. Notice that $G^{(0,1)}$ is a zero matrix in the subspace span$\{r_{j\neq 0}^{(0)}\}$, $l.h.s = G^{(2)} r_k^{(0)}$. Multiplying $r_l^{(0)\dagger}$ in both side

$$r_l^{(0)\dagger} G^{(2)} r_k^{(0)} = \lambda_k^{(2)} r_l^{(0)\dagger} D^{(0)} r_k^{(0)} = \lambda_k^{(2)} \delta_{kl}, \tag{44}$$

$$r_l^{(0)\dagger} G^{(2)} r_k^{(0)} = \alpha^2 g_{11} r_l^{(0)\dagger} C_1 C_1^T r_k^{(0)}. \tag{45}$$

Since $r_l^{(0)\dagger} C_1 C_1^T r_k^{(0)}$ is rank 1, we have proved that $\lambda_1^{(2)} = \Theta(\alpha^2)$ and $\lambda_{j\geq 2}^{(2)} = 0$. Eigenvector $r_1^{(0)}$ is given by orthonogalizing $\mathcal{C}_0^{-1} C_1$ with respect to $r_0^{(0)} \propto \mathcal{C}_0^{-1} C_0$. Note this also means that z_n is the zeroth order eigenbasis for $W y_k = \lambda_k y_k$ for the first two eigenvectors because $\{z_n\}_{n=0,1,2,\cdots}$ is the orthogonalization of $\{D^{-1/2}\mathcal{C}_n\}_{n=0,1,2,\cdots}$. For higher order perturbation, we need to include the higher order correction of eigenbasis $r_k^{(j\geq 1)}$, which becomes more involved. Therefore, we choose to stop at this point.

B. Image a single compact source using SPADE method

As reviewed in Supplementary Note 1 A, the probability for SPADE method takes the form of

$$P_0 = \sum_{m=0}^{\infty} c_m x_m \alpha^m, \quad P_{n\pm} = \sum_{m\geq 2n} c_{nm}^{\pm} x_m \alpha^m, \quad n = 0, 1, 2, 3, \cdots \tag{46}$$

where the low order terms of $O(\alpha^{m\leq 2n-1})$ in $P_{n\pm}$ is cancelled out by construction. We can define

$$\begin{aligned} &G r_k = \lambda_k D r_k, \quad \lambda_k = (1+\beta_k^2)^{-1}, \\ &D = \sum_{n=0}^{\infty} D^{(n)} = \sum_{n=0}^{\infty} d_n \alpha^n \mathcal{C}_n, \quad \mathcal{C}_n = \Pi_{\lfloor n/2 \rfloor} \mathcal{C}_n^0 \Pi_{\lfloor n/2 \rfloor}, \\ &\mathcal{C}_n^0 = \begin{bmatrix} c_n & 0 & 0 & 0 & 0 & \cdots \\ 0 & c_{0n}^+ & 0 & 0 & 0 & \cdots \\ 0 & 0 & c_{0n}^- & 0 & 0 & \cdots \\ 0 & 0 & 0 & c_{1n}^+ & 0 & \cdots \\ 0 & 0 & 0 & 0 & c_{1n}^- & \cdots \\ \cdots & \cdots & \cdots & \cdots & \cdots & \cdots \end{bmatrix}, \end{aligned} \tag{47}$$

$$\begin{aligned} &G = \sum_{n=0}^{\infty} G^{(n)} = \sum_{n=0}^{\infty} \alpha^n \sum_{i+j=n} g_{ij} C_i C_j^T, \quad C_i = \Pi_{\lfloor i/2 \rfloor} C_i^0, \\ &C_i^0 = [c_i, c_{0i}^+, c_{0i}^-, c_{1i}^+, c_{1i}^-, \cdots]^T, \quad \Pi_m = \begin{bmatrix} I_{2m+3} & 0 \\ 0 & 0 \end{bmatrix}. \end{aligned} \tag{48}$$

In the following, we analyze the spectrum of the SPADE method using perturbation theory. To begin, we provide some intuition about the structure of the derived eigenbasis. Observing the form of G, we see that truncating its expansion at order $O(\alpha^n)$ makes the higher-order terms negligible, resulting in a rank-deficient matrix. For example, retaining terms only up to order α^2 gives

$$G = g_{00}C_0C_0^T + \alpha(g_{01}C_0C_1^T + g_{10}C_1C_0^T) + \alpha^2(g_{20}C_2C_0^T + g_{11}C_1C_1^T + g_{02}C_0C_2^T) + o(\alpha^2). \tag{49}$$

Such a truncated G captures the leading eigenvalues, as the neglected higher-order terms contribute only weakly to the spectrum. A key difference from the direct imaging case is that the structure $C_i = \Pi_{\lfloor i/2 \rfloor} C_i^0$ forces certain components to vanish, effectively restricting the support of each C_i. When we examine the eigenvectors within the subspace spanned by C_0, C_1, C_2, we find that the matrix r_{nm} takes an upper-triangular form. The higher-order eigentasks are dominated by higher-order moments in the case of imaging a single compact source. This intuition is rigorously validated by the perturbative analysis presented below.

We again reformulate the eigenvalue problem as $Wy_k = \lambda_k y_k$, $W = D^{-1/2}GD^{-1/2}$. Note the lower order D is not full rank. Rewriting the problem as standard eigenvalue problem avoids this difficulty. To further simplify the calculation, we will divide the matrix into blocks, the (m,n)th block is given by

$$\begin{aligned} W_{mn} =& [D^{-1/2}GD^{-1/2}]_{mn} \\ =& P_m \left(\frac{\alpha^{-m}}{\sqrt{d_{2m}}} \mathcal{C}_{2m}^{-1/2} \left[I - \frac{\alpha}{2}\frac{d_{2m+1}}{d_{2m}} \mathcal{C}_{2m+1}\mathcal{C}_{2m}^{-1} + \frac{3}{8}\alpha^2 \frac{d_{2m+1}^2}{d_{2m}^2} \mathcal{C}_{2m+1}^2 \mathcal{C}_{2m}^{-2} - \frac{1}{2}\alpha^2 \frac{d_{2m+2}}{d_{2m}} \mathcal{C}_{2m+2}\mathcal{C}_{2m}^{-1} + \cdots \right] \right) \\ & \times \sum_{i\geq 2m, j\geq 2n} \alpha^{i+j} g_{ij} C_i C_j^T \\ & \times \left(\frac{\alpha^{-n}}{\sqrt{d_{2n}}} \mathcal{C}_{2n}^{-1/2} \left[I - \frac{\alpha}{2}\frac{d_{2n+1}}{d_{2n}} \mathcal{C}_{2n+1}\mathcal{C}_{2n}^{-1} + \frac{3}{8}\alpha^2 \frac{d_{2n+1}^2}{d_{2n}^2} \mathcal{C}_{2n+1}^2 \mathcal{C}_{2n}^{-2} - \frac{1}{2}\alpha^2 \frac{d_{2n+2}}{d_{2n}} \mathcal{C}_{2n+2}\mathcal{C}_{2n}^{-1} + \cdots \right] \right) P_n, \end{aligned} \tag{50}$$

where $P_n = \Pi_n - \Pi_{n-1}$, $\Pi_{m<0} = 0$. Note the leading order of block W_{mn} has lowest order $\Theta(\alpha^{m+n})$.

We will define the following set of orthonormal basis for convenience

$$z_{2k} \propto P_k \mathcal{C}_{2k}^{1/2} t, \quad z_{2k+1} \perp z_{2k}, \quad z_{2k+1} \in P_k, \tag{51}$$

where $k = 0, 1, 2, 3, \cdots$, and z_{2k+1} is the vector constructed by orthogonalize $P_k \mathcal{C}_{2k}^{-1/2} \mathcal{C}_{2k+1} t$ over z_{2k}. And it should be clear that in this basis, for the (m,n)th block $W_{mn} = P_m W P_n$, the scaling of over α is given by

$$\begin{bmatrix} \Theta(\alpha^{m+n}) & \Theta(\alpha^{m+n+1}) \\ \Theta(\alpha^{m+n+1}) & \Theta(\alpha^{m+n+2}) \end{bmatrix}, \tag{52}$$

To find the scaling of eigenvalues, we again try to observe the structure of the character polynomial $|\lambda I - W| = \sum_{k=0}^{M} (-1)^k e_k \lambda^{M-k}$, its coefficients now has the form of

$$\begin{aligned} &e_1 = \Theta(\alpha^0), \quad e_2 = \Theta(\alpha^{0+2}), \quad e_3 = \Theta(\alpha^{0+2+2}), \\ &e_4 = \Theta(\alpha^{0+2+2+4}), \quad e_5 = \Theta(\alpha^{0+2+2+4+4}), \cdots \end{aligned} \tag{53}$$

So, the scaling of eigenvalue is

$$\beta_0^2 = \Theta(1), \beta_1^2 = \Theta(\alpha^{-2}), \beta_2^2 = \Theta(\alpha^{-2}), \beta_3^2 = \Theta(\alpha^{-4}), \beta_4^2 = \Theta(\alpha^{-4}), \cdots \tag{54}$$

We now calculate the eigenvalues and eigenbasis based on perturbation theory. We can write W as a series of α,

$$W = \sum_{n=0}^{\infty} W^{(n)} \alpha^n. \tag{55}$$

The zeroth order term

$$W^{(0)} \propto P_0 \mathcal{C}_0^{1/2} t t^T \mathcal{C}_0^{1/2} P_0, \tag{56}$$

where $t = [1, 1, 1, \cdots]^T$. Using perturbation theory,

$$W^{(0)} y_k^{(0)} = \lambda_k^{(0)} y_k^{(0)}, \tag{57}$$

it is clear that

$$\lambda_0 = \Theta(1), \quad y_0^{(0)} \propto P_0 \mathcal{C}_0^{1/2} t \propto z_0. \tag{58}$$

Since we have $r_i = D^{-1/2} y_i$, we find that

$$r_0^{(0)} \propto P_0 t. \tag{59}$$

Obviously, we have

$$\lambda_{i\neq 0}^{(0)} = 0. \tag{60}$$

Furthermore, since $W^{(0)}$ is rank one, we will find the proper first order approximation to eigenvector $y_{i\geq 1}^{(0)}$ as a linear combination of $z_{i\geq 1}$ in the higher order perturbation discussion.

With this choice of z_k, we can continue the calculation for $\Theta(\alpha)$ terms.

$$W^{(1)} y_k^{(0)} + W^{(0)} y_k^{(1)} = \lambda_k^{(1)} y_k^{(0)}, \quad k \geq 1. \tag{61}$$

If we apply $(z_{j\geq 1})^T$ from the left and insert $y_k^{(0)} = \sum_{m=1}^{\infty} a_{km}^{(0)} z_m$, we get

$$\sum_{m=1}^{\infty} \left((z_j)^T W^{(1)} z_m - \delta_{mj} \lambda_k^{(1)} \right) a_{km}^{(0)} = 0, \tag{62}$$

where we have used the fact that $W^{(0)} z_{i\geq 1} = 0$, which is because of our construction in Eq. 51 and $W^{(0)} \sim P_0 \mathcal{C}_0^{1/2} t t^T \mathcal{C}_0^{1/2} P_0$. Since $W^{(1)}$ has terms proportional to

$$P_0 \mathcal{C}_0^{1/2} t t^T \mathcal{C}_1 \mathcal{C}_0^{-1/2} P_0, \quad P_0 \mathcal{C}_0^{-1/2} \mathcal{C}_1 t t^T \mathcal{C}_0^{1/2} P_0, \quad P_0 \mathcal{C}_0^{1/2} t t^T \mathcal{C}_2 \mathcal{C}_0^{-1/2} P_1, \quad P_1 \mathcal{C}_0^{-1/2} \mathcal{C}_2 t t^T \mathcal{C}_0^{1/2} P_0, \tag{63}$$

and $z_{k\geq 1}$ to orthogonal to $y_0^{(0)} \propto P_0 \mathcal{C}_0^{1/2} t$, we have $(z_j)^T W^{(1)} z_m = 0$ for $j, m \geq 1$.

$$\lambda_k^{(1)} = 0 \quad \text{for} \quad k \geq 1. \tag{64}$$

We then consider the $\Theta(\alpha^2)$ terms

$$W^{(2)} y_k^{(0)} + W^{(1)} y_k^{(1)} + W^{(0)} y_k^{(2)} = \lambda_k^{(2)} y_k^{(0)}, \quad k \geq 1. \tag{65}$$

Apply $z_{j\geq 1}$ from left,

$$\sum_{m=1}^{\infty} \left((z_j)^T W^{(2)} z_m - \delta_{mj} \lambda_k^{(2)} \right) a_{km}^{(0)} = 0, \tag{66}$$

where we have used the fact that $z_{i\geq 1} W^{(0)} z_{j\geq 1} = z_{i\geq 1} W^{(1)} z_{j\geq 1} = 0$ as argued above. Since $W^{(2)}$ has terms proportional to

$$P_0 \mathcal{C}_1 \mathcal{C}_0^{-1/2} t t^T \mathcal{C}_1 \mathcal{C}_0^{-1/2} P_0, \quad P_1 \mathcal{C}_2^{1/2} t t^T \mathcal{C}_2^{1/2} P_1, \quad P_0 \mathcal{C}_1 \mathcal{C}_0^{-1/2} t t^T \mathcal{C}_2^{1/2} P_1, \quad P_0 \mathcal{C}_1 \mathcal{C}_0^{-1/2} t t^T \mathcal{C}_2^{1/2} P_1, \tag{67}$$

This shows

$$\lambda_{1,2}^{(2)} \neq 0, \quad \lambda_{k\geq 3}^{(2)} = 0, \tag{68}$$

and also provides the leading order approximation to $y_{1,2}^{(0)}$ as a linear combination of $z_{1,2}$. By the orthogonality condition, we must have $y_{i\geq 3}^{(0)} \perp z_{j\leq 2}$.

We can then consider the $\Theta(\alpha^3)$ terms

$$W^{(3)} y_k^{(0)} + W^{(2)} y_k^{(1)} + W^{(1)} y_k^{(2)} + W^{(0)} y_k^{(3)} = \lambda_k^{(3)} y_k^{(0)}, \quad k \geq 3. \tag{69}$$

We apply $(z_{j\geq 3})^T$ from the left

$$\sum_{m=3}^{\infty} a_{km}^{(0)} (z_j)^T W^{(3)} z_m + \sum_{m=1}^{\infty} a_{km}^{(1)} (z_j)^T W^{(2)} z_m = \lambda_k^{(3)} a_{kj}^{(0)}. \tag{70}$$

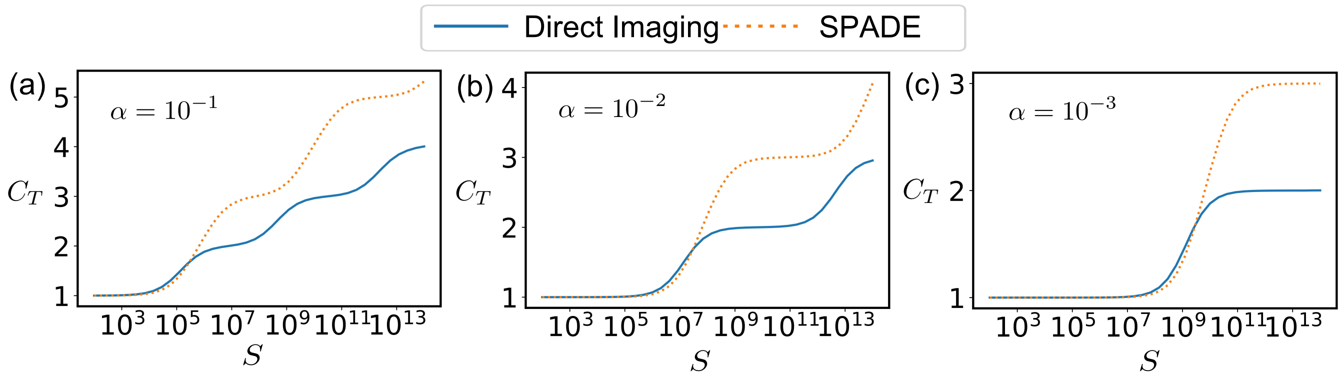

Supplementary Figure 2. Total REC C_T for direct imaging and the SPADE method as a function of S, when imaging one generally distributed compact source with different α.

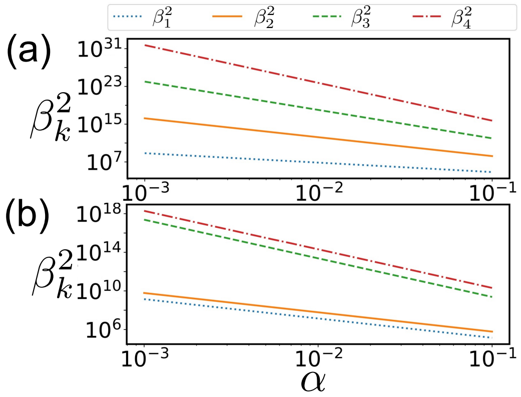

Supplementary Figure 3. Scaling of the β_k^2 as a function of α for (a) direct imaging and (b) SPADE method, when imaging a generally distributed compact source. $\beta_0^2 = 0$ is omitted from the plot. Note that $\beta_{3,4}^2$ in 2(a) is interpolated using values calculated for cases where $\alpha > 10^{-1}$ that are not explicitly shown in the plot.

Analyzing each terms of $W^{(2)}$ and $W^{(3)}$, we conclude the left hand side vanishes and this shows

$$\lambda_{k\geq 3}^{(3)} = 0. \tag{71}$$

We demonstrate that β_k^2 acts as the threshold for the stepwise increase in the total REC, C_T, as illustrated in Supplementary Figure 2 for different values of α separately. For direct imaging, C_T increases by 1 at each step, while for the SPADE method, C_T increases by 2 per step, as expected. Moreover, as α decreases, the plateau regions become more pronounced.

C. Numerical results for eigenvalues β_k^2

In the previous subsections, we provided a rigorous analytical proof based on perturbation theory that applies to any prior distribution $p(\vec{x})$ and PSF. For direct imaging, we showed that $\beta_0^2 = 0$, $\beta_1^2 = \Theta(\alpha^{-2})$, and $\beta_{k\geq 2}^2 = \omega(\alpha^{-2})$. More generally, we expect the scaling $\beta_k^2 = \Theta(\alpha^{-2k})$ to hold for higher-index eigenvalues under any prior. For the superresolution case, we demonstrated that $\beta_0^2 = 0$, $\beta_1^2 = \Theta(\alpha^{-2})$, $\beta_2^2 = \Theta(\alpha^{-2})$, and $\beta_{k\geq 3}^2 = \omega(\alpha^{-2})$, and we conjecture that the scaling $\beta_k^2 = \Theta(\alpha^{-2\lceil k/2 \rceil})$ persists for larger k.

The scalings of the first five eigenvalues for the direct imaging case are confirmed by numerical calculations, as shown in Supplementary Figure 3 (a). In the superresolution case, Supplementary Figure 3 (b) shows that the first five eigenvalues, computed numerically for a random prior, form pairs $\{\beta_{2k-1}^2, \beta_{2k}^2\}_{k\geq 1}$ with identical scaling starting from $k = 1$, also consistent with our analytical proof. Values of β_k^2 exceeding $\sim 10^{15}$ in Supplementary Figure 3 are

interpolated using a straight-line fit at larger α to mitigate numerical precision loss; this interpolation is applied to all β_k^2–α plots.

All numerical calculations in this work assume a Gaussian PSF, $\psi(u) = \exp\left(-u^2/4\sigma^2\right)/(2\pi\sigma^2)^{1/4}$, although our method is applicable to arbitrary PSFs. The prior distribution $p(\vec{x})$ is constructed by randomly generating a set of images and assigning them equal probability, so that $p(\vec{x})$ corresponds to the empirical distribution of the resulting moment vectors. Further details are provided in Supplementary Note 6. For illustration, we show results for a single instance of the randomly generated prior (as is the case for all figures below that involve a randomly chosen prior).

Note that the above analysis is limited to the first few eigenvalues β_k^2, although we expect the same scaling behavior to extend to higher indices k. To avoid potential caveats and ensure clarity, Table Supplementary Table II summarizes the scaling behavior that has been explicitly calculated and confirmed through both analytical and numerical methods.

**Imaging Type**	**Eigenvalue Index k**	**Approach**	**PSF Used**	**Prior Used**
**Analytical Results**				
Direct Imaging	0	0	Arbitrary	Arbitrary
	1	$\Theta(\alpha^{-2})$	Arbitrary	Arbitrary
	2	$\omega(\alpha^{-2})$	Arbitrary	Arbitrary
	3	$\omega(\alpha^{-2})$	Arbitrary	Arbitrary
Superresolution	0	0	Arbitrary	Arbitrary
	1	$\Theta(\alpha^{-2})$	Arbitrary	Arbitrary
	2	$\Theta(\alpha^{-2})$	Arbitrary	Arbitrary
	3	$\omega(\alpha^{-2})$	Arbitrary	Arbitrary
	4	$\omega(\alpha^{-2})$	Arbitrary	Arbitrary
**Numerical Results**				
Direct Imaging	0	0	Gaussian	Random empirical
	1	$\Theta(\alpha^{-2})$	Gaussian	Random empirical
	2	$\Theta(\alpha^{-4})$	Gaussian	Random empirical
	3	$\Theta(\alpha^{-6})$	Gaussian	Random empirical
	4	$\Theta(\alpha^{-8})$	Gaussian	Random empirical
Superresolution	0	0	Gaussian	Random empirical
	1	$\Theta(\alpha^{-2})$	Gaussian	Random empirical
	2	$\Theta(\alpha^{-2})$	Gaussian	Random empirical
	3	$\Theta(\alpha^{-4})$	Gaussian	Random empirical
	4	$\Theta(\alpha^{-4})$	Gaussian	Random empirical

Supplementary Table II. Summary of the asymptotic scaling behavior of eigenvalues β_k^2 from both analytical and numerical approaches. Analytical results apply to arbitrary PSFs and priors, while numerical results are obtained for a Gaussian PSF and a randomly generated empirical prior.

D. Deviation of the approximated eigenbasis from the actual eigenbasis

In this section, we want to verify the deviation of the approximated eigenbasis $\hat{y}_k$ from the actual eigenbasis y_k vanishes as $\alpha \to 0$. In the direct imaging case, we predicted above that $\{z_n\}_{n=0,1,2,\cdots}$, which is the orthonormal basis constructed from the set of vectors $\{D^{-1/2}C_n\}_{n=0,1,2,\cdots}$ using the Gram-Schmidt procedure, are the approximated eigenvector $\hat{y}_k = z_k$ for actual eigenvectors y_k, which is confirmed as shown in Supplementary Figure 4(a1). For the SPADE method, we predicted above that the approximated eigenvector $\hat{y}_0 = z_0$, each $\hat{y}_{k\geq 1}$ is the linear combination of $z_{2\lceil k/2\rceil-1}$ and $z_{2\lceil k/2\rceil}$ in Eq. 51. The coefficients in the linear combination of the two bases depend on the specific values of g_{ij} and d_i. We choose $\hat{y}_0 = z_0$ and employ a least squares approach to fit each $\hat{y}_{k\geq 1}$ with the two bases $z_{2\lceil k/2\rceil-1}$ and $z_{2\lceil k/2\rceil}$ in Eq. 51. The deviation is illustrated in Supplementary Figure 4(b1). In both cases, as $\alpha \to 0$, the actual eigenbasis y_k increasingly aligns with the eigenvectors $\hat{y}_k$ described above. For the numerical calculations, we select the PSF as $\psi(u) = \exp\left(-u^2/4\sigma^2\right)/(2\pi\sigma^2)^{1/4}$. The coefficients d and g are chosen by randomly generating a set of images and assuming that these images appear with equal probability, which then can be used to d and g with the corresponding moments vector of each image as detailed in Supplementary Note 6.

We also plot the actual eigenbasis y_k and r_k in Supplementary Figure 4(a2)(a3) for the direct imaging method

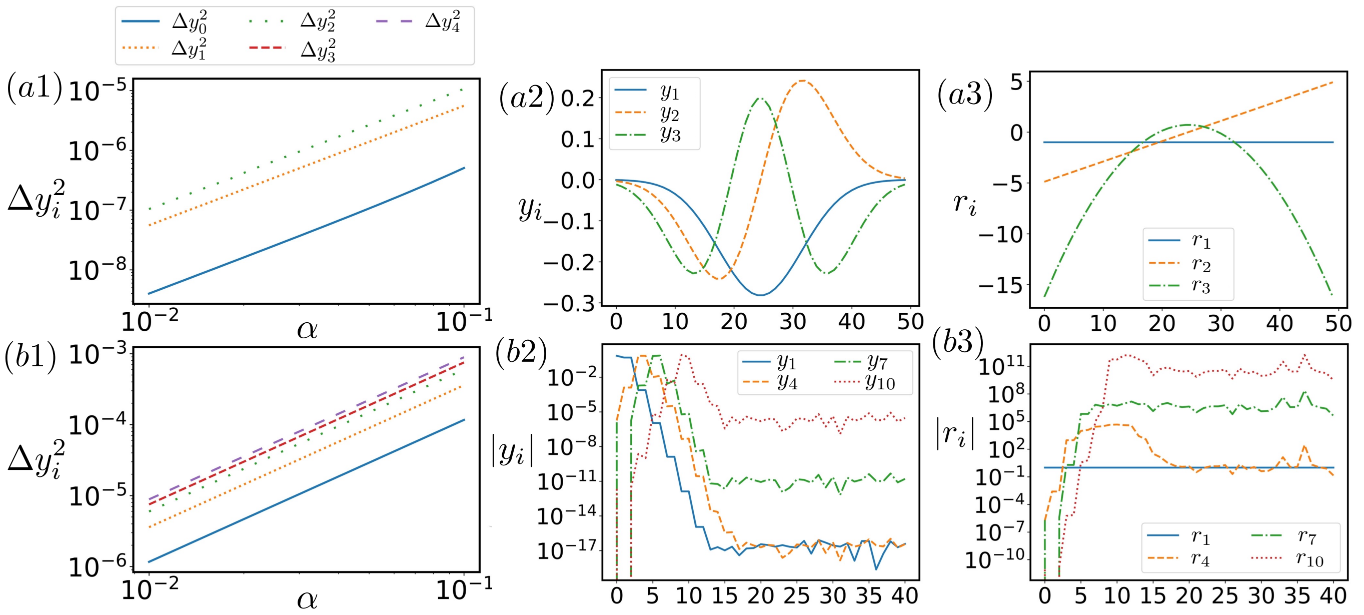

Supplementary Figure 4. We plot the deviation of the approximated eigenvector $\hat{y}_k$ from the actual eigenbasis y_k for imaging a single compact source using (a1) direct imaging and (b1) the SPADE method. The deviation is defined as the squared norm of the difference between two normalized vectors, $\Delta y_k^2 = |\hat{y}_k - y_k|^2$, for the eigenvalue problem $W y_k = \lambda_k y_k$, where $W = D^{-1/2} G D^{-1/2}$. We also show the actual eigenbasis y_k in (a2) for direct imaging and (b2) for the SPADE method. In (b2), we plot the absolute value to display it on a logarithmic scale. Additionally, we present the vectors r_k in (a3) for direct imaging and (b3) for the SPADE method. The x-axis in (a2), (b2), (a3), (b3) represents the different measurement outcomes, and we set $\alpha = 10^{-2}$ for these figures.

and in Supplementary Figure 4(b2)(b3) for the SPADE method. The x-axis corresponds to different measurement outcomes. For direct imaging [Supplementary Figure 4(a2)(a3)], the measurement outcome corresponds to detecting a single photon at each pixel on the image plane. For the SPADE method [Supplementary Figure 4(b2)(b3)], we plot the absolute value $|y_k|, |r_k|$, with the outcomes labeled as $P_0, P_{1+}, P_{1-}, P_{2+}, P_{2-}, \ldots$. It is evident that $|y_k|$ is concentrated on just a few outcomes and $|r_k|$ is almost vanishing for the first few components. Since for the SPADE method, $P_{n\pm} = c^{\pm}_{n,2n} x_{2n} \alpha^{2n} + c^{\pm}_{n,2n+1} x_{2n+1} \alpha^{2n+1} + o(\alpha^{2n+1})$, it follows that the eigentasks are dominated by only a few moments. Note that in the figure we plot y_k, the eigenvector of $D^{-1/2} G D^{-1/2}$ defined by $D^{-1/2} G D^{-1/2} y_k = \lambda_k y_k$, which is related to r_k by $y_k = D^{1/2} r_k$. We use y_k because it is normalized without the weight matrix, i.e., $y_k^\dagger y_k = 1$, whereas $r_k^\dagger D r_k = 1$. Using y_k makes it easier to see which components become dominant.

Supplementary Note 4. IMAGING GENERAL SOURCE EXTENDED OUTSIDE THE RAYLEIGH LIMIT

In the above discussion, we have consistently assumed the size of one compact source is much smaller than the Rayleigh limit of our lens. However, in practice, the source size can exceed the Rayleigh limit, and we still need to quantify imaging performance in such cases. In this section, we focus on quantifying imaging performance using the REC under these conditions.

A natural question is what the total REC would be for a general imaging task using direct imaging and how to interpret its meaning. Intuitively, when imaging a source of size L with a lens where the PSF has a width σ, the resulting image is a blurred version of the original, with the blur size being σ. The total REC represents the number of degrees of freedom we can extract from the measurement, and since the image is blurred, this is roughly of order L/σ; however, this relation is not exact, as it arises from heuristic reasoning rather than formal analysis. To confirm this, we perform a numerical calculation as follows: we randomly generate a general source of size L consisting of $N_{\max}$ discrete pixels,with each pixel assigned a random value between 0 and 1. We then normalize the intensity distribution $I(u)$ such that $\sum_u I(u) = 1$. By repeatedly generating the intensity distribution $I(u)$ in this manner for W times, we simulate the case where we do not have any prior knowledge of the source. For all the numerical calculation for imaging general source outside the Rayleigh limit, we use $N_{\max} = 200$, $W = 200$. Assuming the PSF is given by

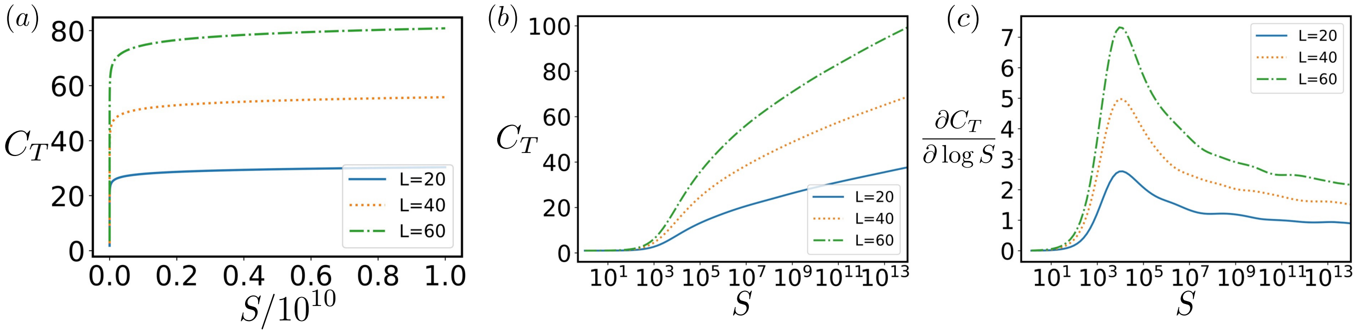

Supplementary Figure 5. (a) Total REC for direct imaging as a function of S for a general source with size $L = 20, 40, 60$ respectively and width of PSF $\sigma = 1$. (b) Total REC C_T for direct imaging of general source extended outside the Rayleigh limit as a function of S in log scale for source with size $L = 20, 40, 60$. (c) Slope $(\partial C_T)/(\partial \log S)$ as a function of S, for source with size $L = 20, 40, 60$. $\sigma = 1$.

$\psi(u) = (2\pi\sigma^2)^{-1/4} \exp\big(-u^2/(4\sigma^2)\big)$.

We plot the total REC as a function of sample number S for different values of L, as shown in Supplementary Figure 5(a). As expected, the total C_T soon reaches a (slowly increasing) plateau as $S \to \infty$. We also present in Supplementary Figure 5(b) a plot of C_T as a function of S on a logarithmic scale. As discussed in the main text, when moving beyond the regime of resolving multiple compact sources—where the sources are no longer confined to a few localized regions—the increase of C_T no longer exhibits stepwise behavior. Note that as $S \to \infty$, the total REC C_T continues to increase slowly and eventually diverges. This implies that, with infinitely many photons, even direct imaging can resolve the image to an arbitrarily fine degree as a number of investigations over the last fifty years have showed [34–36]. However, to reach this infinite C_T, the required number of samples also diverges. In particular, when S becomes sufficiently large, the increase of C_T becomes very slow. This behavior is illustrated in Supplementary Figure 5(c), which shows that the slope $(\partial C_T)/(\partial \log S)$ reaches a peak at some sample size and then gradually decreases. In this sense, C_T exhibits a logarithmic-type growth with respect to S, characterized by a relatively small coefficient when S becomes sufficiently large. Since $\log S$ is used as the x-axis in the slope plot, advancing by a fixed interval along the $\log S$ axis requires an exponentially larger increase in the number of samples. Meanwhile, C_T increases only modestly over the same interval of $\log S$. Therefore, the curve approaches a slowly rising plateau.

We also demonstrate that the total REC C_T increases approximately linearly with the source size L, as shown in Supplementary Figure 6(a) for different choices of σ. The ratio $C_T/(L/\sigma)$ is plotted in Supplementary Figure 6(b), which approaches 1 but shows a slight decreasing trend as L increases in this model across various values of σ. In Supplementary Figure 7, we plot the total REC C_T as a function of the PSF width σ for different source sizes L. Our numerical results indicate that, for the model considered here, C_T approximately follows a power-law scaling: $C_T \propto \sigma^{-0.9}$. We have thus observed that the total REC C_T is approximately determined by the ratio L/σ, but this relation is not exact—rather, it provides a rough estimate that serves only as an intuitive argument rather than a rigorous treatment. All the above calculations are performed for a single instance of randomly generated prior information. However, in Supplementary Figure 8, we present results from three consecutive executions of the calculation with $N_{\max} = 200$ and $W = 200$, where each execution randomly generates a different instance of prior information. Despite the variation in prior information, we observe negligible differences in the calculation results.

Supplementary Note 5. IMAGING MULTIPLE COMPACT SOURCES

The state from multiple compact sources is given by

$$\rho = \sum_{q=1}^{Q} \int du du_1 du_2 I_q(u) \psi(u - u_1) |u_1\rangle \langle u_2| \psi^*(u - u_2), \tag{72}$$

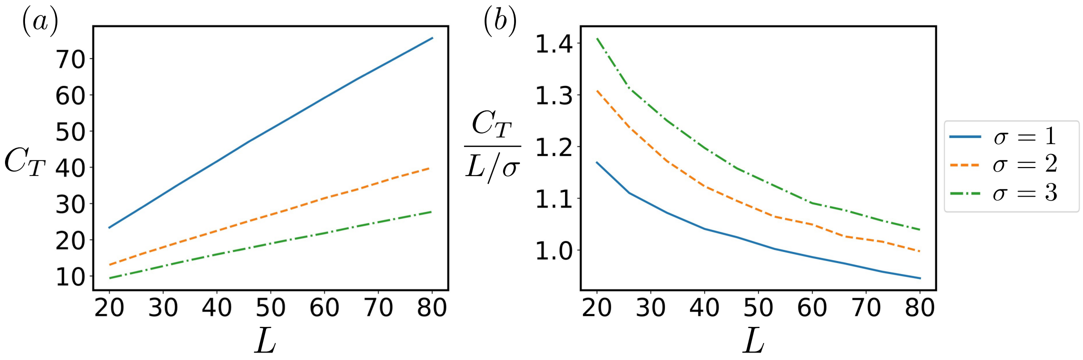

Supplementary Figure 6. (a) Total REC C_T for direct imaging of a general source that extends outside the Rayleigh limit, plotted as a function of the source size L; (b) the ratio $C_T/(L/\sigma)$ as a function of L, for different values of the PSF width $\sigma = 1, 2, 3$. In both panels, the total sample number is fixed at $S = 10^7$.

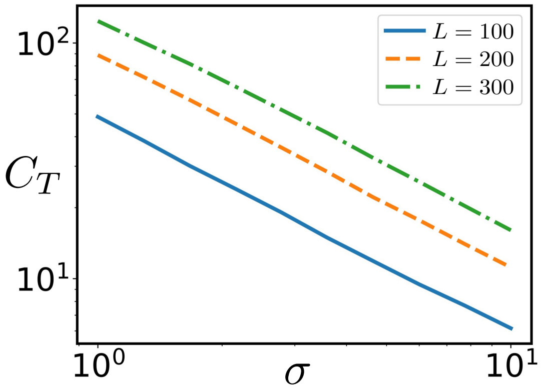

Supplementary Figure 7. Total REC C_T for direct imaging of a general source extending beyond the Rayleigh limit, plotted as a function of the PSF width σ for different source sizes $L = 100, 200, 300$. Both axes use logarithmic scales. The sample number is $S = 10^4$.

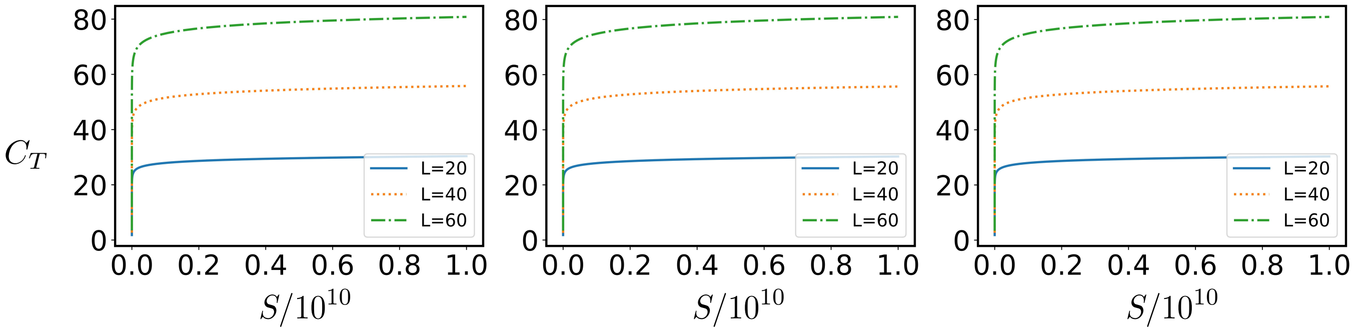

Supplementary Figure 8. Three consecutive executions of the calculation of total REC C_T for direct imaging of general source extended outside the Rayleigh limit as a function of S for source with size $L = 20, 40, 60$. $\sigma = 1$.

where Q is the number of compact sources, $I_q(u)$ is the intensity distribution for qth compact source. We can expand the PSF near the centroid u_q of qth source

$$\begin{aligned}\psi(u_1-u) &= \sum_{n=0}^{\infty}\left(\frac{\partial^n\psi(u_1-u)}{\partial u^n}\bigg|_{u=u_q}\frac{L_q^n}{n!}\right)\left(\frac{u-u_q}{L_q}\right)^n \\ &= \sum_{n=0}^{\infty}\psi_q^{(n)}(u_1)\left(\frac{u-u_q}{L_q}\right)^n,\end{aligned} \tag{73}$$

where L_q is the size of qth source.

$$\begin{aligned}\rho &= \sum_{q=1}^{Q}\sum_{m,n=0}^{\infty}x_{m+n,q}\left|\psi_q^{(m)}\right\rangle\left\langle\psi_q^{(n)}\right|, \\ \left|\psi_q^{(m)}\right\rangle &= \int du\psi_q^{(m)}(u)\left|u\right\rangle,\end{aligned} \tag{74}$$

where $x_{n,q}=\int duI_q(u)\left(\frac{u-u_q}{L_q}\right)^n$ is the nth moment for the qth source.

A. Direct imaging

If we have Q compact sources, direct imaging will have the probability distribution in the following form

$$P_n=\sum_{q=1}^{Q}\sum_{m=0}^{\infty}c_{nmq}x_{mq}\alpha_q^m. \tag{75}$$

Again, we measure a discretized version of direct imaging for both analytical and numerical discussions $M_n=\int_{X_n-l/2}^{X_n+l/2}du\left|u\right\rangle\left\langle u\right|$. Notice that $\alpha_q=L_q/\sigma$ can be different. But in the following, we will redefine the coefficients c_{nmq} involves the difference between L_q for different sources and have $\alpha_q=\alpha$. We will numerically explore the case where α_q differs significantly and cannot be incorporated into the coefficients c_{nmq} below.

$$\begin{aligned}&D=\sum_{n=0}^{\infty}D^{(n)}=\sum_{n=0}^{\infty}\alpha^n\sum_{q=1}^{Q}d_{nq}\mathcal{C}_{nq},\quad \mathcal{C}_{nq}=\mathrm{diag}(C_{nq}),\quad C_{nq}=[c_{0nq},c_{1nq},c_{2nq},\cdots\cdots]^T,\\ &G=\sum_{n=0}^{\infty}G^{(n)}=\sum_{n=0}^{\infty}\alpha^n\sum_{n_1+n_2=n}\sum_{q_1,q_2=1}^{Q}g_{n_1q_1,n_2q_2}C_{n_1q_1}C_{n_2q_2}^T,\\ &g_{n_1q_1,n_2q_2}=\int d\vec{x}p(\vec{x})x_{n_1q_1}x_{n_2q_2},\quad d_{nq}=\int d\vec{x}p(\vec{x})x_{nq}.\end{aligned} \tag{76}$$

where $\mathrm{diag}(C_{nq})$ denotes a diagonal matrix with the diagonal elements being the elements of C_{nq}. If we choose a set of basis z_n as the Gram-Schmidt orthogonalization of $D_0^{-1/2}C_{mq}$, we can then express the matrix $W=D^{-1/2}GD^{-1/2}$ in the basis of z_n and divide it into $Q\times Q$ blocks. In this block structure, the (m,n)th block scales as $\Theta(\alpha^{m+n})$. We can then examine the structure of the characteristic polynomial $|\lambda I-W|=\sum_{k=0}^{M}(-1)^k e_k\lambda^{M-k}$, where the coefficients e_k now take the form of

$$e_1=\Theta(\alpha^0),\cdots,e_Q=\Theta(\alpha^0),e_{Q+1}=\Theta(\alpha^{0+2}),\cdots,e_{2Q}=\Theta(\alpha^{0+2}),e_{2Q+1}=\Theta(\alpha^{0+2+4}),\cdots,e_{3Q}=\Theta(\alpha^{0+2+4}),\cdots\cdots \tag{77}$$

So, the scaling of eigenvalue is

$$\beta^2_{0\le i\le Q-1}=\Theta(1),\quad \beta^2_{Q\le i\le 2Q-1}=\Theta(\alpha^{-2}),\quad \beta^2_{2Q\le i\le 3Q-1}=\Theta(\alpha^{-4}),\cdots \tag{78}$$

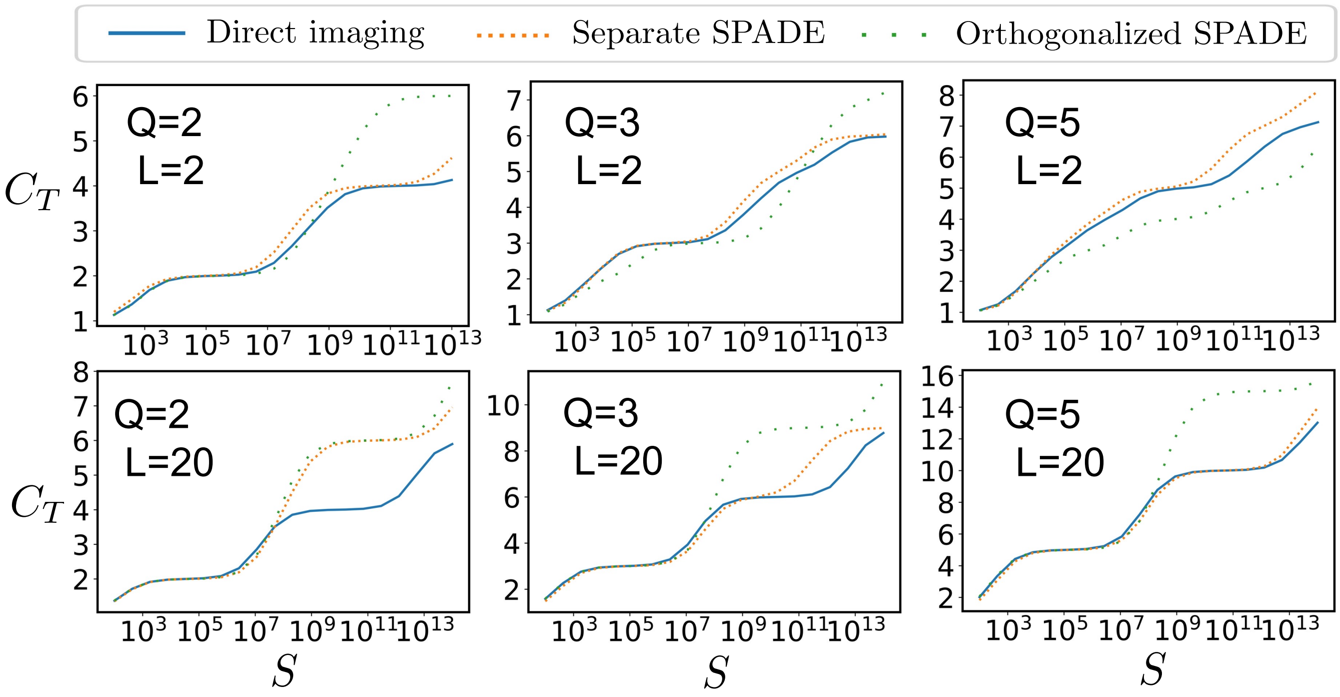

Supplementary Figure 9. Total REC C_T of imaging Q compact sources for direct imaging, separate SPADE method and orthogonalized SPADE method as a function of S. $\alpha = 10^{-2}$, $\sigma = 1$. We consider two different L and different number of compact source Q.

B. Separate SPADE method

If we naively apply the SPADE method [4] to each compact source, which will be referred as the separate SPADE method. In other word, we use the POVM

$$\left\{ \frac{1}{2Q} \left| \phi_{q,m,\pm} \right\rangle \left\langle \phi_{q,m,\pm} \right|, \frac{1}{2Q} \left| b_{q,0} \right\rangle \left\langle b_{q,0} \right| \right\}_{q=1,2,\cdots,Q,\, m=0,1,2,\cdots,\infty}, \tag{79}$$

where $\{|b_{q,m}\rangle\}_{m=0,1,2,\cdots,\infty}$ is given by the Gram-Schmidt orthogonalization of set $\{\left|\psi_q^{(m)}\right\rangle\}_{m=0,1,2,\cdots,\infty}$, and $|\phi_{q,m,\pm}\rangle = \frac{1}{\sqrt{2}}(|b_{q,m}\rangle \pm |b_{q,m+1}\rangle)$. We add the normalization factor $1/Q$ to our POVM.

An immediate issue arises: nearby compact sources can disrupt the construction of separate SPADE method. This occurs because the designed measurement relies on canceling out lower-order terms of $O(\alpha^n)$ through careful design. However, if other compact sources are too close, they may contribute terms that are much larger than $O(\alpha^n)$, undermining the effectiveness of the superresolution technique.

In general, when all compact sources are sufficiently far apart, superresolution can still be achieved. However, when the sources are too close together, the probability distribution of separate SPADE method will resemble that of direct imaging, as described in Eq. 75. To be more specific, if we choose the PSF as $\psi(u) = \exp\left(-u^2/4\sigma^2\right)/(2\pi\sigma^2)^{1/4}$, the threshold for achieving superresolution is determined by whether $\alpha^n \gg \exp\left(-L^2/\sigma^2\right)$, where n is determined by the order of the measured moments, and $\exp\left(-L^2/\sigma^2\right)$ represents the contribution from nearby compact sources.

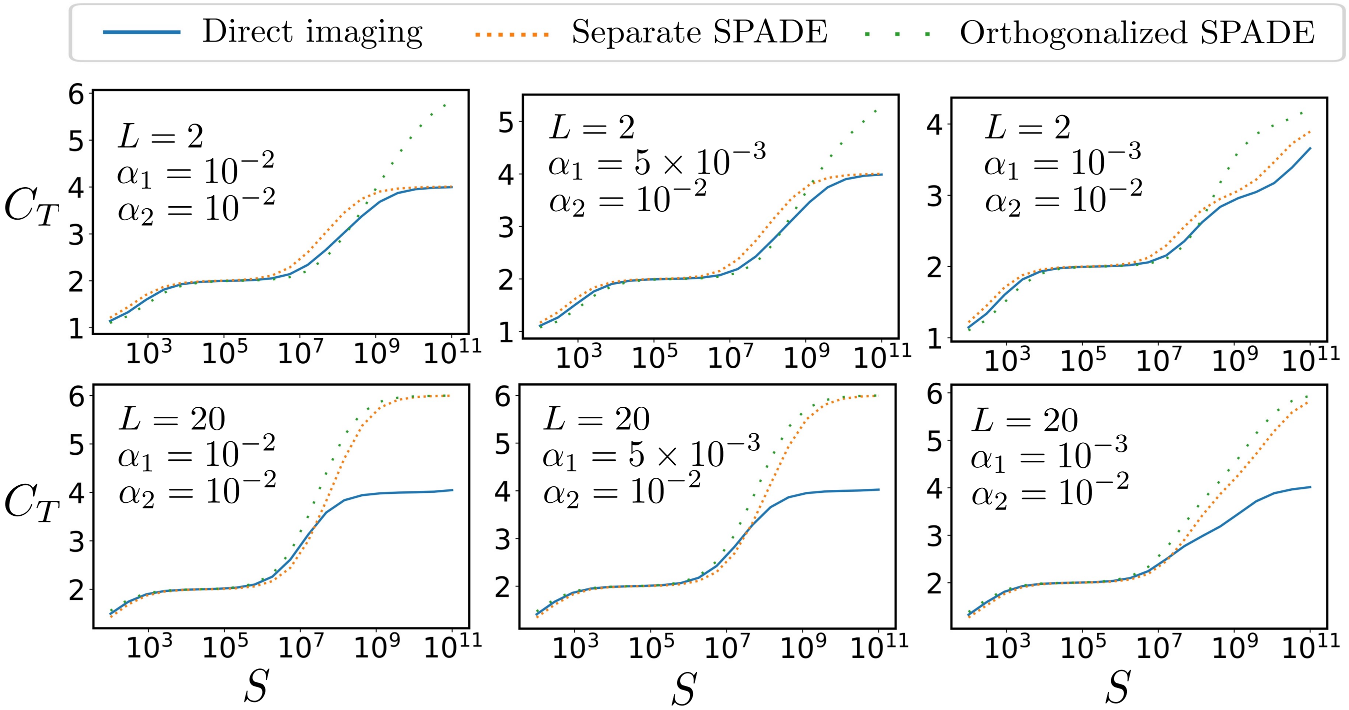

Supplementary Figure 10. Total REC C_T for direct imaging, separate SPADE method and orthogonalized SPADE method as a function of S. Assume we are imaging two compact sources $Q = 2$, $\sigma = 1$. We consider two different L and assume the size of the two compact source is α_1, α_2, which can be different.

C. Orthogonalized SPADE method

With the construction of orthonormal basis $\left|b_j^{(l)}\right\rangle$ from the Gram-Schmidt procedure such that

$$a_{mk,lj} = \left\langle \psi_k^{(m)} \middle| b_j^{(l)} \right\rangle \begin{cases} = 0 & m \leq l-1 \\ = 0 & m = l \ \& \ k \leq j-1 \\ \neq 0 & \text{otherwise} \end{cases} \tag{80}$$

Choose POVM as

$$\left\{ \frac{1}{2} \left|\phi_{j,\pm}^{(l)}\right\rangle \left\langle \phi_{j,\pm}^{(l)}\right|, \frac{1}{2} \left|b_j^{(0)}\right\rangle \left\langle b_j^{(0)}\right| \right\}_{j=1,2,\cdots,Q,\, l=0,1,2,\cdots,\infty}, \tag{81}$$

where

$$\left|\phi_{j\pm}^{(l)}\right\rangle = \frac{1}{\sqrt{2}} \left(\left|b_j^{(l)}\right\rangle \pm \left|b_j^{(l+1)}\right\rangle \right), \quad j = 1,2,3,\cdots,Q, \quad l = 0,1,2,\cdots,\infty \tag{82}$$

Then, the probability distribution

$$P_{0,j} = \frac{1}{2} \left\langle b_j^{(0)} \middle| \rho \middle| b_j^{(0)} \right\rangle = \sum_{q=1}^{Q} \sum_{n=0}^{\infty} c_{jnq} x_{nq} \alpha^n, \tag{83}$$

$$P_{l,j,\pm} = \frac{1}{2} \left\langle \phi_{j\pm}^{(l)} \middle| \rho \middle| \phi_{j\pm}^{(l)} \right\rangle = \sum_{q=1}^{Q} \sum_{n=2l}^{\infty} c_{ljnq}^{\pm} x_{nq} \alpha^n, \tag{84}$$

$$c_{ljnq}^{\pm} = \frac{1}{4} \sum_{m=0}^{n} \left(a_{pq,lj} a_{mq,lj}^* + a_{pq,(l+1)j} a_{mq,(l+1)j}^* \pm a_{pq,(l+1)j} a_{mq,lj}^* \pm a_{pq,lj} a_{mq,(l+1)j}^* \right) / \alpha^n, \quad p = n - m. \tag{85}$$

We set up the REC calculation as

$$D=\sum_{n=0}^{\infty}D^{(n)}=\sum_{n=0}^{\infty}\alpha^n\sum_{q=1}^{Q}d_{nq}\mathcal{C}_{nq},\quad \mathcal{C}_{nq}=\Pi_{nq}\,\mathcal{C}_{nq}^0\,\Pi_{nq}, \tag{86}$$

$$\mathcal{C}_{nq}^0=\mathrm{diag}(C_{nq}^0),\quad d_{nq}=\int d\vec{x}p(\vec{x})x_{nq}, \tag{87}$$

$$G=\sum_{n=0}^{\infty}G^{(n)}=\sum_{n=0}^{\infty}\alpha^n\sum_{n_1+n_2=n}\sum_{q_1,q_2=1}^{Q}g_{n_1q_1,n_2q_2}C_{n_1q_1}C_{n_2q_2}^T, \tag{88}$$

$$C_{nq}=\Pi_{nq}C_{nq}^0,\quad g_{n_1q_1,n_2q_2}=\int d\vec{x}p(\vec{x})x_{n_1q_1}x_{n_2q_2}, \tag{89}$$

$$C_{nq}^0=[c_{1nq},c_{2nq},\cdots,c_{Qnq},c_{01nq}^+,c_{01nq}^-,c_{02nq}^+,c_{02nq}^-,\cdots,c_{0Qnq}^+,c_{0Qnq}^-,c_{11nq}^+,c_{11nq}^-,c_{12nq}^+,c_{12nq}^-,\cdots,c_{1Qnq}^+,c_{1Qnq}^-,\cdots\cdots]^T, \tag{90}$$

$$\Pi_{nq}=\begin{bmatrix}I_{2\lfloor n/2\rfloor+1}\otimes I_Q & 0 & 0\\ 0 & I_2\otimes Q_q & 0\\ 0 & 0 & 0\end{bmatrix},\quad Q_q=\begin{bmatrix}I_q & 0\\ 0 & 0\end{bmatrix}. \tag{91}$$

We can again give an intuitive argument as in Supplementary Note 3 A and Supplementary Note 3 B about the scaling of eigenvalues β_k^2 based on characteristic polynomial. Define $P_m=\Pi_{2m,Q}-\Pi_{2(m-1),Q}$, $\Pi_{m<0,k}=0$. In the $2Q$ dimensional subspace in the support of P_m, we have vectors $P_mC_{2m,q},P_mC_{2m+1,q}$, $q=1,2,3,\cdots,Q$, $m=0,1,2,\cdots,\infty$. We will define the set of orthonormal basis $z_{2m+1,q},z_{2m+2,q}$ for convenience such that $z_{2m+1,q}$ is the Gram-Schmidt orthorgonalization of all of the $\{P_mC_{2m,q}\}_{q=1,2,3,\cdots,Q}$ and $z_{2m+2,q}$ is the Gram-Schmidt orthorgonalization of all of the $\{P_mC_{2m+1,q}\}_{q=1,2,3,\cdots,Q}$ while keeping orthogonal to $\{P_mC_{2m,q}\}_{q=1,2,3,\cdots,Q}$. Then, it should be clear that in this basis, for the (m_1,m_2)th block $W_{m_1,m_2}=P_{m_1}WP_{m_2}$, the scaling of over α is given by

$$\begin{bmatrix}W_{m_1+m_2} & W_{m_1+m_2+1}\\ W_{m_1+m_2+1} & W_{m_1+m_2+2}\end{bmatrix}, \tag{92}$$

where each block W_n is a $Q\times Q$ matrix scaling as $\Theta(\alpha^n)$. To find the scaling of eigenvalues , we again try to observe the structure of the character polynomial $|\lambda I-W|=\sum_{k=0}^{M}(-1)^k e_k\lambda^{M-k}$, its coefficients now has the form of

$$\begin{aligned}&e_1=\Theta(\alpha^0),\cdots,e_Q=\Theta(\alpha^0),\\ &e_{Q+1}=\Theta(\alpha^{0+2}),\cdots,e_{3Q}=\Theta(\alpha^{0+4Q}),\\ &e_{3Q+1}=\Theta(\alpha^{0+4Q+4}),\cdots,e_{5Q}=\Theta(\alpha^{0+4Q+8Q}),\cdots\cdots\end{aligned} \tag{93}$$

So, the scaling of eigenvalue is

$$\beta^2_{0\le i\le Q-1}=\Theta(1),\quad \beta^2_{Q\le i\le 3Q-1}=\Theta(\alpha^{-2}),\quad \beta^2_{3Q\le i\le 5Q-1}=\Theta(\alpha^{-4}),\cdots \tag{94}$$

In Supplementary Figure 9, we present the case where $\alpha=10^{-2}$ is fixed, and different numbers of compact sources Q are considered, with the centroids of these Q sources evenly distributed over $[-L/2,L/2]$. The figure shows that when the number of sources Q exceeds roughly L/Q, the stepwise increase in C_T becomes smoothed out. Note that when we fix $L=20$, the two sources in the case of $Q=2$ are sufficiently far apart. However, as Q increases to 3 or 5, the distance between the sources decreases, making the orthogonalization method more advantageous in these cases. In Supplementary Figure 10, we consider the case where $Q=2$, but α_1 and α_2 take different values for the two compact sources. It is evident that the difference in α_i between the compact sources also smooths out the stepwise increase in C_T by introducing smaller steps with varying thresholds. The prior knowledge about the moments are again chosen by randomly generating a set of images as in detailed in Supplementary Note 6.

D. Implementation of the orthogonalized SPADE method

In this section, we discuss how to implement the orthogonalized SPADE method using a Hermite-Gaussian mode sorter, beam splitters, and postselection. The central idea is that the Hermite-Gaussian mode sorter enables a transformation from Hermite-Gaussian modes into transverse momentum states. In our implementation of the orthogonalized

SPADE method, the basis functions are linear combinations of the Hermite-Gaussian modes. Therefore, the Hermite-Gaussian mode sorter enables the transformation of these orthogonalized basis functions into transverse momentum eigenstates (up to additional processing), thereby allowing full implementation of the desired measurement using only the mode sorter, beam splitter, and postselection. We illustrate this implementation with a representative example: imaging two compact sources.

When there are two compact sources located in u_1 and u_2 respectively and the PSF is given by $\psi(u) = \exp\left(-u^2/4\sigma^2\right)/(2\pi\sigma^2)^{1/4}$, for the separate SPADE measurement, the POVM as in Eq. 79 is $\left\{\frac{1}{2Q}\left|\phi_{q,m,\pm}\right\rangle\left\langle\phi_{q,m,\pm}\right|, \frac{1}{2Q}\left|b_{q,0}\right\rangle\left\langle b_{q,0}\right|\right\}_{q=1,2,\cdots,Q,\, m=0,1,2,\cdots,\infty}$, where $\left|\phi_{q,m,\pm}\right\rangle = \frac{1}{\sqrt{2}}(\left|b_{q,m}\right\rangle \pm \left|b_{q,m+1}\right\rangle)$. We can calculate the wavefunction $\left|b_{q,m}\right\rangle = \int du b_{q,m}(u)\left|u\right\rangle$ as

$$b_{q,0}(u) = \frac{\exp\left(-(u-u_q)^2/4\sigma^2\right)}{(2\pi\sigma^2)^{1/4}}, \quad b_{q,1}(u) = \frac{(u-u_q)\exp\left(-(u-u_q)^2/4\sigma^2\right)}{(2\pi)^{1/4}\sigma^{3/2}}, \quad q = 1, 2\,, \tag{95}$$

These basis functions, constructed under the assumption of a Gaussian point spread function, are the Hermite-Gaussian modes. In this subsection, we denote $b_{q,m}(u)$ as the mth Hermite-Gaussian mode centered at the centroid of the qth compact source. For POVM of the orthogonalized SPADE method given in Eq. 81, $\left\{\frac{1}{2}\left|\phi_{j,\pm}^{(l)}\right\rangle\left\langle\phi_{j,\pm}^{(l)}\right|, \frac{1}{2}\left|b_j^{(0)}\right\rangle\left\langle b_j^{(0)}\right|\right\}_{j=1,2,\cdots,Q,\, l=0,1,2,\cdots,\infty}$, where $\left|\phi_{j\pm}^{(l)}\right\rangle = \frac{1}{\sqrt{2}}\left(\left|b_j^{(l)}\right\rangle \pm \left|b_j^{(l+1)}\right\rangle\right)$, the basis functions $b_j^{(l)}(u)$ can be computed as linear combinations of the Hermite-Gaussian modes

$$\begin{aligned}
&b_1^{(0)}(u) = b_{1,0}(u), \\
&b_2^{(0)}(u) = p_1 b_{1,0}(u) + p_2 b_{2,0}(u), \quad p_1 = -\frac{e^{-(u_1-u_2)^2/8\sigma^2}}{\sqrt{1-e^{-(u_1-u_2)^2/4\sigma^2}}}, \quad p_2 = \frac{1}{\sqrt{1-e^{-(u_1-u_2)^2/4\sigma^2}}}, \\
&b_1^{(1)}(u) = p_3 b_{1,0}(u) + p_4 b_{2,0}(u) + p_5 b_{1,1}(u), \quad p_3 = \frac{\sqrt{4\sigma^2 + \frac{e^{\frac{u_1 u_2}{2\sigma^2}}(u_1-u_2)^2}{e^{\frac{u_1 u_2}{2\sigma^2}} - e^{\frac{u_1^2+u_2^2}{4\sigma^2}}}}(u_1-u_2)}{4\left(-1+e^{\frac{(u_1-u_2)^2}{4\sigma^2}}\right)\sigma^2 - (u_1-u_2)^2}, \\
&p_4 = \frac{e^{\frac{(u_1-u_2)^2}{8\sigma^2}}\sqrt{4\sigma^2 + \frac{e^{\frac{u_1 u_2}{2\sigma^2}}(u_1-u_2)^2}{e^{\frac{u_1 u_2}{2\sigma^2}} - e^{\frac{u_1^2+u_2^2}{4\sigma^2}}}}(-u_1+u_2)}{4\left(-1+e^{\frac{(u_1-u_2)^2}{4\sigma^2}}\right)\sigma^2 - (u_1-u_2)^2}, \quad p_5 = -\frac{2\left(-1+e^{\frac{(u_1-u_2)^2}{4\sigma^2}}\right)\sigma\sqrt{4\sigma^2 + \frac{e^{\frac{u_1 u_2}{2\sigma^2}}(u_1-u_2)^2}{e^{\frac{u_1 u_2}{2\sigma^2}} - e^{\frac{u_1^2+u_2^2}{4\sigma^2}}}}}{4\left(-1+e^{\frac{(u_1-u_2)^2}{4\sigma^2}}\right)\sigma^2 - (u_1-u_2)^2}.
\end{aligned} \tag{96}$$

Higher-order basis states $\left|b_q^{(l)}\right\rangle$ can be constructed similarly, though their explicit forms become too complex to present here.

There has been significant discussion regarding Hermite-Gaussian mode sorting [37–41], which enables the conversion of Hermite-Gaussian modes, with $m = 0, 1, 2, \ldots, \infty$ for a fixed q, into transverse momentum states. Note that when the PSF is Gaussian, $b_{q,m}(u)$ in Eq. 95 corresponds to the Hermite-Gaussian mode. The transformation from Hermite-Gaussian modes to momentum states can be expressed as $\left|m\right\rangle = F_q\left|b_{q,m}\right\rangle$. Since the orthogonalized SPADE basis $b_j^{(l)}(u)$ can be written as a linear combination of these Hermite-Gaussian modes $b_{q,m}(u)$, the mode converter F_q, allows for transforming the basis $b_j^{(l)}(u)$ into simple transverse momentum states with some addition steps, thereby enabling complete measurement.

Supplementary Figure 11 illustrates the process of generating superpositions of nonorthogonal spatial modes $\left|\psi_{a,b,c}\right\rangle$, where each spatial mode $\left|\psi_{a,b,c}\right\rangle$ can be created using the mode converter $F_{a,b,c}^{\dagger}$ from a single photon in momentum states. Examples are shown for the cases of two and three distinct spatial modes, which can be easily extended to include additional modes. For the implementation of $\left|\phi_{j\pm}^{(l)}\right\rangle$, we will need the spatial modes $b_{q,m}(u)$ for $q = 1, 2, \cdots, Q$ when $m \le l$, and $q \le j$ when $m = l+1$. This procedure can be reversed to detect spatial modes as linear combinations of $\left|b_{q,m}\right\rangle$. Since both $\left|\phi_{j,\pm}^{(l)}\right\rangle$ and $\left|b_j^{(l)}\right\rangle$ can be expressed as linear combinations of $\left|b_{q,m}\right\rangle$, the orthogonalized SPADE method can be implemented using the Hermite-Gaussian mode converter with some additional steps.

The basis $\left|b_j^{(l)}\right\rangle$ can be constructed differently from Eq. (80) while preserving the key feature of the orthogonalized SPADE method—namely, ensuring that lower-order terms vanish in the probability distribution when estimating higher-order moments. This design enhances the performance of moment estimation. For instance, one could redefine the basis $\left|\psi_k^{\prime(m)}\right\rangle$ as a linear combination of $\left|\psi_{j=1,2,\ldots,Q}^{(m)}\right\rangle$ in the Eq. (80) and then construct $\left|b_j^{(l)}\right\rangle$ using the

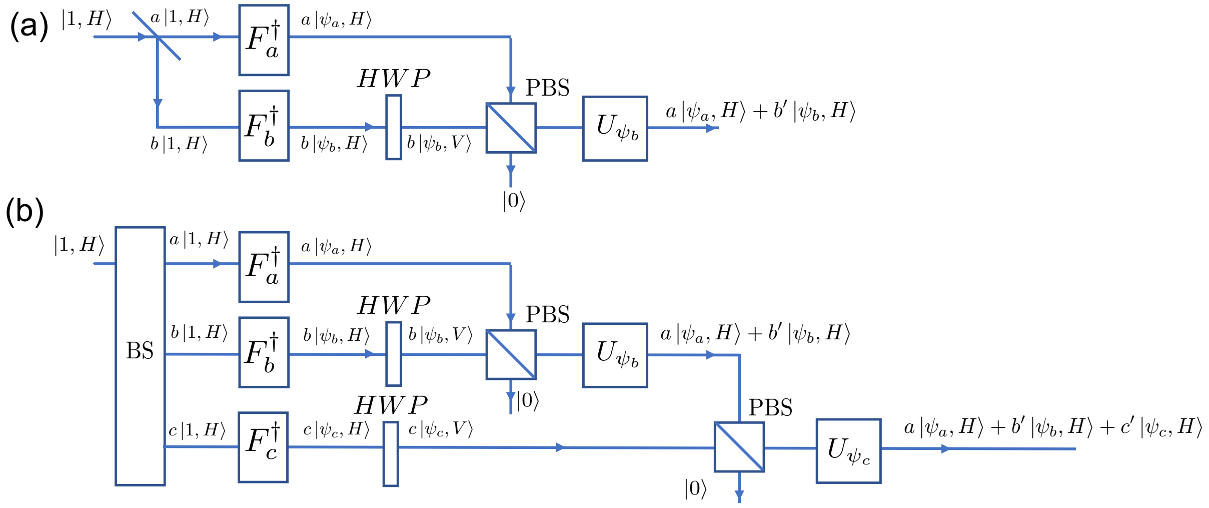

$$a\,|\psi_a,H\rangle = a_1\,|\psi_a^{\perp},H\rangle + a_2\,|\psi_b,H\rangle$$
$$a\,|\psi_a,H\rangle = a_3\,|\psi_a'^{\perp},H\rangle + a_4\,|\psi_c,H\rangle$$
$$b'\,|\psi_b,H\rangle = b_1'\,|\psi_b^{\perp},H\rangle + b_2'\,|\psi_c,H\rangle$$

$$U_{\psi_b} = \frac{1}{\sqrt{|a_2|^2+|b|^2}}\begin{bmatrix} a_2^* & b^* \\ -b & a_2 \end{bmatrix} \begin{matrix} |\psi_b,H\rangle \\ |\psi_b,V\rangle \end{matrix} \qquad b' = \sqrt{|a_2|^2+|b|^2} - a_2$$

$$U_{\psi_c} = \frac{1}{\sqrt{|a_4+b_2'|^2+|c|^2}}\begin{bmatrix} a_4^*+b_2'^* & c^* \\ -c & a_4+b_2' \end{bmatrix} \begin{matrix} |\psi_c,H\rangle \\ |\psi_c,V\rangle \end{matrix} \qquad c' = \sqrt{|a_4+b_2'|^2+|c|^2} - a_4 - b_2'$$

Supplementary Figure 11. Generation of a superposition of (a) two and (b) three nonorthogonal spatial modes using the mode converter $F_{a,b,c}$, which transforms the nonorthogonal spatial modes $|\psi_{a,b,c}\rangle$ into transverse momentum states. The labels H and V denote the two polarizations. The setup includes polarizing beam splitter (PBS), beam splitter (BS), and half-wave plate (HWP). The operation $U_{\psi_{b,c}}$ is applied to the two polarizations of the spatial mode $|\psi_{b,c}\rangle$ to achieve the desired transformation.

Gram-Schmidt procedure applied to the set of basis states $\left|\psi_k'^{(m)}\right\rangle_{k=1,2,\ldots,Q;,m=0,1,2,\ldots}$. This approach allows for the exploration of alternative constructions of $\left|b_j^{(l)}\right\rangle$. As the current construction is not necessarily the optimal choice for experimental implementation, the flexibility in constructing the basis $\left|b_j^{(l)}\right\rangle$ could potentially lead to simpler or more practical implementations of the orthogonalized SPADE method.

The implementation of the measurement must be sufficiently accurate to ensure that any deviations in the probability distribution due to imprecision remain smaller than the lowest-order terms of α for each outcome. Consequently, the precision requirements for the measurement become increasingly stringent for higher-order moments.

E. Eigenvectors and eigenvalues for multiple compact sources with varying position distributions

In the case of imaging multiple compact sources, the eigentasks generally take on very diverse forms in practical scenarios. In fact, the form of the eigentasks is strongly influenced by the practical imaging model and the prior knowledge, such as the positions of the individual sources. As previously noted, this is an important aspect of the problem: when the number of samples is finite, it is unclear which features can be accurately estimated, and identifying these features is nontrivial. This is crucial because, in downstream analysis, it is essential to know which measured features can be reliably used.

We present the eigentasks corresponding to three different configurations of four compact sources in Supplementary Figure 12(a1)-(a3). The numerical simulation procedure follows that described in Supplementary Note 6: prior information is obtained by randomly generating a set of source distributions, which are then used to compute the sampled values of d_n and g_{mn}. We assume the presence of four compact sources, each confined to a region indicated by the function $\zeta(u)$ in the figure. Note that the intensity distributions within each $\zeta(u) = 1$ region are randomly generated; we are not imaging four simple sources with flat intensity profiles. Instead, each region where $\zeta(u) = 1$ may exhibit more complex internal structure, while any region with $\zeta(u) = 0$ has zero intensity.

Let us first consider the direct imaging method, as shown in Supplementary Figure 12(b1)–(b3). The eigenvectors r_k, obtained by solving the generalized eigenvalue equation $Vr_k = \beta_k^2 G r_k$ in Eq. 14, are displayed in the figure. In the direct imaging method, each measurement outcome corresponds to the probability of detecting a photon at a position

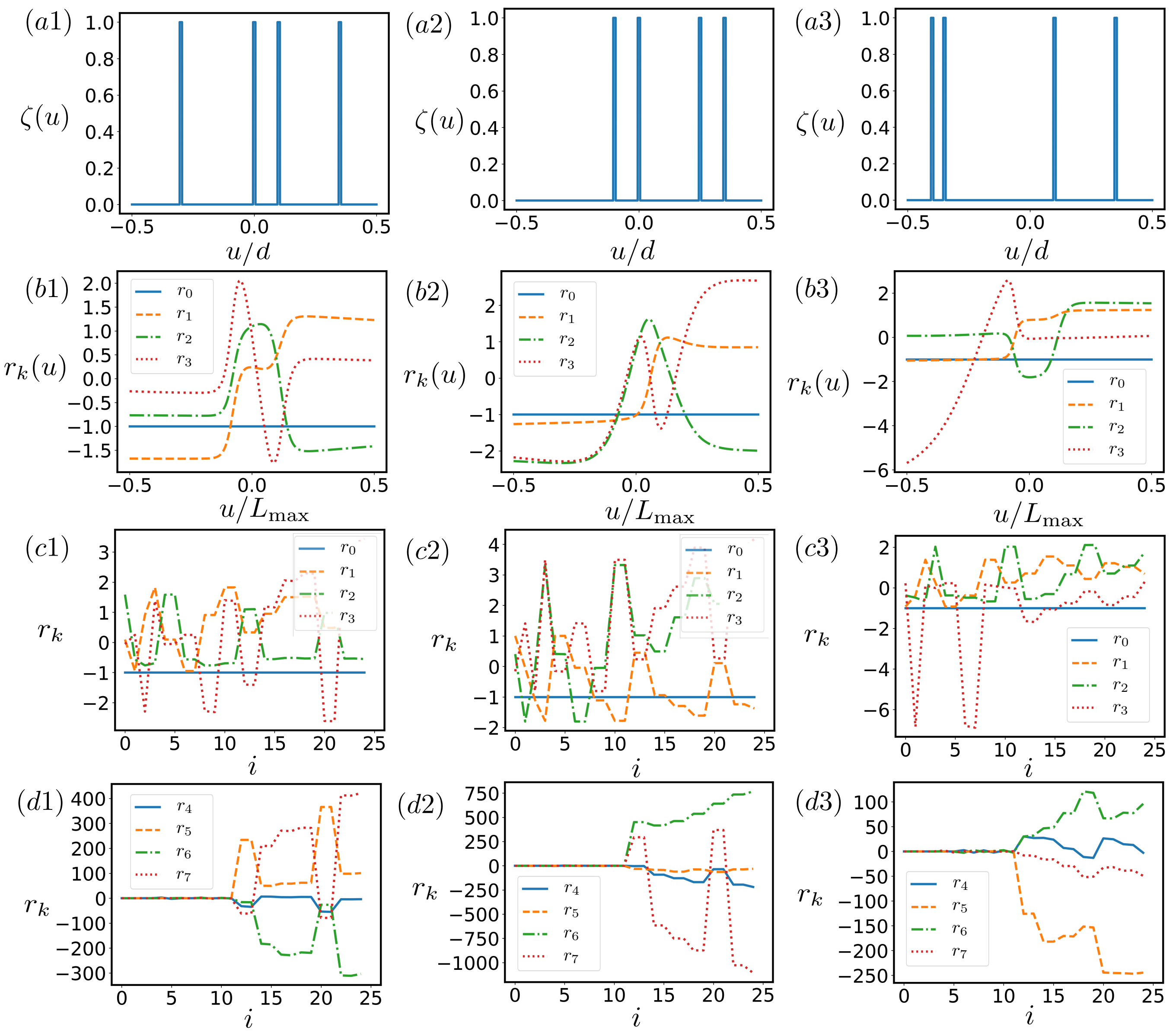

Supplementary Figure 12. (a1)–(a3) Different positions of the four compact sources, indicated by $\zeta(u)$. The x-axis indicates the position u on the image plane. (b1)–(b3) The first four eigenvectors r_k for direct imaging. The x-axis indicates the position u on the detection plane. (c1)–(c3), (d1)–(d3) The first eight eigenvectors r_k for the orthogonalized SPADE method. The outcomes for the orthogonalized SPADE method are labeled in the following order: $P_{01}, \cdots, P_{04}, P_{0,1,+}, P_{0,1,-}, \cdots, P_{0,4,+}, P_{0,4,-}, P_{1,1,+}, P_{1,1,-}, \cdots, P_{1,4,+}, P_{1,4,-}, \ldots$, where the meaning of each subscript in $P_{l,j,\pm}$ is defined in Eq. 83. We set $\alpha = 10^{-1}$ for these figures. The centroids of all compact sources are confined within $[-d/2, d/2]$ with $d = 10$, the width of the PSF is $\sigma = 1$, and the size of each source is $\alpha\sigma = 0.1$. The region $[-L_{\max}/2, L_{\max}/2]$ on the image plane is detected with $L_{\max} = d + 10\sigma$.

u on the image plane. The k-th eigentask is given by $\sum_i r_k(u_i)P(u_i)$, where $P(u_i)$ is the probability of detecting a photon at position u_i. It is evident that the eigentasks are strongly influenced by the positions of the compact sources.

We next examine the eigentasks of the orthogonalized SPADE method. From Supplementary Figure 12(c1)–(c3), we observe the first four eigenvectors of the orthogonalized SPADE method. As discussed in the main text, for four compact sources, we expect four eigentasks corresponding to the Rayleigh-resolvable features. In general, these eigentasks incorporate all measurement outcomes of the constructed orthogonalized SPADE method, particularly including the first few outcomes that scale as $\Theta(1)$. From Supplementary Figure 12(d1)–(d3), we observe the next four eigenvectors of the orthogonalized SPADE method, which correspond to the sub-Rayleigh features. Note that

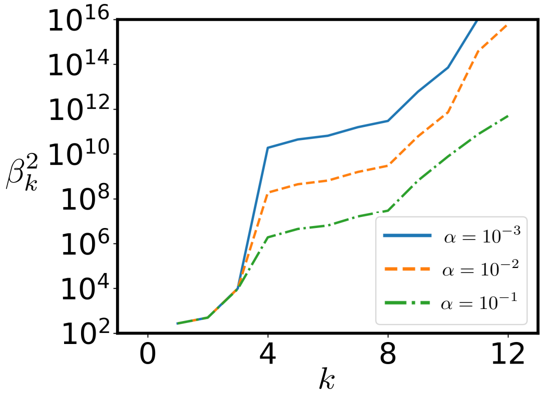

Supplementary Figure 13. Eigenvalues β_k^2 for various k and different α using the orthogonalized SPADE method to image four compact sources. The simulation setup is identical to that in Supplementary Figure 12(a3). The value $\beta_0^2 = 0$ is omitted from the plot.

the eigenvectors r_k are normalized such that $r_{k_1}^T D r_{k_2} = \delta_{k_1 k_2}$. Consequently, some components of r_k can take very large values because certain elements of D in the orthogonalized SPADE method can be quite small, owing to the α^{2n} scaling in D. A clear characteristic of these eigentasks is that they have vanishing components in the first few outcomes. This is expected because the construction of these eigentasks explicitly cancels the lower-order terms in α, as shown in Eq. 83, and we therefore anticipate corresponding structures in the eigenvectors r_k. Direct computation of the eigenvalue problem confirms this expectation. Clearly, the eigentasks exhibit complex structures that are strongly influenced by the positions of the compact sources. This complexity becomes increasingly important as the number of compact sources grows, since the prefactors accompanying the α-dependent terms also affect the thresholds, especially when α is not very small. Selecting eigentasks along with their corresponding thresholds is therefore crucial for the downstream analysis of features extracted from the images.

As shown in Supplementary Figure 13, we plot the eigenvalues β_k^2 obtained using the orthogonalized SPADE method to image four compact sources. These results reveal that although, when α is sufficiently small, the α-scaling dominates the behavior of β_k^2, for the multiple compact source problem the prefactors can also lead to an increase in β_k^2 at higher-order eigenvalues. See Supplementary Note 7 for a more systematic study of these prefactors. In practice, we view the multiple compact source model as an approximation of more general sources exhibiting clustered structures, or as a potential approach to tackle general imaging problems after further development of the orthogonalized SPADE method, which aims to resolve fine details of the source even when its overall extent exceeds the Rayleigh limit. This suggests that the number of compact sources can be large in practical tasks. It is therefore essential to identify the eigentasks that can be reliably estimated given a finite sample size in such scenarios involving multiple compact sources. In general, these eigentasks incorporate contributions from all the compact sources, determined by the prior information.

Supplementary Note 6. DETAILS OF NUMERICAL CALCULATIONS

In our numerical calculations for the direct imaging case, we discretize the spatial coordinate for analysis, which is reasonable since, in practice, direct imaging involves pixels of finite size. Assuming the centroid of all compact sources or point sources is within the interval $[-d/2, d/2]$, we extend this range to $[-d/2 - 5\sigma, d/2 + 5\sigma]$ for the PSF given by $\psi(u) = \frac{\exp\left(-u^2/4\sigma^2\right)}{(2\pi\sigma^2)^{1/4}}$. Because of the exponential decay of the PSF, this cutoff is expected to have a negligible impact on the results. We then select $T_{\max}$ points at $X_1, X_2, \cdots, X_{T_{\max}}$ uniformly distributed across this interval, forming the set of POVMs $\{M_i\}_{i=1,2,\ldots,T\max}$, where each $M_i = \int_{X_i-l/2}^{X_i+l/2} dx \, |x\rangle \langle x|$ and $l = (d + 10\sigma)/T_{\max}$. In the calculations presented in the main text, we use $T_{\max} = 50$, as further increasing $T_{\max}$ does not show obvious difference. A plot illustrating the convergence of our calculations for different $T_{\max}$ values confirms the stability of the results. Using the imaging of a single compact source as an example (Supplementary Figure 14), we show that varying $T_{\max}$ does not affect the scaling behavior of β_k^2 with α or the stepwise increase of C_T with S. In theory, C_T should increase slightly with larger $T_{\max}$ values due to the greater number of independent pixels; however, this effect is minimal in the numerical calculation. This is likely because, for compact source imaging, the information contained in the nearby pixel is largely similar.

$m\backslash l$	0	1	2	3	4
0	1	0	0	0	0
1	0	$\frac{1}{2}\alpha$	0	0	0
2	$-\frac{1}{8}\alpha^2$	0	$\frac{1}{4\sqrt{2}}\alpha^2$	0	0
3	0	$-\frac{1}{16}\alpha^3$	0	$\frac{1}{8\sqrt{6}}\alpha^3$	0
4	$\frac{1}{128}\alpha^4$	0	$-\frac{1}{32\sqrt{2}}\alpha^4$	0	$\frac{1}{32\sqrt{6}}\alpha^4$

Supplementary Table III. $a_{ml} = \left\langle \psi^{(m)} \middle| b_l \right\rangle$ for the coefficient Eq. 97.

For imaging of one compact source using the SPADE method [4], assume the PSF is again given by $\psi(u) = \exp\left(-u^2/4\sigma^2\right)/(2\pi\sigma^2)^{1/4}$. Using $\rho = \sum_{m,n=0}^{\infty} x_{m+n} \left|\psi^{(m)}\right\rangle \left\langle \psi^{(n)}\right|$, we will construct the orthonormal measurement basis $\{|b_l\rangle\}_l$ such that

$$a_{ml} = \left\langle \psi^{(m)} \middle| b_l \right\rangle = \begin{cases} = 0 & m \le l-1 \\ \neq 0 & m \ge l \end{cases} \tag{97}$$

We can explicitly calculate the coefficients a_{ml}, the first few coefficients is shown in Table Supplementary Table III as examples. If we define the measurement as in Eq. 5, the probability distribution of getting $|b_0\rangle$, $|\phi_{k\pm}\rangle$, $k = 0, 1, 2, \cdots$

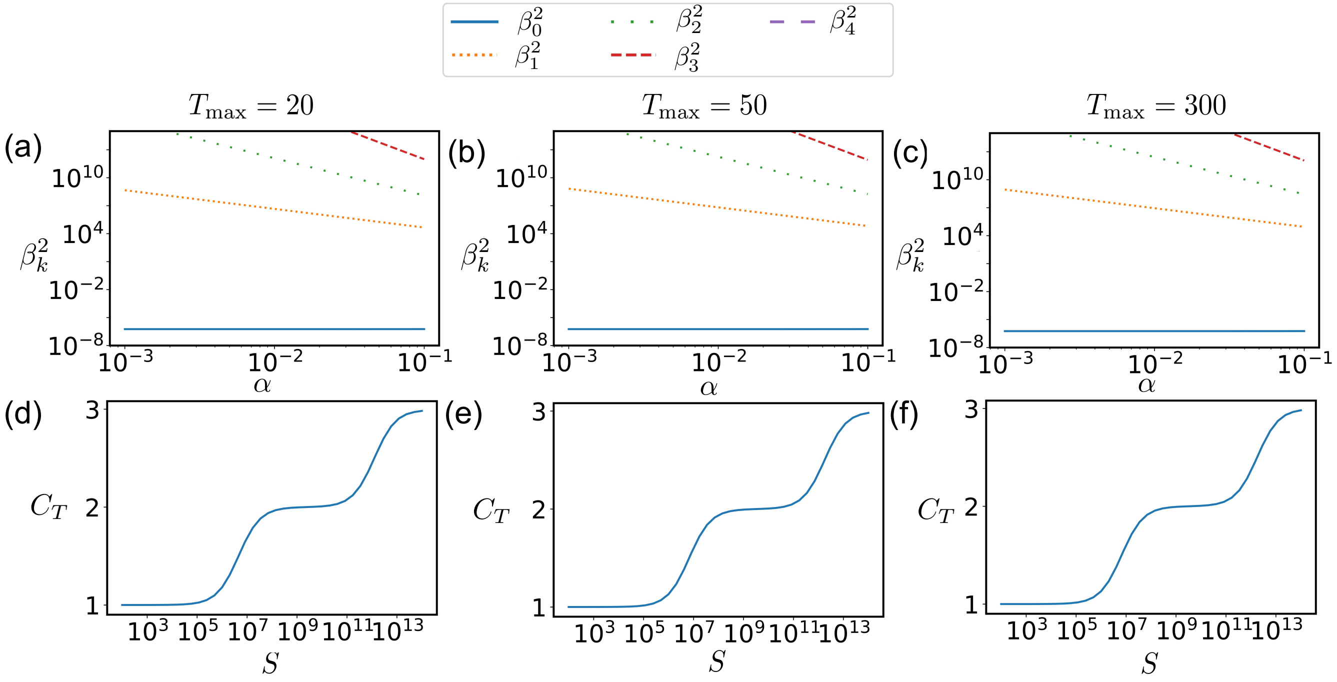

Supplementary Figure 14. Robustness of the numerical calculation for direct imaging with different discretization $T_{\max}$. The calculation is done for one compact source, and for (d)-(e) we choose $\alpha = 10^{-2}$. (Note in this subsection, we include β_0^2 in the plot for β_k^2 as a function of α, which numerically appears slightly above zero due to finite numerical precision. This discrepancy could be minimized by increasing the calculation's precision. Additionally, we do not interpolate values of β_k^2 above 10^{15} with a straight line as in the main text; instead, we introduce a direct cutoff at 10^{15}, beyond which numerical calculations lack sufficient precision.)

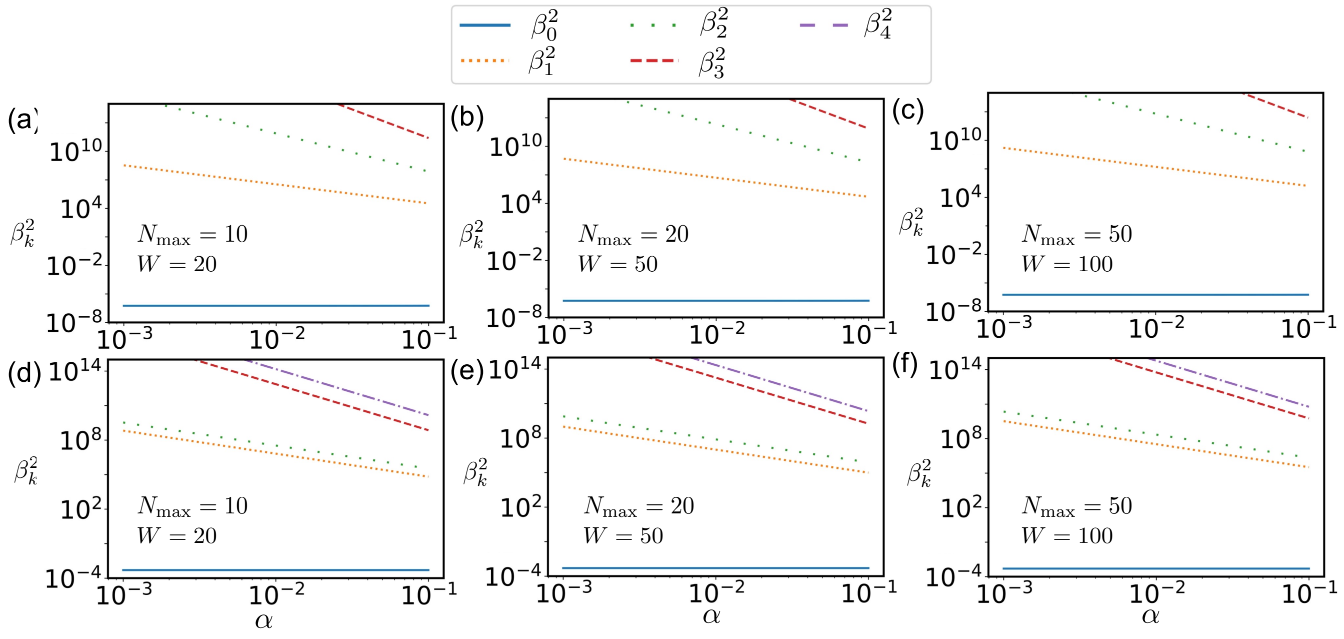

Supplementary Figure 15. The numerical calculation of β_k^2 performed using various randomly generated sets of prior information, with different values for $N_{\max}$ and W. We analyze the imaging of a single compact source using direct imaging and the SPADE method. β_4^2 exceeds the cutoff at 10^{15} for (a)-(c). In the main text, this line is interpolated using calculations for $\alpha > 0.1$.

is given by

$$P_0 = \frac{1}{2}\sum_{m,n=0}^{\infty} x_{m+n} a_{m,0}^* a_{n,0},$$
$$P_{k,+} = \frac{1}{4}\sum_{m,n=0}^{\infty} x_{m+n}(a_{m,k}^* a_{n,k} + a_{m,k+1}^* a_{n,k+1} + a_{m,k+1}^* a_{n,k} + a_{m,k}^* a_{n,k+1}), \tag{98}$$
$$P_{k,-} = \frac{1}{4}\sum_{m,n=0}^{\infty} x_{m+n}(a_{m,k}^* a_{n,k} + a_{m,k+1}^* a_{n,k+1} - a_{m,k+1}^* a_{n,k} - a_{m,k}^* a_{n,k+1}).$$

By comparing this concrete form of probability with the abstract form used earlier, $P_0 = \sum_{m=0}^{\infty} c_m x_m \alpha^m$, $P_{k,\pm} = \sum_{m\geq 2k} c_{km}^{\pm} x_m \alpha^m$ as in Eq. 7, we can determine the individual coefficients $c_{nm}^{\pm}$.

To perform the numerical calculations for both single and multiple compact sources, we randomly generate prior knowledge $p(\vec{x})$ over the input moment vectors $\boldsymbol{\theta} = \vec{x} = [x_0, x_1, x_2, \dots]$. This is done by first generating a set of W one-dimensional images, each of which yields a corresponding moment vector $\vec{x}(\omega)$ that serves as a sample of input parameters, where ω indexes each generated vector. The prior distribution $p(\vec{x})$ is then defined by the empirical statistics of this ensemble. This prior is used to compute the quantities $d_n = \int d\vec{x} p(\vec{x}) x_n$ and $g_{n_1 n_2} = \int d\vec{x} p(\vec{x}) x_{n_1} x_{n_2}$, which enter subsequent stages of the calculation. Numerically, these matrix elements are estimated by averaging over the sampled moment vectors: $d_n = \frac{1}{W}\sum_w x_n(w)$ and $g_{n_1 n_2} = \frac{1}{W}\sum_w x_{n_1}(w) x_{n_2}(w)$. This sampling-based approach enables efficient incorporation of general prior knowledge into the numerical evaluation of the quantities required for our analysis.

To generate these images, we assume that the qth compact source is confined to a region of size L_q, which is divided into $N_{\max}$ equal segments. A point source is placed at the center of each segment, with an assigned intensity I_m drawn uniformly from the interval $[0, 1]$. As $N_{\max}$ becomes sufficiently large, this setup provides high degrees of freedom to model generally distributed compact sources. After assigning random intensities I_m, we normalize them by replacing each I_m with $I_m/(\sum_m I_m)$ so that the total intensity sums to one. Because each point source has a known coordinate, we can compute the corresponding moment vector $\vec{x}(w)$ for each generation. By repeating this procedure, we obtain a set of W moment vectors $\vec{x}(w)$ for use in statistical averaging. In the calculations presented in the main text, we set $N_{\max} = 20$ and $W = 50$, meaning each compact source is modeled by 20 evenly spaced pixels, and 50 different

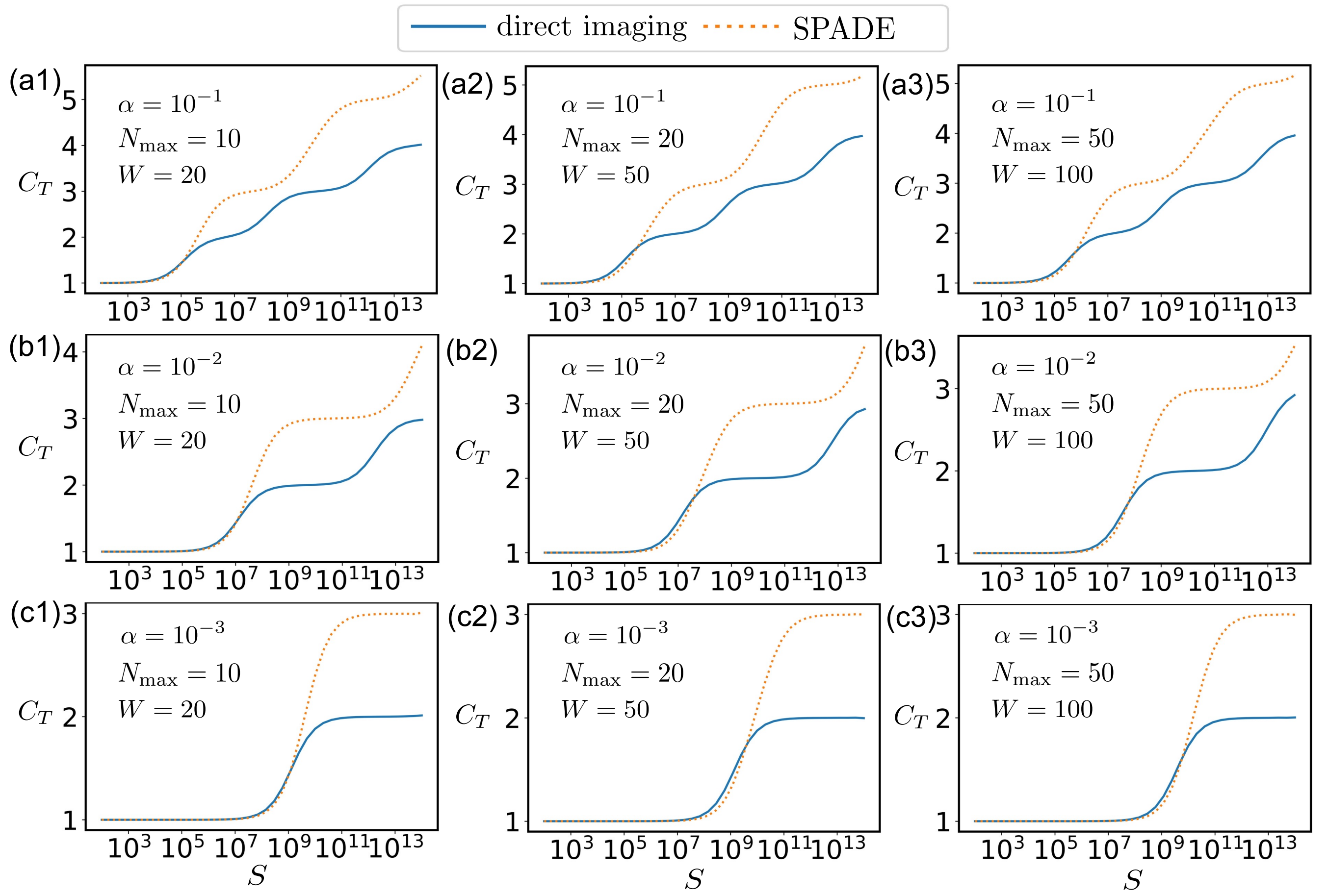

Supplementary Figure 16. The numerical calculation of REC C_T as a function of S performed using various randomly generated sets of prior information, with different values for $N_{\max}$ and W. We analyze the imaging of a single compact source using direct imaging and the SPADE method.

moment vectors are generated to compute d_n and $g_{n_1 n_2}$. For scenarios involving multiple compact sources whose centroids are separated by a distance L and individual sizes $L_q \ll L$, we generate nonzero intensity only within each compact region of size L_q. The intensities are normalized such that the total intensity across all compact sources sums to one.

We emphasize that these randomly generated sources serve as a simple example to simulate prior knowledge and to demonstrate our approach. However, the discussion in the main text applies to any general prior information about the moments $\vec{x}$. It is important to note that a set of images is needed to generate the matrices $g_{n_1 n_2}$ and d_n; using only one image would make $g_{n_1 n_2}$ a rank-one matrix, implying that the moment of the image is already known, hence no additional information can be obtained through measurement.

We examine the impact of varying M and W on both the scaling of β_k^2, shown in Supplementary Figure 15, and the C_T vs. S plot, shown in Supplementary Figure 16. As we increase $N_{\max}$ and W, the scaling of β_k^2 with α and the stepwise increase of C_T as a function of S remains the same. However, a careful reader may observe that as $N_{\max}$ and W grow, β_k^2 exhibit a slight increase, as can be seen by comparing Supplementary Figure 15(a) with Supplementary Figure 15(c). This trend is also evident in Supplementary Figure 16(c1) and Supplementary Figure 16(c3), where the threshold for each stepwise increment rises. This effect occurs because the prior knowledge influences the prefactor of the eigenvalues β_k^2. Numerically, we observe the prefactors of β_k^2 tend to increase as the eigenvalues of $g_{n_1 n_2}$ decrease. If $g_{n_1 n_2}$ is full rank, the prior information is not expected to influence the scaling of β_k^2.

The method described above generates a unique random prior knowledge for each execution, resulting in a different set of g and d for every run. In all the discussions in this work, the plots are generated using a single instance of randomly generated prior knowledge. To verify that our method works universally for any prior information, we run the code multiple times and observe that each instance of prior knowledge produces a similar scaling of β_k^2 with α, as expected. Consequently, C_T consistently displays a stepwise increase as a function of S. In Supplementary Figure 17,

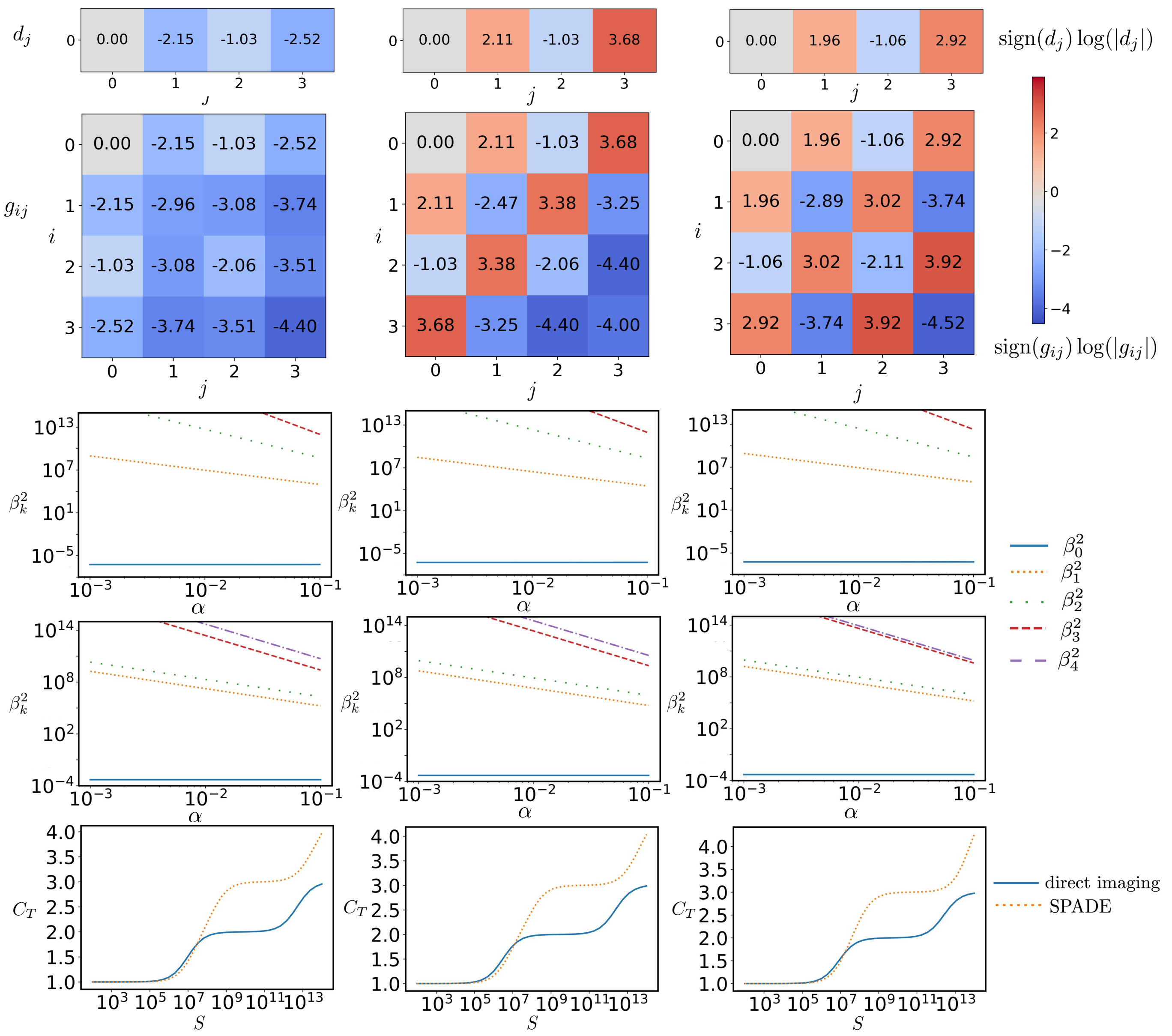

Supplementary Figure 17. The numerical calculation of REC C_T as a function of S when $\alpha = 10^{-2}$ and β_k^2 as a function of α. The calculation is performed using three different prior information randomly generated when $N_{\max} = 10$, $W = 20$. The first few elements of d, g for these three different prior information are given. We analyze the imaging of a single compact source using direct imaging and the SPADE method.

we present results for three randomly generated instances of prior knowledge using this method. In these three instances, g and d differ due to the difference of prior information. While the eigenvalues and C_T vary with the prior knowledge, the scaling behavior of β_k^2 remains consistent, ensuring the stepwise increase of C_T is always observed.

Supplementary Note 7. DISCUSSION ABOUT THE PREFACTORS

In this section, we discuss the prefactors of β_k^2, complementing the main text, which primarily focuses on the scaling of β_k^2 with respect to α in different scenarios. For convenience, we summarize the eigenvalue problems from Eq. 14,

Eq. 27, and Eq. 47 below

$$\begin{aligned} &V = D - G, \quad Vr_k = \beta_k^2 G r_k, \quad G r_k = \lambda_k D r_k, \quad \lambda_k = (1+\beta_k^2)^{-1}, \\ &D = \sum_{n=0}^{\infty} d_n \alpha^n \mathcal{C}_n, \quad G = \sum_{n=0}^{\infty} \alpha^n \sum_{i+j=n} g_{ij} C_i C_j^T. \end{aligned} \tag{99}$$

In general, it is difficult to extract the exact values of the prefactors analytically. However, we can analyze a special case that offers clear intuition and, in some situations, serves as a good approximation to the actual prefactor values in β_k^2. We redefine

$$\begin{aligned} &W = D^{-1/2} G D^{-1/2}, \quad W y_k = \lambda_k y_k, \quad y_k = D^{1/2} r_k, \\ &W = \sum_{n=0}^{\infty} \alpha^n \sum_{i+j=n} g_{ij} (D^{-1/2} C_i)(D^{-1/2} C_j)^T = \mathbb{C}^T g \mathbb{C}, \\ &\mathbb{C} = [D^{-1/2} C_0, \alpha D^{-1/2} C_1, \alpha^2 D^{-1/2} C_2, \cdots] \\ &\mathbf{C} g v_k = \lambda_k v_k, \quad \mathbf{C} = \mathbb{C}\mathbb{C}^T \end{aligned} \tag{100}$$

where we use the fact that the eigenvalues of AB and BA are equal [42]. Therefore, the analysis of the eigenvalues β_k^2 reduces to examining the spectrum of the product of the two matrices $\mathbf{C}g$. In general, this is still difficult to compute. However, in the special case where $\mathbf{C}$ and g commute, we can choose v_k to be the common eigenvectors of both $\mathbf{C}$ and g. In this case, the eigenvalues satisfy $\lambda_k = \lambda_k(\mathbf{C})\lambda_k(g)$, so the spectrum is directly determined by the eigenvalues of $\mathbf{C}$ and g.

Of course, in general, $\mathbf{C}$ and g do not commute with each other. However, in the following, we numerically show that the product of the eigenvalues of $\mathbf{C}$ and g provides a good approximation to the actual values of λ_k in some examples. As shown in Supplementary Figure 18, we plot the eigenvalues of g and $\mathbf{C}$ for both the direct imaging and SPADE methods. The simulation consists of 10 successive runs of such simulations, where each run uses a randomly generated prior, as explained in Supplementary Note 6. For each run, we randomly generate a set of $W = 5$ one-dimensional images and use the corresponding moment vectors to calculate d and g by averaging, as described in Supplementary Note 6. It is clear that the eigenvalues $\lambda_k(g)$ of the matrix g fluctuate randomly. The eigenvalues $\lambda_k(\mathbf{C})$ of the matrix $\mathbf{C}$ for both the direct imaging and SPADE methods used for imaging a single compact source are shown for each run. We observe that the eigenvalues $\lambda_k(\mathbf{C})$ do not vary much under the randomly generated priors considered here. We also plot the prefactor $\tau_k = \lambda_k/\alpha^{n_k}$, where $n_k = [0, -2, -4, -6, \cdots]$ for direct imaging and $n_k = [0, -2, -2, -4, -4, \cdots]$ for SPADE method. We find that the variation of τ_k is mainly determined by $\lambda_k(g)$ for a given approach, since $\lambda_k(\mathbf{C})$ does not vary much with the prior considered here; however, τ_k can differ significantly between approaches due to differences in $\lambda_k(\mathbf{C})$. Clearly, the variation of τ_k computed using the approximate $\hat{\lambda}_k = \lambda_k(\mathbf{C})\lambda_k(g)$ for both direct imaging and SPADE closely follows that obtained using the actual eigenvalues λ_k. We find that this approximation is reasonably good and can serve as an intuitive guide for understanding the spectrum of β_k^2 in the examples considered here. Note that $\lambda_k = (1+\beta_k^2)^{-1}$, so when $\alpha \ll 1$, $\tau_{k\geq 1}^{-1}$ is roughly the prefactor of β_k^2. We choose to plot the prefactors of λ_k because τ_k exhibits the same trend as the eigenvalues $\lambda_k(g)$, while the prefactors of β_k^2 show the inverse trend. Thus, it is easier to observe the behavior of the prefactors using λ_k.

Note $g_{ij} = \int d\vec{x}\, p(\vec{x}) x_i x_j$ is the matrix that directly quantifies our prior knowledge of the moments of the images. The matrix $\mathbf{C}$ also incorporates some prior information, but further includes α and the vectors C_i, which are directly related to the measurement strategy. In other words, different approaches, such as direct imaging and SPADE methods, have the same g but different $\mathbf{C}$. In this sense, we can intuitively think of g as quantifying the prior information, while $\mathbf{C}$ quantifies the measurement strategy. Note that $\mathbf{C}$ also depends on the prior information through its dependence on D, but numerical analysis above suggests that this dependence may not be very strong in some cases. Thus, this analysis offers an intuitive and heuristic way to understanding the behavior of the prefactors, highlighting the two main factors that influence β_k^2: the measurement strategy, which primarily determines $\mathbf{C}$, and the prior information, which determines g. Note that the vectors C_i, which constitute $\mathbf{C}$, are the coefficients of each order term in the series expansion of the probability distribution in powers of α. Intuitively, larger eigenvalues of $\mathbf{C}$ indicate that the C_i are more independent, meaning that the probability distributions exhibit more distinct behavior in each coefficient. Consequently, the measurement outcomes may contain more information, as reflected by the larger eigenvalues of $\mathbf{C}$. Lastly, we emphasize that the approximate value $\hat{\lambda}_k = \lambda_k(g)\lambda_k(\mathbf{C})$ is meant solely as an intuitive tool to understand the behavior of the prefactors of β_k^2, inspired by a special case that g and $\mathbf{C}$ commute with each other. We do not claim that this approximation is universally valid for all prior information and measurement strategies.

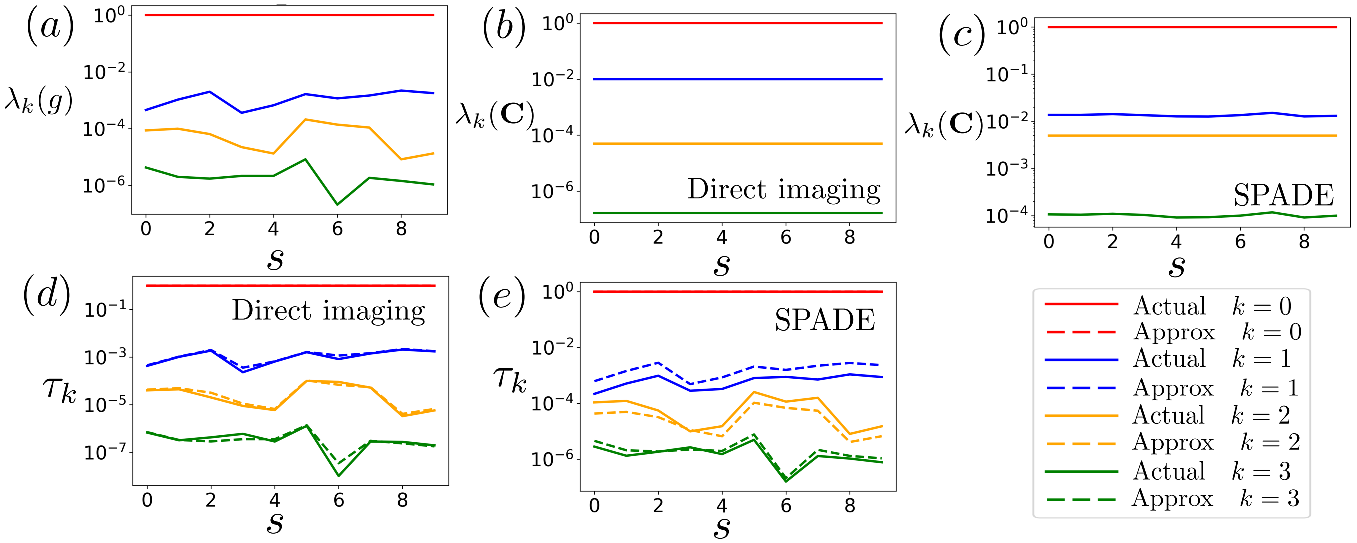

Supplementary Figure 18. (a) Eigenvalues of g. (b)–(c) Eigenvalues of $\mathbf{C}$ for direct (b) and SPADE (c) imaging of a compact source. (d) Prefactors of λ_k for direct imaging, defined as $\tau_k = \lambda_k/\alpha^{n_k}$, where $n_k = [0, -2, -4, -6, \cdots]$. (e) Prefactors of λ_k for the SPADE method, defined as $\tau_k = \lambda_k/\alpha^{n_k}$ with $n_k = [0, -2, -2, -4, -4, \cdots]$. We fix $\alpha = 0.1$. All plots are computed over 10 successive runs of the simulation with randomly generated prior distributions, labeled by s (the s-th run). Here, k labels the k-th eigenvector. The dashed lines represent the prefactors computed using the approximate value $\hat{\lambda}_k = \lambda_k(g)\lambda_k(\mathbf{C})$, while the solid lines represent the prefactors computed using the actual eigenvalues λ_k.

Supplementary Note 8. DEMONSTRATIVE EXAMPLE

In this section, we provide the simulation details of the demonstrative examples that illustrate the use of the quantum learning approach and our new orthogonalized SPADE method to perform a distinguishing task with a highly complex structure, inspired by the pattern of a QR code and representative of challenges encountered in practical applications. We begin with the imaging of a general source beyond the Rayleigh limit, then discuss the imaging of multiple compact sources, and finally highlight the advantages of the quantum learning approach in addressing such complex imaging problems.

Algorithm 1 Demonstrative Example Procedure

Input: Number of pixels N, interval size L, activation probability p_0, number of training samples per class W_{train}, number of test samples per class W_{test}, total detected photons per test image S, truncation order $\mathcal{K}$
Define indicator function $\eta(u) \in \{0,1\}$ over $[-L/2, L/2]$ to specify active regions.
Training:
for each class (Case 1: active; Case 2: inactive) **do**
  for $i = 1$ to W_{train} **do**
    for each pixel $j = 1$ to N **do**
      if Case 1 and $\eta(u_j) = 1$ **then**
        Set $I_i(u_j) = 1$ with probability p_0, else 0
      else if Case 2 and $\eta(u_j) = 0$ **then**
        Set $I_i(u_j) = 1$ with probability p_0, else 0
      else
        Set $I_i(u_j) = 0$
      end if
    end for
    Normalize $I_i(u)$ such that $\sum_{j=1}^{N} I_i(u_j) = 1$, which determines the nonzero value of $I_i(u_j)$ as i_0.
  end for
end for
Compute prior matrices d_k and g_{ij} from all training samples, taking the prior to be the uniform distribution over the training set.
Solve eigenvalue problem (Eq. 14) to obtain eigenvectors r_k and eigenvalues β_k^2.
for each training sample i in both classes **do**
  Compute theoretical detection probabilities $P_i(u_j)$.
  Compute eigentask components $\xi_{ki} = \sum_j r_{kj} P_i(u_j)$.
  Form truncated eigentask vector $\vec{\xi}_i = [\xi_{0i}, \ldots, \xi_{\mathcal{K}i}]$.
end for
Train a logistic regression classifier using $\vec{\xi}_i$ as inputs and class labels as targets.
Testing:
for each test sample $i = 1$ to W_{test} in both classes **do**
  Generate intensity distribution as in the training stage.
  Simulate S detection events to obtain empirical probabilities $\hat{P}_i(u_j)$.
  Compute empirical eigentask vector $\hat{\vec{\xi}}_i$.
  Classify $\hat{\vec{\xi}}_i$ using the trained classifier.
end for
Repeat testing for multiple independent sets of test samples.
Compute mean and range of success probability P_{succ} as a function of $\mathcal{K}$ and S.

A. Simulation for imaging a source outside the Rayleigh limit

We consider a class of distinguishing tasks using direct imaging. This serves as an additional simulation example not included in the main text; the simulation presented in the main text is detailed in the next subsection. The pseudocode for this simulation is provided in Algorithm 1 and is further explained below. The spatial intensity distribution $I(u)$ is defined over the interval $[-L/2, L/2]$ and takes binary values, $I(u) \in \{0, i_0\}$, where i_0 is fixed by the normalization condition $\int du\, I(u) = 1$, resembling the structure of a QR code. The density matrix of the state on the imaging plane is given in Eq. 1, which is reproduced below for convenience

$$\rho = \int du du_1 du_2 I(u) \psi(u - u_1) |u_1\rangle \langle u_2| \psi(u - u_2), \tag{101}$$

where the PSF is chosen as $\psi(u) = \exp\!\big(-u^2/4\sigma^2\big)/(2\pi\sigma^2)^{1/4}$ for all simulations in this section, with $\sigma = 1$. We consider the imaging of a source beyond the Rayleigh limit, i.e., L can be greater than σ. For simplicity, the interval is discretized into N equally spaced pixels, with the intensity assumed to be constant within each pixel.

To generate random instances of $I(u)$, we introduce an indicator function $\eta(u) \in \{0,1\}$ defined over $[-L/2, L/2]$, one example is shown in the Supplementary Figure 19(a). The function $\eta(u)$ specifies which spatial regions are "active" (where $\eta(u) = 1$) and which are "inactive" (where $\eta(u) = 0$). The generation of $I(u)$ differs depending on the following two cases:

Case 1 (active case): Intensity is allowed to appear only in the regions where $\eta(u) = 1$. That is, for each pixel whose centroid lies in a region where $\eta(u) = 1$, the corresponding value of $I(u)$ is independently set to 1 with activation

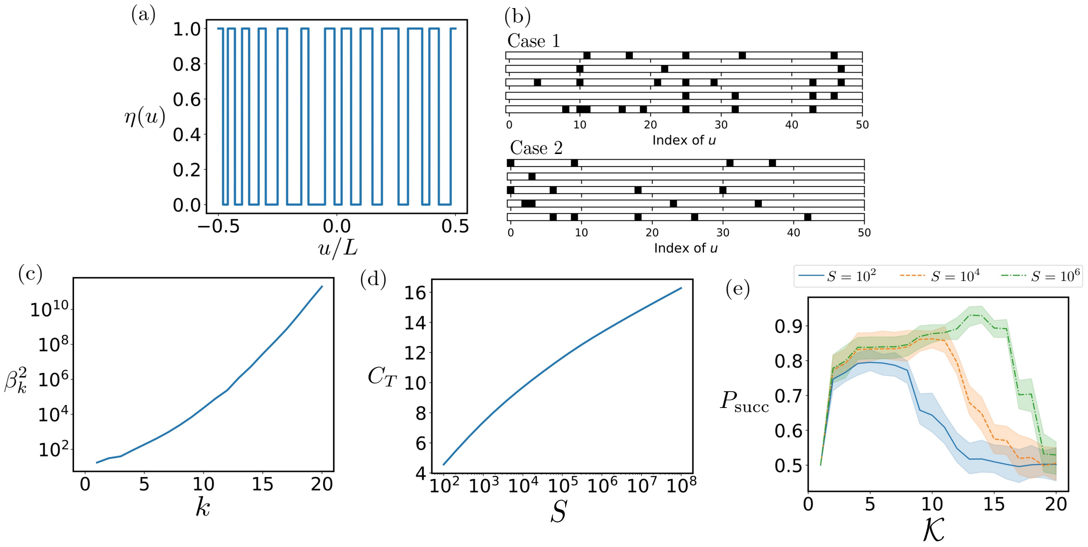

Supplementary Figure 19. (a) Indicator function $\eta(u)$ used for the simulation here. (b) Examples of intensity distributions randomly generated in the two cases; for demonstration purposes, we choose the number of pixels $N = 50$. We set the probability of $I(u) = 1$ to be $p_0 = 0.2$ for each pixel where $\eta(u) = 1$. (c) The eigenvalues β_k^2 for different orders k. In the calculation, we use $N = 200$ pixels and set $L = 10$. A total of $2W_{\rm train} = 400$ different intensity distributions are generated to compute the prior distribution and the corresponding g and d. (d) The total REC C_T as a function of the sample number S. (e) The success probability $P_{\rm succ}$ for different sample sizes S as a function of the highest order $\mathcal{K}$ of the eigentask included in the training and testing procedure. We use a test set of $2W_{\rm test} = 600$ different intensity distributions, with $W_{\rm test} = 300$ distributions for each case, to calculate the success probability of distinguishing between the two cases. This calculation is repeated 50 times using $2W_{\rm test} = 600$ independently generated intensity distributions each time. The line in the figure represents the mean success probability, and the shaded region indicates the maximum and minimum values across the 50 repetitions.

probability p_0, and 0 otherwise. For pixels where $\eta(u) = 0$, we set $I(u) = 0$ deterministically. This simulates a sparse intensity pattern supported only within the $\eta(u) = 1$ region.

Case 2 (inactive case): Intensity is allowed only in the complementary region where $\eta(u) = 0$. For each pixel with $\eta(u) = 0$ at its centroid, $I(u)$ is randomly set to 1 with probability p_0, and 0 otherwise. For pixels where $\eta(u) = 1$, we set $I(u) = 0$. This case represents an alternative intensity pattern that avoids the $\eta(u) = 1$ regions.

We provide example intensity distributions generated in the two cases in Supplementary Figure 19(b). The goal of the distinguishing task is to determine, based on the observed data on the image plane, whether a given realization of $I(u)$ was generated according to Case 1 or Case 2.

We first generate a training set of $2W_{\rm train}$ intensity distributions according to the formalism described above, with $W_{\rm train}$ samples for each case. The corresponding theoretical measurement outcomes are computed without sample noise. To model the task as a learning problem [30], we incorporate prior knowledge by treating the intensity values at each pixel as the input parameters for each distribution. Using these inputs, we construct the matrices d_k and g_{ij} by averaging over the all the $2W_{\rm train}$ intensity distributions in the training set. This enables us to compute the eigentasks and their associated eigenvalues β_k^2.

Once the eigenvectors r_k, as defined in Eq. 27, are obtained with the prior quantified by d_i and g_{ij}, and the pixel-wise detection probability distribution $P_m(u_j)$ for the mth intensity distribution is known theoretically, we construct the kth eigentask as $\xi_{km} = \sum_j r_{kj} P_m(u_j)$. This yields, for each intensity distribution indexed by m, an eigentask vector $\vec{\xi}_m = [\xi_{0m}, \xi_{1m}, \xi_{2m}, \ldots, \xi_{\mathcal{K}m}]$. We truncate the eigentasks at order $\mathcal{K}$, which is determined based on the number of detection events S—a choice we will justify later. For each of the two classes, we generate $W_{\rm train}$ such eigentask vectors, forming two datasets that will be used as training inputs for the logistic regression classifier.

We use logistic regression, implemented via the standard `LogisticRegression` class from the widely used Python package `scikit-learn`, to perform binary classification. Logistic regression models the probability that a sample belongs to class 1 as $P(y = 1 \mid \vec{x}) = 1/(1 + \exp(-\vec{w}^T \vec{x}))$, where $\vec{x}$ is the input feature vector and $\vec{w}$ is the weight vector learned during training. A classification decision is made by thresholding this probability at 0.5. In our case, the input features $\vec{x}$ correspond to the eigentask vector $\vec{\xi}_m$, with each component normalized by dividing by its mean absolute value across the training set before being passed to the classifier. Since we know which class each eigentask vector $\vec{\xi}_m$ corresponds to during training, we use this standard python package to fit the weight vector $\vec{w}$ accordingly.

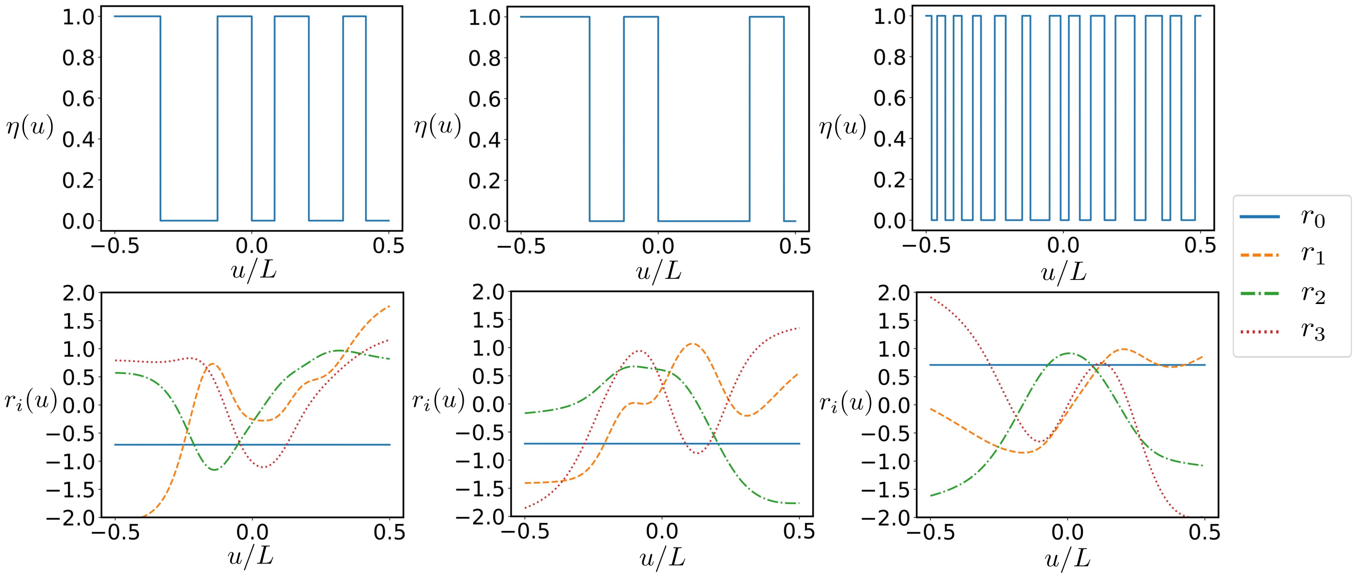

Supplementary Figure 20. Different indicator functions $\eta(u)$ lead to different eigentasks r_k, which are obtained by solving the generalized eigenvalue equation $Gr_k = \lambda_k Dr_k$ in Eq. 27. In this example, the direct imaging measurement yields the probability distribution of detecting a photon at each position u. The kth eigentask is given by $\sum_i r_k(u_i)P(u_i)$, where $P(u_i)$ is the probability of detecting a photon at position u_i. $L = 10$, $\sigma = 1$.

During testing, we again generate intensity distributions from the two classes. For each intensity distribution, we fix the total number of detected photons (sample number) to be S. By counting the number of photons detected in each pixel on the image plane, we obtain an empirical probability distribution $\hat{P}_m(u_j)$ for the mth intensity distribution. Using this distribution, we compute the corresponding empirical eigentask vector $\hat{\vec{\xi}}_m = [\hat{\xi}_{0m}, \hat{\xi}_{1m}, \hat{\xi}_{2m}, \ldots, \hat{\xi}_{\mathcal{K}m}]$. The choice of truncation order $\mathcal{K}$ depends on the precision with which each eigentask can be estimated. The eigenvalues β_k^2 characterize the sample size threshold required for accurately estimating each eigentask component; accurate estimation becomes possible once $S \gg \beta_k^2$. In Supplementary Figure 19(c), we show the eigenvalues β_k^2 for the model considered here. A suitable choice of $\mathcal{K}$ satisfies $\beta_{\mathcal{K}}^2 \sim S$, ensuring that all retained eigentask components can be estimated reliably. The resulting eigentask vectors $\hat{\vec{\xi}}_m$ are then used for classification using the trained logistic regression model.

As in Supplementary Figure 19(d), the success probability of distinguishing the two cases is plotted as a function of the number of included eigentasks $\mathcal{K}$ for various sample sizes S. As observed, the success probability initially increases with $\mathcal{K}$ and then decreases. This behavior is intuitive: increasing $\mathcal{K}$ allows more eigentasks to be included, capturing more information about the intensity distribution and thus improving classification performance. However, beyond a certain point, the higher-order eigentasks are poorly estimated due to limited sample size S. Naively including these noisy components degrades the classifier's performance and ultimately reduces the success probability. As shown in Supplementary Figure 19(c), when $S = 10^2$, 10^4, and 10^6, we can roughly reliably estimate approximately the first 5, 10, and 14 eigentasks, respectively. As expected, Supplementary Figure 19(d) shows that when we naively include the first $\mathcal{K}$ eigentasks, the success probability P_{succ} peaks at around $\mathcal{K} = 5$, 10, and 14, respectively.

Supplementary Figure 20 shows that the prior distribution can significantly influence the form of the eigentasks, which in general do not have a simple form.

B. Imaging multiple compact sources

Algorithm 2 Distinguishing multiple compact sources using eigentasks

Input: Number of pixels N, total region size $\mathbb{L}$, activation probability p_0, number of compact sources Q, number of training samples per class W_{train}, number of test samples per class W_{test}, total detected photons per test image S, truncation order $\mathcal{K}$
Define the position function $\zeta(u) \in \{0,1\}$ to specify the spatial locations of the Q compact sources.
For each compact source S_ℓ ($\ell = 1, \ldots, Q$), define an indicator function $\eta_\ell(u) \in \{0,1\}$ specifying the active region within that source.
Training:
for each class (Case 1: active; Case 2: inactive) **do**
  for $i = 1$ to W_{train} **do**
    Initialize $I_i(u_j) = 0$ for all pixels j.
    for each compact source $\ell = 1, \ldots, Q$ **do**
      for each pixel j where $\zeta(u_j) = 1$ (i.e. within a compact source) **do**
        if Case 1 and $\eta_\ell(u_j) = 1$ **then**
          Set $I_i(u_j) = 1$ with probability p_0, else 0.
        else if Case 2 and $\eta_\ell(u_j) = 0$ **then**
          Set $I_i(u_j) = 1$ with probability p_0, else 0.
        end if
      end for
    end for
    Normalize $I_i(u)$ so that $\sum_{j=1}^{N} I_i(u_j) = 1$, where each nonzero $I_i(u_j)$ takes value i_0.
  end for
end for
Compute prior matrices d_k and g_{ij} from all training samples, taking the prior to be the uniform distribution over the training set.
Solve the eigenvalue problem (Eq. 14) to obtain eigenvectors r_k and eigenvalues β_k^2.
for each training sample i in both classes **do**
  Compute theoretical detection probabilities $P_i(j)$ for the chosen measurement method (direct imaging, separate SPADE, orthogonalized SPADE).
  Compute eigentask components $\xi_{k,i} = \sum_j r_{kj} P_i(j)$.
  Form the truncated eigentask vector $\vec{\xi_i} = [\xi_{0i}, \ldots, \xi_{\mathcal{K}i}]$.
end for
Train a logistic regression classifier using $\vec{\xi_i}$ as inputs and the class labels as targets.
Testing:
for each test sample $i = 1$ to W_{test} in both classes **do**
  Generate a new intensity distribution $I_i(u)$ as in the training phase.
  Simulate S detection events to obtain empirical probabilities $\hat{P}_i(j)$.
  Compute empirical eigentask vector $\hat{\vec{\xi_i}} = [\hat{\xi}_{0i}, \ldots, \hat{\xi}_{\mathcal{K}i}]$.
  Classify $\hat{\vec{\xi_i}}$ using the trained logistic regression model.
end for
Repeat the testing process for multiple independent sets of test samples.
Compute the mean and range of the success probability P_{succ} as a function of $\mathcal{K}$ and S.

We now consider a similar task: distinguishing multiple compact sources using the direct imaging method, the separate SPADE method, and the orthogonalized SPADE method. This corresponds to the simulation example presented in the main text. The pseudocode for this simulation is provided in Algorithm 2 and is further explained below.

As before, we randomly generate images according to two different cases specified by the indicator function $\eta(u)$. In this setting of imaging multiple compact sources, we assume that the intensity is nonzero only at the locations of a few compact sources where the position function $\zeta(u) = 1$, with sizes within the Rayleigh limit, i.e., $\alpha \ll 1$. As illustrated in Supplementary Figure 21, we present an example with four compact sources defined by $\zeta(u)$. For each compact source, the corresponding indicator function $\eta(u)$ within its region is shown, along with several examples of the randomly generated intensity distributions for both cases and for each source.

As in the previous simulation of imaging a general source, we use $2W_{\text{train}}$ different intensity distributions to generate the corresponding g_{ij} and d_i. This allows us to compute the eigentasks and the associated eigenvalues β_k^2. Once the eigenvectors r_k are obtained, and the theoretical probability distribution $P_m(j)$ — corresponding to the mth intensity distribution and the jth outcome — is known, we construct the kth eigentask as $\xi_{km} = \sum_j r_{kj} P_m(j)$. Note that, for

the direct imaging method, $P_m(j)$ represents the probability of detecting photons at position u_j. For the separate SPADE method, the outcomes are

$$\begin{aligned}&\frac{1}{2Q}\left|b_{1,0}\right\rangle\left\langle b_{1,0}\right|,\cdots,\frac{1}{2Q}\left|b_{4,0}\right\rangle\left\langle b_{4,0}\right|,\frac{1}{2Q}\left|\phi_{1,0,\pm}\right\rangle\left\langle\phi_{1,0,\pm}\right|,\cdots,\frac{1}{2Q}\left|\phi_{4,0,\pm}\right\rangle\left\langle\phi_{4,0,\pm}\right|,\\&\frac{1}{2Q}\left|\phi_{1,1,\pm}\right\rangle\left\langle\phi_{1,1,\pm}\right|,\cdots,\frac{1}{2Q}\left|\phi_{4,1,\pm}\right\rangle\left\langle\phi_{4,1,\pm}\right|,\frac{1}{2Q}\left|\phi_{1,2,\pm}\right\rangle\left\langle\phi_{1,2,\pm}\right|,\cdots,\frac{1}{2Q}\left|\phi_{4,2,\pm}\right\rangle\left\langle\phi_{4,2,\pm}\right|,\cdots\end{aligned}\tag{102}$$

which are defined in Eq. 79. And the orthogonalized SPADE method has outcomes in the following order

$$\begin{aligned}&\frac{1}{2}\left|b_1^{(0)}\right\rangle\left\langle b_1^{(0)}\right|,\cdots,\frac{1}{2}\left|b_4^{(0)}\right\rangle\left\langle b_4^{(0)}\right|,\frac{1}{2}\left|\phi_{1,\pm}^{(0)}\right\rangle\left\langle\phi_{1,\pm}^{(0)}\right|,\cdots,\frac{1}{2}\left|\phi_{4,\pm}^{(0)}\right\rangle\left\langle\phi_{4,\pm}^{(0)}\right|,\\&\frac{1}{2}\left|\phi_{1,\pm}^{(1)}\right\rangle\left\langle\phi_{1,\pm}^{(1)}\right|,\cdots,\frac{1}{2}\left|\phi_{4,\pm}^{(1)}\right\rangle\left\langle\phi_{4,\pm}^{(1)}\right|,\frac{1}{2}\left|\phi_{1,\pm}^{(2)}\right\rangle\left\langle\phi_{1,\pm}^{(2)}\right|,\cdots,\frac{1}{2}\left|\phi_{4,\pm}^{(2)}\right\rangle\left\langle\phi_{4,\pm}^{(2)}\right|,\cdots\end{aligned}\tag{103}$$

which are defined in Eq. 81. This yields, for each intensity distribution indexed by m, an eigentask vector $\vec{\xi}_m = [\xi_{0m}, \xi_{1m}, \xi_{2m}, \ldots, \xi_{\mathcal{K}m}]$. We truncate the eigentasks at order $\mathcal{K}$, which is determined by the number of detection events S—a choice we will justify later. For each of the two classes, we get W_{train} such eigentask vectors, forming two datasets that serve as training inputs for the logistic regression classifier.

During testing, we generate $2W_{\text{test}}$ intensity distributions from the two cases. For each intensity distribution, we fix the total number of detected photons (sample number) to be S. By counting the number of occurrences of each outcome, we obtain an empirical probability distribution $\hat{P}_m(j)$ for the mth intensity distribution. Using this distribution, we compute the corresponding empirical eigentask vector $\hat{\vec{\xi}}_m = [\hat{\xi}_{0m}, \hat{\xi}_{1m}, \hat{\xi}_{2m}, \ldots, \hat{\xi}_{\mathcal{K}m}]$. The resulting eigentask vectors $\hat{\vec{\xi}}_m$ are then used as input for classification with the trained logistic regression model.

We then show the performance of distinguishing the two cases using the three approaches in Supplementary Figure 22. In this calculation, we use the position function $\zeta(u)$ and the indicator function $\eta(u)$, as illustrated in Supplementary Figure 21, to generate the intensity distributions for the two cases. We observe that the success probability P_{succ} first increases with $\mathcal{K}$ and then decreases to around 50% in Supplementary Figure 22(d1)-(d3). This behavior is expected because we should only include the eigentasks that can be reliably estimated given the sample number S.

Comparing the success probability P_{succ} across the three approaches, since the distance between the compact sources is not much larger than the width of the PSF σ, we expect the separate SPADE method to perform comparably to the direct imaging case, while our orthogonalized SPADE method should achieve better performance. This is explicitly demonstrated in Supplementary Figure 22(d1)-(d3), where the peak success probability P_{succ} for the orthogonalized SPADE method is clearly higher than that of the other two approaches when $S = 10^{10}$.

This simulation also illustrates the operational meaning of the total REC C_T: it roughly indicates the number of eigentasks that should be included in the fitting. This is because β_k^2 serves as an approximate threshold on the sample number required to reliably estimate the kth eigentask, and $C_T = \sum_k 1/(1+\beta_k^2/S)$, where each term $1/(1+\beta_k^2/S)$ approaches 1 when $S \gg \beta_k^2$. Hence, C_T roughly counts how many eigentasks can be reliably estimated. When the number of samples is $S = 10^4$ or $S = 10^7$, the total REC C_T is roughly the same across the three approaches, and we observe similar behavior of P_{succ} as a function of $\mathcal{K}$. However, at $S = 10^{10}$, the total REC C_T for the orthogonalized SPADE method becomes noticeably larger than that of the other two approaches, reaching roughly 13. This aligns precisely with the peak position in the P_{succ} plot for the orthogonalized SPADE method. In comparison, for direct imaging and the separate SPADE method, C_T is about 10, which is also consistent with the peak positions in their respective P_{succ} plots. These observations clearly demonstrate the operational meaning of C_T, which reflects the number of eigentasks that should be included in the downstream analysis. Being able to include more reliably estimated features can improve the performance of the subsequent distinguishing task, whereas including eigentasks that are not well estimated can degrade the performance. A larger C_T indicates that more eigentasks can be included in the downstream analysis, reflecting a better-performing imaging method.

The eigentasks here exhibit complex structures that depend on the positions of the compact sources $\zeta(u)$ and the indicator function $\eta(u)$. The first four eigenvectors for the orthogonalized SPADE method are shown in Supplementary Figure 22(c). This demonstrates the strength of the quantum learning approach in identifying which eigentasks should be included and determining up to which order they should be incorporated in the logistic regression for downstream analysis. From Supplementary Figure 22(a), we observe that the eigenvalues increase almost smoothly across all approaches considered here, rather than showing the stepwise increase we previously described. This is because the value of $\alpha = 0.1$ in Supplementary Figure 22; if α were even smaller, the β_k^2 would be dominated by the scaling over α. These aspects — the eigentask structure and the eigenvalue behavior — are discussed in detail in Supplementary Note 5 E.

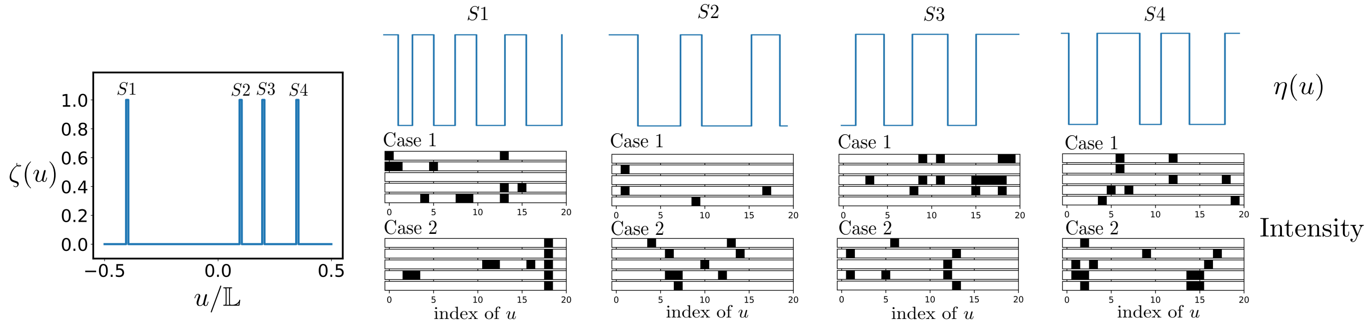

Supplementary Figure 21. The position function $\zeta(u)$ specifies the positions of four compact sources, labeled $S1$, $S2$, $S3$, and $S4$. For each compact source, the intensity distribution is randomly generated based on another indicator function $\eta(u)$. In case 1, the intensity is nonzero only where $\eta(u) = 1$, whereas in case 2, it is nonzero only where $\eta(u) = 0$. All four compact sources are confined within the interval $[-\mathbb{L}/2, \mathbb{L}/2]$ with $\mathbb{L} = 10$, and each source has a size of 0.1, which is smaller than the PSF width $\sigma = 1$. The intensity distributions are randomly generated, and we present five examples of both cases for each compact source above. Note that the intensity of each pixel is assigned based on the position of its centroid, so edge pixels may appear slightly misaligned with the indicator function $\eta(u)$; however, this effect becomes negligible as the number of pixels increases.

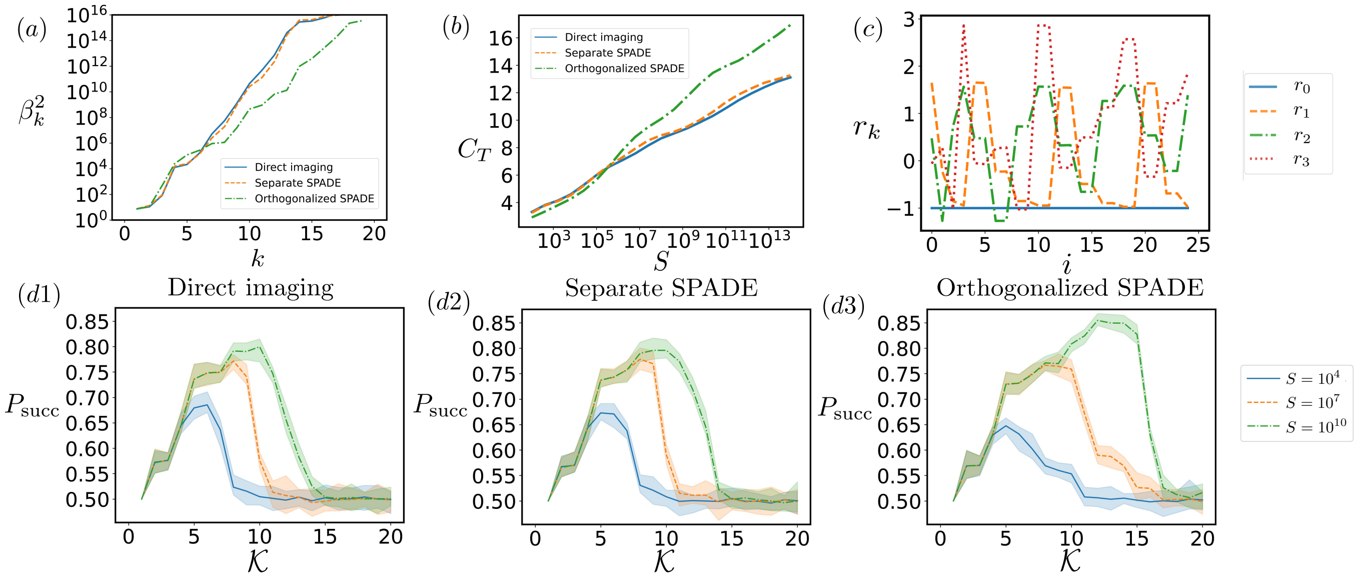

Supplementary Figure 22. (a) The eigenvalues β_k^2 for different orders k obtained using the three approaches to image four compact sources. (b) The total REC C_T as a function of the sample number S for the three approaches. (c) The first four eigenvectors r_k for the orthogonalized SPADE method, where i labels the ith outcome. (d1)–(d3) The success probability P_{succ} as a function of the highest order $\mathcal{K}$ of the eigentasks included in the training and testing procedures for classifying the images in the two cases, evaluated at different sample sizes S. The solid line represents the mean success probability, and the shaded region shows the maximum and minimum values across the 20 repetitions. We set $\alpha = 0.1$ in this figure.

In the calculation shown in Supplementary Figure 22, we use $N_{\max} = 20$ pixels for each compact source. A total of $2W_{\text{train}} = 400$ intensity distributions — with $W_{\text{train}} = 200$ per case — are generated to compute the prior distribution and the corresponding g and d for the eigenvalues β_k^2 and the total REC C_T. These $2W_{\text{train}} = 400$ intensity distributions, together with the corresponding theoretical eigentasks, are also used as the training data for logistic regression. A test set of $2W_{\text{test}} = 2000$ intensity distributions — with $W_{\text{test}} = 1000$ per case — is used to evaluate the success probability of distinguishing the two cases. This calculation is repeated 20 times, each with an independently generated test set of $2W_{\text{test}} = 2000$ intensity distributions.

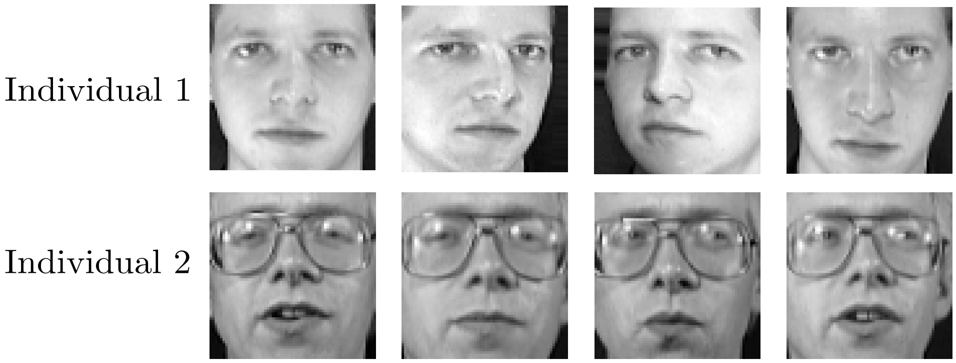

Supplementary Figure 23. Example images of two individuals from the Olivetti Faces dataset. Each row shows several images belonging to the same individual, illustrating natural variations in expression, pose, and illumination. These samples form the input set for the face-recognition task considered in this work.

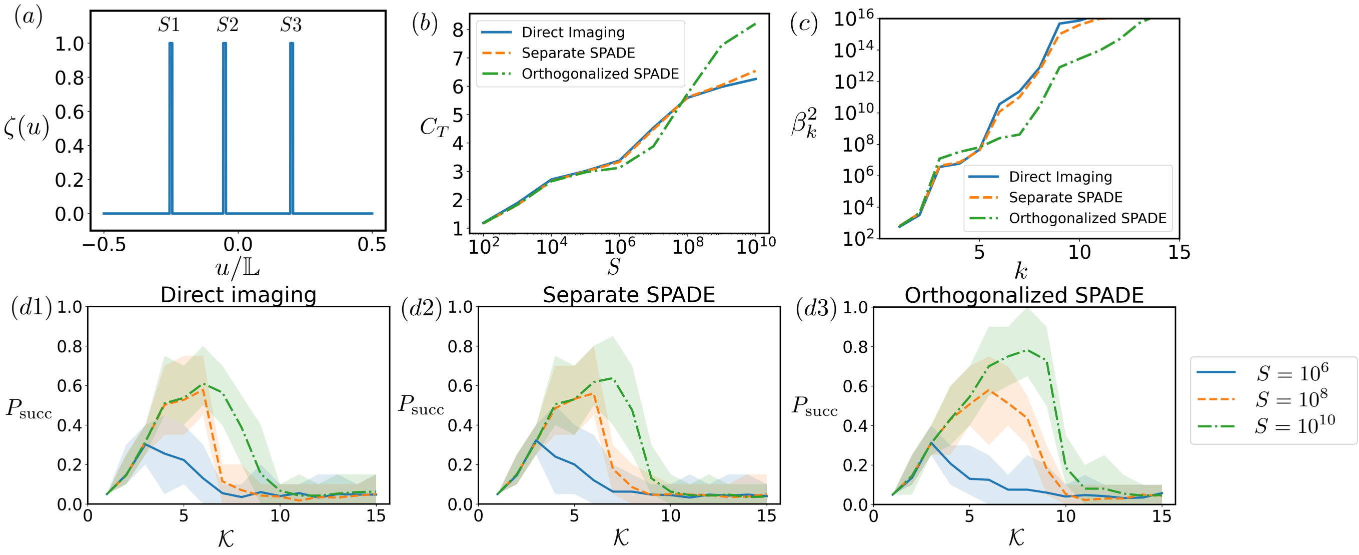

Supplementary Figure 24. Simulation for face recognition. (a) The position function $\zeta(u)$ specifies the positions of three compact sources, labeled $S1$, $S2$, and $S3$. All three compact sources are confined within the interval $[-\mathbb{L}/2, \mathbb{L}/2]$ with $\mathbb{L} = 10$, and each source has a size of 0.1, which is smaller than the PSF width $\sigma = 1$. (b) The eigenvalues β_k^2 for different orders k obtained using the three approaches. (c) The total REC C_T as a function of the sample number S for the three approaches. (d1)–(d3) The success probability P_{succ} as a function of the highest order $\mathcal{K}$ of the eigentasks included in the training and testing procedures for face recognition, evaluated at different sample sizes S. The lines represent the mean success probability, and the shaded region shows the maximum and minimum values across the 20 repetitions. We set $\alpha = 0.1$ in this figure.

C. Face recognition

Algorithm 3 Face-recognition simulation using eigentasks

Input: Number of individuals N_{person}, training images per person w_{train}, test images per person w_{test}, number of compact sources M, total region size $\mathbb{L}$, PSF width σ, total detected photons per test image S, truncation order $\mathcal{K}$, measurement type (direct imaging, separate SPADE, orthogonalized SPADE)

Preprocessing:

for each face image **do**
  Load the 64×64 grayscale face image.
  Vectorize the image into a 1D array of $N_{\mathrm{pix}} = 4096$ pixels.
  Split the array into M consecutive segments of equal length.
  Assign segment m to compact source S_m using the position function $\zeta(u)$.
  Construct the 1D intensity distribution $I(u)$ and normalize it so $\sum_j I(u_j) = 1$.
end for

Training:

Randomly select w_{train} training images for each of the N_{person} individuals.
for each training image i **do**
  Construct $I_i(u)$ following the preprocessing steps.
end for
Compute prior matrices d_k and g_{ij} from all training intensity distributions, taking the prior to be uniform.
Solve the eigenvalue problem (Eq. 14) to obtain eigenvectors r_k and eigenvalues β_k^2.
for each training image i **do**
  Compute theoretical detection probabilities $P_i(j)$ for the chosen measurement method.
  Compute eigentask components $\xi_{k,i} = \sum_j r_{kj} P_i(j)$ for $k = 0, \ldots, \mathcal{K}$.
  Form the truncated eigentask vector $\vec{\xi_i} = [\xi_{0i}, \ldots, \xi_{\mathcal{K}i}]$.
  Assign the class label corresponding to the identity of the person.
end for
Train a logistic regression classifier using the eigentask vectors $\vec{\xi_i}$ as inputs and person identities as labels.

Testing:

Select w_{test} test images per person.
for each test image i **do**
  Construct a new 1D intensity distribution $I_i(u)$ as in the training phase.
  Simulate S detection events based on $I_i(u)$ and the chosen measurement to obtain empirical probabilities $\hat{P}_i(j)$.
  Compute empirical eigentask components $\hat{\xi}_{k,i} = \sum_j r_{kj} \hat{P}_i(j)$.
  Form the empirical eigentask vector $\hat{\vec{\xi_i}} = [\hat{\xi}_{0i}, \ldots, \hat{\xi}_{\mathcal{K}i}]$.
  Classify $\hat{\vec{\xi_i}}$ using the trained logistic regression model.
end for
Repeat the testing process using randomly selected training and test sets.
Compute the mean and range of the success probability P_{succ} as functions of $\mathcal{K}$ and S.

In this subsection, we present a simulation example of face recognition in the context of imaging below the Rayleigh limit, using all three measurement strategies: the direct imaging method, the separate SPADE method, and the orthogonalized SPADE method. The pseudocode for this simulation is provided in Algorithm 3 and is further explained below.

As shown in Supplementary Figure 23, the face images used in our simulation are taken from the Olivetti Faces dataset provided by AT&T Laboratories Cambridge and distributed through `scikit-learn`. Each individual has ten 64×64 grayscale images with different facial expressions and small variations in pose. We use these images as the training and testing data for the face-recognition task considered in this simulation. Our theoretical analysis is developed for one-dimensional intensity distribution, whereas the face images are inherently two-dimensional. While it should be possible to extend our framework to the fully two-dimensional setting, we leave this to future work, as most superresolution techniques generalize naturally to higher dimensions without introducing additional qualitative differences [4, 43–45]. Reducing a two-dimensional image to a one-dimensional signal through rasterization is also a standard dimensionality-reduction technique in machine learning and image processing. To embed the data into our one-dimensional model, each 64×64 face image is converted into a one-dimensional signal by rasterizing the image: all pixel rows are concatenated sequentially to form a vector of length 4096. We partition this vector into $M = 3$ segments. These segments play the role of compact sources and are placed at the positions specified by the position function $\zeta(u)$, as illustrated in Supplementary Figure 23(a). In this way, each two-dimensional face image is embedded into the one-dimensional framework required for our superresolution analysis.

We use $w_{\text{train}} = 9$ training images per person and consider a total of $N_{\text{person}} = 20$ individuals. As in the simulation of the previous subsections, this gives $W_{\text{train}} = w_{\text{train}} N_{\text{person}} = 180$ training face images corresponding to different people. These training data are used to generate the quantities g_{ij} and d_i, which in turn allow us to compute the eigentasks and the associated eigenvalues β_k^2. Once the eigenvectors r_k are obtained, and the theoretical probability distribution $P_m(j)$—corresponding to the mth intensity distribution and the jth outcome—is known, we construct the kth eigentask as $\xi_{km} = \sum_j r_{kj} P_m(j)$. For the direct imaging method, $P_m(j)$ represents the probability of detecting photons at position u_j. For the separate SPADE method, the outcomes are given in Eq. 102. The orthogonalized SPADE method uses the outcomes in the order specified in Eq. 103. This procedure yields, for each intensity distribution indexed by m, an eigentask vector $\vec{\xi}_m = [\xi_{0m}, \xi_{1m}, \xi_{2m}, \ldots, \xi_{\mathcal{K}m}]$. We truncate the eigentasks at order $\mathcal{K}$, which is set by the number of detection events S—a choice we will justify later. For each individual, we obtain w_{train} such eigentask vectors, forming datasets that serve as the training inputs for the logistic regression classifier.

During testing, we use $w_{\text{test}} = 1$ image per person and consider the same $N_{\text{person}} = 20$ individuals, giving a total of $W_{\text{test}} = w_{\text{test}} N_{\text{person}} = 20$ test face images corresponding to different people. For each intensity distribution, we fix the total number of detected photons (sample number) to be S. By counting the occurrences of each outcome, we obtain an empirical probability distribution $\hat{P}_m(j)$ for the mth intensity distribution. Using this distribution, we then compute the corresponding empirical eigentask vector $\hat{\vec{\xi}}_m = [\hat{\xi}_{0m}, \hat{\xi}_{1m}, \hat{\xi}_{2m}, \ldots, \hat{\xi}_{\mathcal{K}m}]$, which is also truncated at order $\mathcal{K}$. These empirical eigentask vectors $\hat{\vec{\xi}}_m$ are used as inputs to the trained logistic regression classifier.

We then evaluate the performance of identifying these $N_{\text{person}} = 20$ individuals in the context of imaging below the Rayleigh limit using the three approaches, as shown in Supplementary Figure 24. We observe that the success probability P_{succ} first increases with $\mathcal{K}$ and then decreases, as illustrated in Supplementary Figure 24(d1)–(d3). This trend is expected: only eigentasks that can be reliably estimated given the sample number S should be included. Comparing the success probability P_{succ} across the three approaches, since the distance between the compact sources is not much larger than the width of the PSF σ, we expect the separate SPADE method to perform comparably to the direct imaging case, while our orthogonalized SPADE method should achieve better performance. This is explicitly demonstrated in Supplementary Figure 24(d1)-(d3), where the peak success probability P_{succ} for the orthogonalized SPADE method is clearly higher than that of the other two approaches when $S = 10^{10}$.

This simulation also illustrates the operational meaning of the total REC C_T: it approximately indicates how many eigentasks should be included in the fitting. The reason is that β_k^2 serves as a threshold on the sample number required to reliably estimate the kth eigentask, and $C_T = \sum_k 1/(1+\beta_k^2/S)$, where each term $1/(1+\beta_k^2/S)$ approaches 1 when $S \gg \beta_k^2$. Thus, C_T effectively counts the number of eigentasks that can be reliably estimated. From Supplementary Figure 24(c), we observe that the eigenvalues β_k^2 for the orthogonalized SPADE method are significantly smaller than those of the other two methods for approximately $k \geq 6$. When the number of samples is $S = 10^6$ or $S = 10^8$, the total REC C_T is roughly the same across all three approaches, and the resulting dependence of P_{succ} on $\mathcal{K}$ is correspondingly similar. However, at $S = 10^{10}$, the total REC C_T for the orthogonalized SPADE method increases noticeably, reaching a value of roughly 8, while the corresponding values for direct imaging and separate SPADE remain around 6. This behavior matches the peak positions of P_{succ} in Supplementary Figure 24(d1)–(d3): the orthogonalized SPADE method peaks near $\mathcal{K} \approx 8$, while the other two methods peak near $\mathcal{K} \approx 6$. These observations clearly demonstrate the operational meaning of C_T: it reflects the number of eigentasks that should be included in the downstream analysis. Being able to incorporate more reliably estimated features improves the performance of the face recognition, whereas including poorly estimated eigentasks degrades it. A larger C_T therefore indicates that more eigentasks can be used effectively, corresponding to a better-performing imaging method.

In the calculation shown in Supplementary Figure 24(d1)–(d3), a total of $W_{\text{train}} = 180$ intensity distributions, with $w_{\text{train}} = 9$ per person, are generated to compute the prior distribution and the corresponding g_{ij} and d_i for determining the eigenvalues β_k^2 and the total REC C_T. These $W_{\text{train}} = 180$ intensity distributions, together with their theoretical eigentasks, are also used as the training data for logistic regression. A test set of $W_{\text{test}} = 20$ intensity distributions, with $w_{\text{test}} = 1$ per person, is used to evaluate the success probability of identifying each individual. This calculation is repeated 20 times. In each repetition, the $w_{\text{test}} + w_{\text{train}} = 10$ images available for each person are randomly split so that 9 images are used for training and 1 image is used for testing. Repeating the simulation with different random choices of test images prevents the result from depending on any particular image and provides a more reliable estimate of the overall performance.

D. Discussion

Here, we want to discuss the key advantages of applying the quantum learning formalism [30] to imaging problems, in comparison to traditional quantum parameter estimation approaches based on the Cramer-Rao bound. A central insight is that practical imaging tasks involve intrinsic ambiguities: (i) parameterization—it is unclear which

set of parameters best captures the task-relevant information, and different choices can lead to markedly different performance; (ii) finite-sample limitations—with limited data, only a subset of parameters can be reliably estimated, making it crucial to identify and retain well-estimated ones, which is especially challenging in the inherently infinite-dimensional setting of imaging. While quantum parameter estimation remains a powerful tool when the goal is to estimate well-defined parameters encoded in a system, this quantum learning approach is better suited for more general tasks that involve prior structure, model uncertainty, and finite sample limitations. We believe this approach opens the door to a range of practically important applications where traditional estimation strategies may fall short.

A core difficulty in analyzing imaging problems is that imaging inherently involve a large number of degrees of freedom. Conventional parameter estimation provides no clear guidance on which parameters to extract or how many to include in downstream analyses, such as logistic regression in our simulation. In this simulation, our objective is to distinguish between two cases, rather than to estimate a well-defined set of parameters. As such, it is unclear which quantities should be treated as parameters, and if so, up to what order. For example, one might consider the intensity values at each pixel as unknown parameters, or alternatively use the Fourier components or the moments of the intensity distribution—all of which, in principle, contain the full information and can be incorporated into the Fisher information framework. Furthermore, if moments are chosen as features, there remains ambiguity regarding how many moments to include. If we rely solely on Fisher information or traditional parameter estimation approaches, such questions must be addressed carefully on a case-by-case basis, lacking both conceptual simplicity and a unified theoretical foundation. In fact, we view this ambiguity as a significant drawback in earlier discussions of superresolution based on Fisher information [4–6, 43, 45–61], where a fixed set of parameters is assumed from the outset without a clear justification for their selection.

By contrast, the quantum learning framework identifies a natural basis—the eigentasks—derived from the measurement model and prior knowledge, which serve as meaningful and physically motivated features for downstream tasks such as logistic regression. While parameters such as pixel intensities, Fourier components, or moments can be chosen as inputs, the features that can actually be estimated should be determined by the measurement system itself, together with the known prior distribution over the inputs. These are precisely the eigentasks. Importantly, the total REC and the eigentasks are invariant under reparameterization. The eigentasks span the space of functions expressible as linear combinations of the measured features. If a subset of them can be estimated with sufficient accuracy, then any learnable function must lie within their span, making them a principled and well-justified choice for regression. Crucially, the number of detection events S determines how many eigentasks can be reliably estimated, and thus which features should be retained for classification. This is particularly important in superresolution problems, where the performance exhibits step-like behavior closely tied to the size of the sources. As a result, precision quantified solely by Fisher information is inadequate for guiding practical decisions—particularly when the prior is complex, the sample size is limited, or the task is not explicitly parameter estimation.

Supplementary Note 9. COMPARISON WITH THE CHERNOFF BOUND

Many machine-learning tasks can be viewed as discrimination problems. It is therefore natural to compare learning-based discrimination with the predictions obtained from more traditional statistical tools. In this section, we compare the learning approach with the likelihood-ratio method and with the corresponding Chernoff bounds.

Before going into details, we first emphasize that although many machine-learning tasks can be formulated as discrimination problems, this does not trivialize the learning-based approach. A key advantage of learning methods is their ability to handle highly complex image structures. In contrast, the likelihood-ratio method typically requires an accurate statistical model for the objects being imaged. For example, in a face-recognition task involving tens of individuals, it is essentially impossible to write down a closed-form likelihood function for the measured data that would allow an exact likelihood-ratio calculation. Learning-based approaches naturally excel in such high-dimensional, structured tasks, as is evident from their widespread use in everyday face-recognition systems, even though such tasks are, in principle, solvable by an optimal likelihood-ratio test. Our quantum learning method inherits these advantages while also being able to exploit the underlying structure of quantum measurements and quantum states, allowing it to operate effectively in the quantum-imaging setting considered here.

It is difficult to directly compare the likelihood-ratio method, the learning-based method, and the corresponding Chernoff bounds for the complex face-recognition example presented in the main text. We therefore begin by carrying out this comparison in a simpler and well-studied setting: distinguishing between a single point source and two point sources, a central problem in the theory of superresolution [58, 62–65]. We then use the insights gained from this simple example to discuss the behavior of the quantum learning–based method in the face-recognition task. For this elementary discrimination problem, previous works have predicted that for direct imaging, the error probability scales as $P_{\mathrm{err}} \sim \exp\bigl(-\alpha^4 S\bigr)$ up to constant prefactors, whereas the SPADE measurement achieves $P_{\mathrm{err}} \sim \exp\bigl(-\alpha^2 S\bigr)$, where S is the sample number. The error probability is simply related to the success probability by $P_{\mathrm{err}} = 1 - P_{\mathrm{succ}}$.

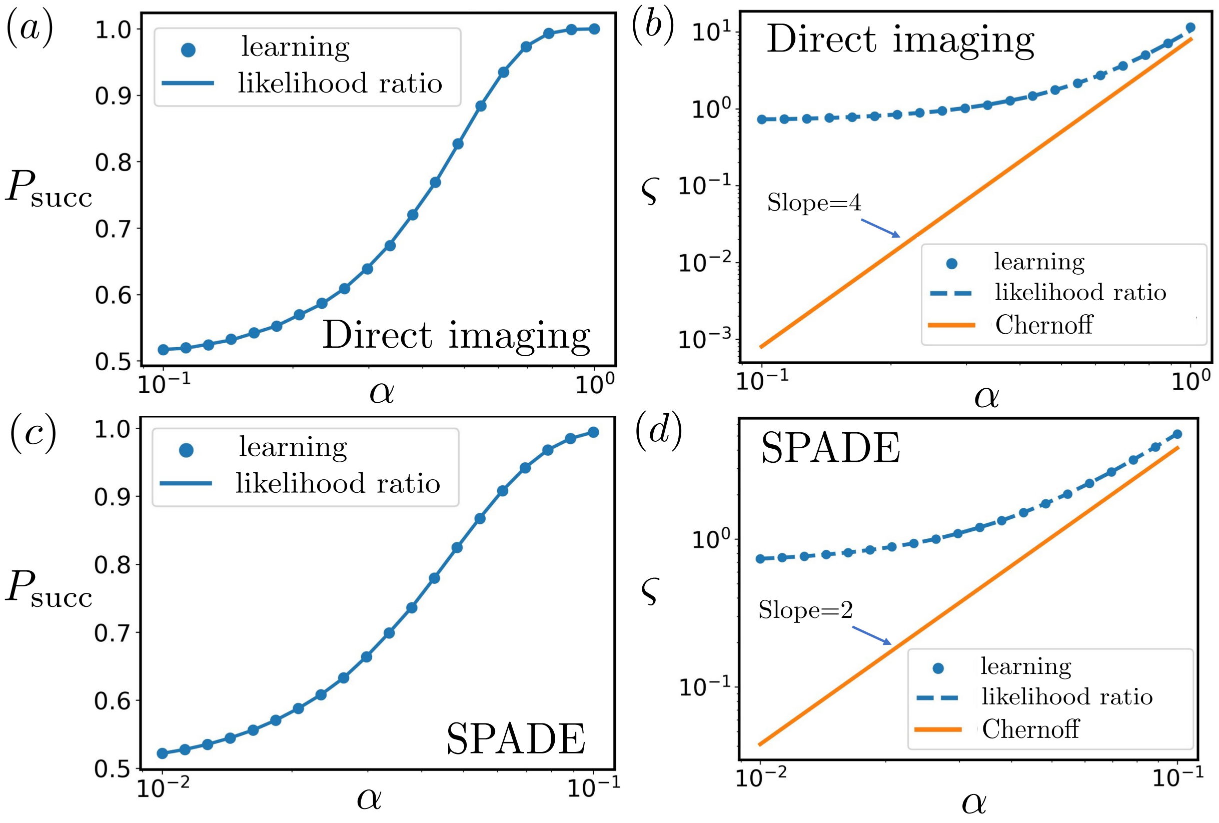

Supplementary Figure 25. Comparison of the learning approach, likelihood-ratio method, and Chernoff bound for discriminating a single point source from two point sources. (a), (b) Direct imaging with sample number $S = 10^7$. Panel (a) shows the success probability $P_{\rm succ}$ for the three approaches, and panel (b) shows the corresponding exponential index $\varsigma = -\log(1 - P_{\rm succ})$. (c), (d) SPADE measurements with sample number $S = 10^6$. Panel (c) shows the success probability $P_{\rm succ}$, and panel (d) shows the exponential index $\varsigma = -\log(1 - P_{\rm succ})$. The legend "learning" denotes the learning-based approach introduced in this work, "likelihood ratio" denotes the likelihood-ratio test, and "Chernoff" shows the theoretical Chernoff bound. In all panels, the single-source and two-source hypotheses occur with equal prior probability, and the two point sources have equal brightness with separation $\alpha d_{\rm sep}$, where $d_{\rm sep} = 0.6$ and the PSF width is $\sigma = 1$. α and ς are plotted in log scale, while $P_{\rm succ}$ is plotted in linear scale.

These scalings follow from the Chernoff bound [66–68], which upper-bounds the decay rate of the error probability via $P_{\rm err} \sim \exp(-SC)$ with $C = -\log\left(\min_{0\le t\le 1} \sum_i p_0(i)^t p_1(i)^{1-t}\right)$, where $p_{0,1}(i)$ denote the probabilities of obtaining outcome i under hypotheses 0 and 1, respectively. As shown in Supplementary Figure 25, our numerically calculated Chernoff bound exhibits the same scaling over α as reported in previous work [58, 62–65].

We further evaluate the performance of the likelihood ratio method, which compares how likely the observed data are under two competing hypotheses and selects the hypothesis with the higher likelihood. The likelihood ratio method decides between two hypotheses H_0 and H_1 by computing $\Lambda(\vec{x}) = P(\vec{x} \mid H_1)/P(\vec{x} \mid H_0)$, and choosing H_1 if $\Lambda(\vec{x}) > 1$, and H_0 otherwise, where $\vec{x}$ denotes the outcomes of S samples. To apply this method, we first construct the success-probability distributions for imaging a single point source and two point sources, corresponding to the two hypotheses in each measurement method, which yields explicit formulas for $P(\vec{x} \mid H_0)$ and $P(\vec{x} \mid H_1)$. Based on any observed sample outcome $\vec{x}$, we then compute $\Lambda(\vec{x})$ to make the prediction.

We also provide the performance of distinguishing the two hypotheses using the learning approach developed in our work in Supplementary Figure 25. In this calculation, we assume prior information that the single–point–source case and the two–point–source case occur with equal probability, which allows us to compute D, G, and the corresponding eigenvectors and eigenvalues based on Eq. 14. Following Supplementary Note 8, these eigenvectors are then used to construct the eigentasks $\xi_{ik} = \sum_j r_{kj} P_i(j)$, where $P_i(j)$ is the probability distribution for the jth outcome obtained from the ith test instance, which may correspond to either a single point source or two point sources. We use these truncated eigentask vectors $\vec{\xi}_i = [\xi_{0i}, \ldots, \xi_{\mathcal{K}i}]$ as the input features for training the logistic regression model. To avoid the instability caused by having very few real samples in this simple example, we employ model-based data

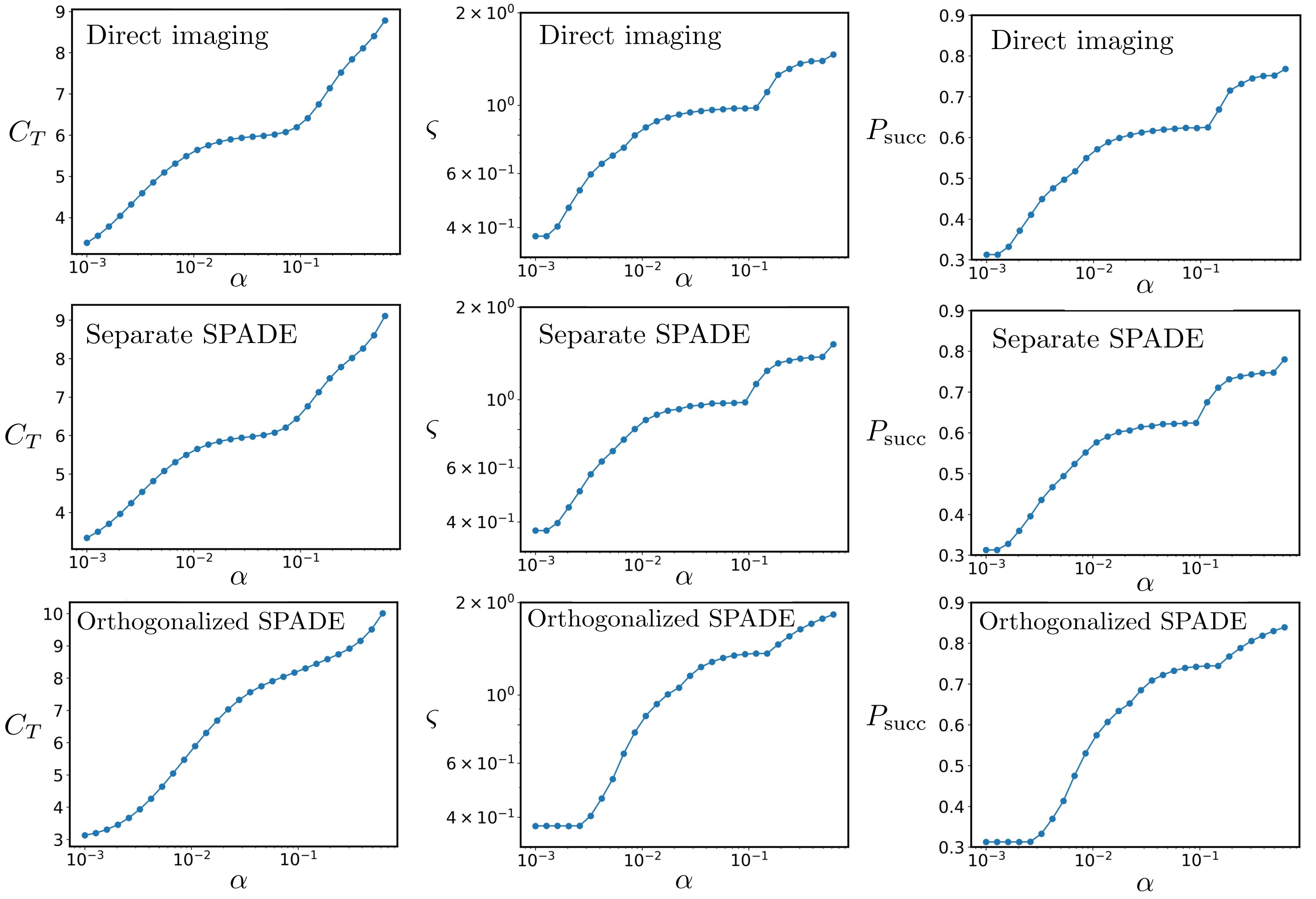

Supplementary Figure 26. Simulation of face-recognition performance as a function of the separation parameter α using the the learning-based approach, comparing three measurement strategies. The task involves identifying an individual from a set of 20 people, where each person has 9 training images and 1 additional image reserved for testing. Each face image is split into three compact pieces, consistent with the simulation setup of the previous section. All panels use $S = 10^{10}$ samples for each method. For every method, we report the total REC C_T, the success probability P_{succ}, and the corresponding exponential index $\varsigma = -\log(1 - P_{\text{succ}})$. The quantities P_{succ} and ς are obtained by running 100 repetitions and averaging the resulting success probabilities.

augmentation that generates synthetic eigentask features around each class's prototype model, yielding a sufficiently large and robust training set. Later, for each test instance, we sample the S outcomes to obtain the empirical probability distribution $\hat{P}_i(j)$ and compute the corresponding empirical eigentask $\hat{\xi}_{ik} = \sum_j r_{kj} \hat{P}_i(j)$, which is then used as the input to the trained classifier for evaluation. Supplementary Note 8 has shown that the success probability depends on the truncation order $\mathcal{K}$. Hence, we vary $\mathcal{K}$ to compute the corresponding success probability P_{succ} and select the peak value across all truncation orders. The plot reports the peak success probability achieved by this learning-based approach.

It is clear from Supplementary Figure 25 that the learning approach and the likelihood-ratio method perform similarly for this simple discrimination task. However, the likelihood ratio quickly becomes difficult to implement for practical problems such as face recognition, where the structure of the data is highly complex. In contrast, our learning-based approach naturally captures this structure through training, and therefore remains effective even in such complicated settings. Importantly, the learning approach avoids the need to construct the likelihood function $P(\vec{x} \mid H_{0,1})$, which is a significant advantage in practical applications.

As α increases, or equivalently as the separation between the two point sources becomes larger, both the learning approach and the likelihood-ratio method eventually approach the performance predicted by the Chernoff bound. We note that in a binary discrimination task, the minimal possible success probability P_{succ} is 50%, corresponding to random guessing. In contrast, the Chernoff bound predicts $P_{\text{succ}} = 1 - \exp(-\text{constant} \times S)$, which can be smaller

than 50%—for instance, by formally taking the sample number $S = 0$. This is expected because the Chernoff bound is an asymptotic bound that applies only when S is sufficiently large. This asymptotic nature explains why both the learning approach and the likelihood-ratio method initially outperform the Chernoff prediction for small α, but converge to the Chernoff scaling as α becomes larger.

In principle, the learning-based approach should not outperform the limit predicted by the Chernoff bound, since the Chernoff bound characterizes the fundamental performance limit of a discrimination task. However, we emphasize that the Chernoff bound is not the complete story for quantifying performance in many discrimination problems. Its limitations already appear in this simple example of distinguishing a single point source from two point sources. To approach the scaling of the exponential index $\varsigma = -\log(1 - P_{\text{succ}})$ predicted by the Chernoff bound, we increase α while keeping the sample number S fixed. When the predicted scaling of $\varsigma = -\log(1 - P_{\text{succ}})$ is finally reached, the success probability P_{succ} is already above 99%. In other words, the Chernoff bound accurately reflects performance only in the regime where the task is almost certain to succeed in this example. In practical applications, however, our attention is not on how quickly P_{succ} increases once it is already above 99%, but on when we can reach a moderate level of performance such as 70% and what strategy should be adopted to achieve it. Such nonasymptotic behavior is clearly not captured by the Chernoff bound in our simulation, and the Chernoff bound also does not indicate what strategy should be used to reach the desired performance when we face a complex task for which the likelihood ratio method is not easily applicable. By contrast, our learning-based approach provides the total REC as a meaningful figure of merit in the finite-sample regime, and, more importantly, offers a principled strategy for tackling discrimination tasks when sample number S is finite.

For more complex tasks such as the face recognition example in the main text, it becomes difficult to make a direct comparison with the likelihood ratio method. We do not know how to construct the likelihood function, which is not feasible for complex imaging tasks in practice. In addition, we are not working in the regime where the success probability approaches 100%, so the Chernoff bound would not provide a meaningful benchmark. Instead, we show in Supplementary Figure 26 the performance obtained from the learning-based approach. In the plot, the exponential index $\varsigma = -\log(1 - P_{\text{succ}})$ is shown as a function of α for a fixed sample number, using exactly the same setting as in the main text and in Supplementary Note 8 C. As the success probability depends on the truncation order $\mathcal{K}$, we vary $\mathcal{K}$ and take the peak success probability in this calculation. The calculation shows a step-like structure. Because C_T varies in discrete steps as a function of α, the number of measurable features also grows in a stepwise manner. Correspondingly, both the success probability P_{succ} and the exponential index $\varsigma = -\log(1 - P_{\text{succ}})$ display this step-like behavior as α increases. Importantly, the success probability in this regime is far from 100%, and the learning approach enables us to probe performance well before the asymptotic regime predicted by the Chernoff bound is reached, while also providing a principled strategy for handling complex discrimination tasks in the finite sample regime.

SUPPLEMENTARY REFERENCES

[1] S. L. Braunstein and C. M. Caves, Statistical distance and the geometry of quantum states, Physical Review Letters **72**, 3439 (1994).
[2] M. G. Paris, Quantum estimation for quantum technology, International Journal of Quantum Information **7**, 125 (2009).
[3] M. Tsang, R. Nair, and X.-M. Lu, Quantum theory of superresolution for two incoherent optical point sources, Physical Review X **6**, 031033 (2016).
[4] S. Zhou and L. Jiang, Modern description of rayleigh's criterion, Physical Review A **99**, 013808 (2019).
[5] G. Sorelli, M. Gessner, M. Walschaers, and N. Treps, Optimal observables and estimators for practical superresolution imaging, Physical Review Letters **127**, 123604 (2021).
[6] M. R. Grace, Z. Dutton, A. Ashok, and S. Guha, Approaching quantum-limited imaging resolution without prior knowledge of the object location, Journal of the Optical Society of America A **37**, 1288 (2020).
[7] M. Benedetti, E. Lloyd, S. Sack, and M. Fiorentini, Parameterized quantum circuits as machine learning models, Quantum science and technology **4**, 043001 (2019).
[8] M. Cerezo, A. Arrasmith, R. Babbush, S. C. Benjamin, S. Endo, K. Fujii, J. R. McClean, K. Mitarai, X. Yuan, L. Cincio, *et al.*, Variational quantum algorithms, Nature Reviews Physics **3**, 625 (2021).
[9] M. Schuld, R. Sweke, and J. J. Meyer, Effect of data encoding on the expressive power of variational quantum-machine-learning models, Physical Review A **103**, 032430 (2021).
[10] M. Schuld, A. Bocharov, K. M. Svore, and N. Wiebe, Circuit-centric quantum classifiers, Physical Review A **101**, 032308 (2020).
[11] E. Farhi and H. Neven, Classification with quantum neural networks on near term processors, arXiv preprint arXiv:1802.06002 (2018).
[12] E. Farhi, J. Goldstone, and S. Gutmann, A quantum approximate optimization algorithm, arXiv preprint arXiv:1411.4028 (2014).

[13] Z. Holmes, K. Sharma, M. Cerezo, and P. J. Coles, Connecting ansatz expressibility to gradient magnitudes and barren plateaus, PRX quantum **3**, 010313 (2022).
[14] A. Abbas, D. Sutter, C. Zoufal, A. Lucchi, A. Figalli, and S. Woerner, The power of quantum neural networks, Nature Computational Science **1**, 403 (2021).
[15] R. LaRose and B. Coyle, Robust data encodings for quantum classifiers, Physical Review A **102**, 032420 (2020).
[16] S. Sim, P. D. Johnson, and A. Aspuru-Guzik, Expressibility and entangling capability of parameterized quantum circuits for hybrid quantum-classical algorithms, Advanced Quantum Technologies **2**, 1900070 (2019).
[17] I. Cong, S. Choi, and M. D. Lukin, Quantum convolutional neural networks, Nature Physics **15**, 1273 (2019).
[18] S. Boyd and L. Chua, Fading memory and the problem of approximating nonlinear operators with volterra series, IEEE Trans. Comput.-Aided Des. Integr. Circuits Syst. **32**, 1150 (1985).
[19] G. Tanaka, T. Yamane, J. B. Héroux, R. Nakane, N. Kanazawa, S. Takeda, H. Numata, D. Nakano, and A. Hirose, Recent advances in physical reservoir computing: A review, Neural Netw. **115**, 100 (2019).
[20] P. Mujal, R. Martínez-Peña, J. Nokkala, J. García-Beni, G. L. Giorgi, M. C. Soriano, and R. Zambrini, Opportunities in quantum reservoir computing and extreme learning machines, Adv. Quantum Technol. **4**, 2100027 (2021).
[21] C. M. Wilson, J. S. Otterbach, N. Tezak, R. S. Smith, A. M. Polloreno, P. J. Karalekas, S. Heidel, M. S. Alam, G. E. Crooks, and M. P. da Silva, Quantum kitchen sinks: An algorithm for machine learning on near-term quantum computers, arXiv:1806.08321 (2018).
[22] J. García-Beni, G. L. Giorgi, M. C. Soriano, and R. Zambrini, Scalable photonic platform for real-time quantum reservoir computing, Phys. Rev. Appl. **20**, 014051 (2023).
[23] V. Havlíček, A. D. Córcoles, K. Temme, A. W. Harrow, A. Kandala, J. M. Chow, and J. M. Gambetta, Supervised learning with quantum-enhanced feature spaces, Nature (London) **567**, 209 (2019).
[24] G. E. Rowlands, M.-H. Nguyen, G. J. Ribeill, A. P. Wagner, L. C. G. Govia, W. A. S. Barbosa, D. J. Gauthier, and T. A. Ohki, Reservoir computing with superconducting electronics, arXiv:2103.02522 (2021).
[25] X. Lin, Y. Rivenson, N. T. Yardimci, M. Veli, Y. Luo, M. Jarrahi, and A. Ozcan, All-optical machine learning using diffractive deep neural networks, Science **361**, 1004 (2018).
[26] S. Pai, Z. Sun, T. W. Hughes, T. Park, B. Bartlett, I. A. D. Williamson, M. Minkov, M. Milanizadeh, N. Abebe, F. Morichetti, A. Melloni, S. Fan, O. Solgaard, and D. A. B. Miller, Experimentally realized in situ backpropagation for deep learning in photonic neural networks, Science **380**, 398 (2023).
[27] J. Dambre, D. Verstraeten, B. Schrauwen, and S. Massar, Information processing capacity of dynamical systems, Scientific reports **2**, 514 (2012).
[28] F. C. Sheldon, A. Kolchinsky, and F. Caravelli, Computational capacity of lrc, memristive and hybrid reservoirs, Phys. Rev. E **106**, 045310 (2022).
[29] Y. Wu, J. Yao, P. Zhang, and H. Zhai, Expressivity of quantum neural networks, Phys. Rev. Res. **3**, L032049 (2021).
[30] F. Hu, G. Angelatos, S. A. Khan, M. Vives, E. Türeci, L. Bello, G. E. Rowlands, G. J. Ribeill, and H. E. Türeci, Tackling sampling noise in physical systems for machine learning applications: Fundamental limits and eigentasks, Physical Review X **13**, 041020 (2023).
[31] M. Cerezo, M. Larocca, D. García-Martín, N. L. Diaz, P. Braccia, E. Fontana, M. S. Rudolph, P. Bermejo, A. Ijaz, S. Thanasilp, *et al.*, Does provable absence of barren plateaus imply classical simulability? or, why we need to rethink variational quantum computing, arXiv preprint arXiv:2312.09121 (2023).
[32] M. Cerezo, A. Sone, T. Volkoff, L. Cincio, and P. J. Coles, Cost function dependent barren plateaus in shallow parametrized quantum circuits, Nature communications **12**, 1791 (2021).
[33] J. R. McClean, S. Boixo, V. N. Smelyanskiy, R. Babbush, and H. Neven, Barren plateaus in quantum neural network training landscapes, Nature communications **9**, 4812 (2018).
[34] J. W. Goodman, *Introduction to Fourier optics* (Roberts and Company publishers, 2005).
[35] L. B. Lucy, Statistical limits to super resolution, Astronomy and Astrophysics, Vol. 261, NO. 2/AUGI, P. 706, 1992 **261**, 706 (1992).
[36] Y. Wang and V. O. Lorenz, Fundamental limit of bandwidth-extrapolation-based superresolution, Physical Review A **108**, 012602 (2023).
[37] M. P. Lavery, D. J. Robertson, G. C. Berkhout, G. D. Love, M. J. Padgett, and J. Courtial, Refractive elements for the measurement of the orbital angular momentum of a single photon, Optics express **20**, 2110 (2012).
[38] M. W. Beijersbergen, L. Allen, H. Van der Veen, and J. Woerdman, Astigmatic laser mode converters and transfer of orbital angular momentum, Optics Communications **96**, 123 (1993).
[39] R. Ionicioiu, Sorting quantum systems efficiently, Scientific reports **6**, 25356 (2016).
[40] Y. Zhou, M. Mirhosseini, D. Fu, J. Zhao, S. M. Hashemi Rafsanjani, A. E. Willner, and R. W. Boyd, Sorting photons by radial quantum number, Physical review letters **119**, 263602 (2017).
[41] Y. Zhou, J. Zhao, Z. Shi, S. M. Hashemi Rafsanjani, M. Mirhosseini, Z. Zhu, A. E. Willner, and R. W. Boyd, Hermite–gaussian mode sorter, Optics letters **43**, 5263 (2018).
[42] R. A. Horn and C. R. Johnson, *Matrix analysis* (Cambridge university press, 2012).
[43] S. Z. Ang, R. Nair, and M. Tsang, Quantum limit for two-dimensional resolution of two incoherent optical point sources, Physical Review A **95**, 063847 (2017).
[44] C. Lupo, Z. Huang, and P. Kok, Quantum limits to incoherent imaging are achieved by linear interferometry, Physical Review Letters **124**, 080503 (2020).
[45] Z. Yu and S. Prasad, Quantum limited superresolution of an incoherent source pair in three dimensions, Physical review letters **121**, 180504 (2018).

[46] S. Pirandola, B. R. Bardhan, T. Gehring, C. Weedbrook, and S. Lloyd, Advances in photonic quantum sensing, Nature Photonics **12**, 724 (2018).
[47] M. Tsang, Quantum limit to subdiffraction incoherent optical imaging, Physical Review A **99**, 012305 (2019).
[48] M. Tsang, Subdiffraction incoherent optical imaging via spatial-mode demultiplexing, New Journal of Physics **19**, 023054 (2017).
[49] Y. Wang, Y. Zhang, and V. O. Lorenz, Superresolution in interferometric imaging of strong thermal sources, Physical Review A **104**, 022613 (2021).
[50] R. Nair and M. Tsang, Far-field superresolution of thermal electromagnetic sources at the quantum limit, Physical review letters **117**, 190801 (2016).
[51] C. Lupo and S. Pirandola, Ultimate precision bound of quantum and subwavelength imaging, Physical review letters **117**, 190802 (2016).
[52] C. Napoli, S. Piano, R. Leach, G. Adesso, and T. Tufarelli, Towards superresolution surface metrology: quantum estimation of angular and axial separations, Physical review letters **122**, 140505 (2019).
[53] F. Yang, A. Tashchilina, E. S. Moiseev, C. Simon, and A. I. Lvovsky, Farfield linear optical superresolution via heterodyne detection in a higher-order local oscillator mode, Optica **3**, 1148 (2016).
[54] Z. S. Tang, K. Durak, and A. Ling, Fault-tolerant and finite-error localization for point emitters within the diffraction limit, Opt. Express **24**, 22004 (2016).
[55] M. Paúr, B. Stoklasa, Z. Hradil, L. L. Sánchez-Soto, and J. Rehacek, Achieving the ultimate optical resolution, Optica **3**, 1144 (2016).
[56] W.-K. Tham, H. Ferretti, and A. M. Steinberg, Beating rayleigh's curse by imaging using phase information, Phys. Rev. Lett. **118**, 070801 (2017).
[57] M. Parniak, S. Borówka, K. Boroszko, W. Wasilewski, K. Banaszek, and R. Demkowicz-Dobrzanski, Beating the rayleigh limit using two-photon interference, Phys. Rev. Lett. **121**, 250503 (2018).
[58] U. Zanforlin, C. Lupo, P. W. Connolly, P. Kok, G. S. Buller, and Z. Huang, Optical quantum super-resolution imaging and hypothesis testing, Nature Communications **13**, 5373 (2022).
[59] L. Santamaria, F. Sgobba, and C. Lupo, Single-photon sub-rayleigh precision measurements of a pair of incoherent sources of unequal intensity, Optica Quantum **2**, 46 (2024).
[60] X.-J. Tan, L. Qi, L. Chen, A. J. Danner, P. Kanchanawong, and M. Tsang, Quantum-inspired superresolution for incoherent imaging, Optica **10**, 1189 (2023).
[61] C. Rouvière, D. Barral, A. Grateau, I. Karuseichyk, G. Sorelli, M. Walschaers, and N. Treps, Ultra-sensitive separation estimation of optical sources, Optica **11**, 166 (2024).
[62] Z. Huang and C. Lupo, Quantum hypothesis testing for exoplanet detection, Physical Review Letters **127**, 130502 (2021).
[63] H. Zhang, S. Kumar, and Y.-P. Huang, Super-resolution optical classifier with high photon efficiency, Optics Letters **45**, 4968 (2020).
[64] X.-M. Lu, H. Krovi, R. Nair, S. Guha, and J. H. Shapiro, Quantum-optimal detection of one-versus-two incoherent optical sources with arbitrary separation, npj Quantum Information **4**, 64 (2018).
[65] M. R. Grace and S. Guha, Identifying objects at the quantum limit for superresolution imaging, Physical Review Letters **129**, 180502 (2022).
[66] K. M. Audenaert, J. Calsamiglia, R. Munoz-Tapia, E. Bagan, L. Masanes, A. Acin, and F. Verstraete, Discriminating states: The quantum chernoff bound, Physical review letters **98**, 160501 (2007).
[67] H. Chernoff, A measure of asymptotic efficiency for tests of a hypothesis based on the sum of observations, The Annals of Mathematical Statistics , 493 (1952).
[68] T. M. Cover, *Elements of information theory* (John Wiley & Sons, 1999).
